# Using Administrative Data to Predict Suicide After Psychiatric Hospitalization in the Veterans Health Administration System

**DOI:** 10.3389/fpsyt.2020.00390

**Published:** 2020-05-06

**Authors:** Ronald C. Kessler, Mark S. Bauer, Todd M. Bishop, Olga V. Demler, Steven K. Dobscha, Sarah M. Gildea, Joseph L. Goulet, Elizabeth Karras, Julie Kreyenbuhl, Sara J. Landes, Howard Liu, Alex R. Luedtke, Patrick Mair, William H. B. McAuliffe, Matthew Nock, Maria Petukhova, Wilfred R. Pigeon, Nancy A. Sampson, Jordan W. Smoller, Lauren M. Weinstock, Robert M. Bossarte

**Affiliations:** ^1^Deparment of Health Care Policy, Harvard Medical School, Boston, MA, United States; ^2^Department of Psychiatry, Harvard Medical School, Boston, MA, United States; ^3^Center for Healthcare Organization & Implementation Research, VA Boston Healthcare System, Boston, MA, United States; ^4^Center of Excellence for Suicide Prevention, Canandaigua VA Medical Center, Canandaigua, NY, United States; ^5^Division of Preventive Medicine, Brigham and Women’s Hospital, Boston, MA, United States; ^6^Department of Medicine, Harvard Medical School, Boston, MA, United States; ^7^VA Center to Improve Veteran Involvement in Care, VA Portland Health Care System, Portland, OR, United States; ^8^Department of Psychiatry, Oregon Health & Science University, Portland, OR, United States; ^9^Pain, Research, Informatics, Multimorbidities & Education Center, VA Connecticut Healthcare System, West Haven, CT, United States; ^10^Department of Emergency Medicine, Yale School of Medicine, New Haven, CT, United States; ^11^VA Capitol Healthcare Network (VISN 5), Mental Illness Research, Education, and Clinical Center (MIRECC), Baltimore, MD, United States; ^12^Department of Psychiatry, Division of Psychiatric Services Research, University of Maryland School of Medicine, Baltimore, MD, United States; ^13^South Central Mental Illness Research Education Clinical Center (MIRECC), Central Arkansas Veterans Healthcare System, North Little Rock, AR, United States; ^14^Department of Psychiatry, University of Arkansas for Medical Sciences, Little Rock, AR, United States; ^15^Department of Statistics, University of Washington, Seattle, WA, United States; ^16^Vaccine and Infectious Disease Division, Fred Hutchinson Cancer Research Center, Seattle, WA, United States; ^17^Department of Psychology, Harvard University, Cambridge, MA, United States; ^18^Department of Psychiatry, University of Rochester Medical Center, Rochester, NY, United States; ^19^Department of Psychiatry, Massachusetts General Hospital, Boston, MA, United States; ^20^Department of Psychiatry & Human Behavior, Alpert Medical School of Brown University, Providence, RI, United States; ^21^West Virginia University Injury Control Research Center and Department of Behavioral Medicine and Psychiatry, West Virginia University School of Medicine, Morgantown, WV, United States

**Keywords:** intensive case management, machine learning, predictive analytics, suicide, super learner

## Abstract

There is a very high suicide rate in the year after psychiatric hospital discharge. Intensive postdischarge case management programs can address this problem but are not cost-effective for all patients. This issue can be addressed by developing a risk model to predict which inpatients might need such a program. We developed such a model for the 391,018 short-term psychiatric hospital admissions of US veterans in Veterans Health Administration (VHA) hospitals 2010–2013. Records were linked with the National Death Index to determine suicide within 12 months of hospital discharge (n=771). The Super Learner ensemble machine learning method was used to predict these suicides for time horizon between 1 week and 12 months after discharge in a 70% training sample. Accuracy was validated in the remaining 30% holdout sample. Predictors included VHA administrative variables and small area geocode data linked to patient home addresses. The models had AUC=.79–.82 for time horizons between 1 week and 6 months and AUC=.74 for 12 months. An analysis of operating characteristics showed that 22.4%–32.2% of patients who died by suicide would have been reached if intensive case management was provided to the 5% of patients with highest predicted suicide risk. Positive predictive value (PPV) at this higher threshold ranged from 1.2% over 12 months to 3.8% per case manager year over 1 week. Focusing on the low end of the risk spectrum, the 40% of patients classified as having lowest risk account for 0%–9.7% of suicides across time horizons. Variable importance analysis shows that 51.1% of model performance is due to psychopathological risk factors accounted, 26.2% to social determinants of health, 14.8% to prior history of suicidal behaviors, and 6.6% to physical disorders. The paper closes with a discussion of next steps in refining the model and prospects for developing a parallel precision treatment model.

## Introduction

Suicide is the 10^th^ leading cause of death in the US (1). The suicide rate has increased steadily since 1999 (2) and especially among veterans (3). Transitions in care, especially psychiatric hospital discharge, are periods of particularly high suicide risk in the general population, including veterans (4, 5). Indeed, the approximately 1% of Veteran Health Administration (VHA) patients who are hospitalized for psychiatric disorders each year account for nearly 12% of all VHA suicides over the subsequent 12 months (6). Programs to reduce suicides after psychiatric hospital discharge are urgently needed. Beginning in 2008, VHA implemented a series of suicide prevention recommendations that addressed this need by requiring each VHA treatment facility to appoint a suicide prevention coordinator (7) and, more recently, to require inpatient clinicians to develop a suicide safety plan with each inpatient before discharge (8). These changes were associated with a stabilization of the previously rising postdischarge suicide rate, although the rate is still very high compared to others (6).

Although several VHA outpatient treatment programs exist, none is designed specifically for patients at high suicide risk after psychiatric hospital discharge. Intensive postdischarge case management programs, which are not used in VHA, have been shown elsewhere to be effective in reducing suicides after psychiatric hospital discharge (9–16), leading to recommendations to add such programs to existing postdischarge suicide preventive interventions (17). However, these programs can be labor-intensive, requiring frequent outpatient contacts, assertive outreach for missed outpatient appointments, and intensive community support to engage reluctant patients. It would be difficult to justify implementing such a program for all VHA patients given the rarity of postdischarge suicides (about 3/1,000 hospitalizations) (6) and the scarcity of the specially trained staff needed to implement this type of intervention in each of the nearly 100 VHA psychiatric inpatient units and over 1,000 outpatient clinics to which inpatients are discharged around the country (9).

Such a program would be more scalable, though, if it focused on recently discharged patients at high suicide risk and was implemented remotely by centralized program staff to increase efficiency. We are in the process of piloting a promising program of this sort known as the Coping Long Term with Active Suicide Program, a telephone-based adjunctive intensive case management program that has been shown to have significant aggregate effects on suicide-related behaviors (SRBs) after psychiatric hospital discharge (9, 18, 19). A first step in implementing this kind of targeted intervention would be to develop a predictive analytic model that pinpoints the inpatients with high risk of subsequent suicide. A model of this sort was developed for hospitalized US Army soldiers as part of the Army STARRS research program (20). More than 50% of all suicides in the 12 months after hospital discharge in that study occurred among the 10% of soldiers defined as being at highest risk. However, a much more extensive set of predictor variables was available to build this model in the integrated Department of Defense administrative data system than exists to build a VHA model, making it unclear if a comparable model could be developed in VHA. We present the results of a preliminary effort to investigate this question in the current report.

## Materials and Methods

### Sample

We focus on the 391,018 short-term psychiatric hospital admissions of veterans in any VHA hospital in the US or its territories (i.e., American Samoa, Guam, Northern Mariana Islands, Puerto Rico, and US Virgin Islands) during calendar years 2010–2013. Records of these visits were linked with the National Death Index (21) to determine which patients died within 12 months of hospital discharge. The analysis sample was drawn from a master sample we developed that was a variation of the case-control approach used in our previous research (20). The cases in this master sample were 100% of the VHA patients who died in calendar years 2010–2014 and were last seen in the VHA system within 2 years (24 months) of their death and were classified in the National Death Index as dying either by suicide, by any opioid-related cause, or by drug overdose. This case definition excludes deaths by other external cause (i.e., murder or other accidents), some of which might have been misclassified suicides.

The controls were a stratified probability sample of other patients ever seen in the VHA system in calendar years 2008–2013 (i.e., within 2 years of any month in the time period 2010–2013). The stratification scheme for controls was hierarchical and included (i) patients that made a suicide attempt recorded in the VHA administrative records; (ii) other patients (i.e., exclusive of those that made a suicide attempt) that had a psychiatric hospitalization; (iii) other patients that had any outpatient psychiatric treatment; and (iv) all other patients. Sampling fractions within strata were set to generate a sample of controls either four times the number of cases in the stratum (in stratum i), three times the number of cases (in strata ii-iii), or two times the number of cases (in stratum iv). Sampling of controls was carried out using secondary stratification for discharge date and a variety of socio-demographic and clinical factors. This differential sampling was designed to increase power in the segments of the population where suicide rates are highest under a financial constraint on total number of controls because we purchased some of the predictor data from a commercial data aggregation firm.

For the analysis of suicides after psychiatric hospital discharge, the unweighted case-control ratio was about 1:11.5 at the person level. However, as we included all hospitalizations for all patients in the case-control sample, the case-control ratio at the level of the hospitalization was somewhat different (1:17). The person-level data were weighted by the inverse of their probabilities of selection for purposes of analysis and population projection. The 70% of case hospitalizations with the earliest discharge dates were combined with all control hospitalizations up to the same discharge date to create a training sample in which the prediction model was developed. The remaining 30% of cases and controls were held out to validate the model. The study protocol was approved by Research Ethics Committee of the Veterans Administration Center of Excellence for Suicide Prevention and Harvard Medical School with a waiver of informed consent based on the data being deidentified.

### Predictors

#### Overview

We turned to prior studies of data from electronic health records to determine the predictor set. Troister et al. (22) carried out a comprehensive review of published studies of risk factors for civilian postdischarge suicides as of 2006 and found five replicated classes of predictors: (i) history of prior suicidal behaviors; (ii) psychopathological disorders (the most consistent being nonaffective psychosis, mood disorders, and multiple comorbid psychiatric disorders), medications for these disorders, and interactions between specific psychopathological disorders and medications known to be especially useful in protecting against suicide among patients with these disorders [e.g., lithium among patients with bipolar disorder; (23)]; (iii) quality of care after hospital discharge (e.g., low continuity of care); (iv) time since hospital discharge (inversely related to suicide risk); and (v) socio-demographics (the most consistent being male gender and recent job loss), which more recently have been conceptualized as indicators of social determinants of health (24, 25). Studies published after the Troister et al. review found similar predictors (20, 26–28). We included indicators of all these predictor classes in our analysis.

We also included two additional predictor sets that could be considered indicators of social determinants of health: (vi) International Classification of Diseases, Ninth Revision, Clinical Modification [**ICD-9-CM**; (29)] E and V codes for social factors known to be associated with suicide, such as sexual assault victimization (30) and financial loss (31); and (vii) small-area geocode data (e.g., neighborhood deprivation, local unemployment rate). And we looked at potentially informative interactions between patient socio-demographics and neighborhood characteristics (e.g., Black patients living in predominantly White neighborhoods). Finally, we included indicators of 3 other predictor classes found in more general studies of suicides among outpatients and health plan members: (viii) physical disorders and medications for the treatment of these disorders (32); (ix) medications that are thought to be associated with increased suicide risk (33); and (x) medical procedures associated either with increased suicide risk [e.g., amputations; (34)] or decreased suicide risk [e.g., certain types of psychotherapy; (35)].

#### Data Sources

Three VHA data systems were used to operationalize most of the predictors:

- *The VHA Corporate Data Warehouse* [CDW; (36)]: An integrated system containing data on patient socio-demographics along with information on all health care encounters either in VHA or paid for by VHA in the community, classified in terms of primary and secondary ICD-9-CM diagnostic and procedure codes. The CDW also contains information on prescriptions written in VHA or otherwise paid for by VHA, classified using the VHA Drug Classification System (37). The CDW also includes a comprehensive list of test results along with E codes for external causes of injury due to accidents, suicide attempts, and other types of self-inflicted injuries and V codes for other factors influencing health status and contact with the health care system that contain information about social determinants of health (38);
- *The Veterans Administration Suicide Prevention Applications Network* (39): This is an administrative data system for suicide behavior tracking in VHA;
- *The Veterans Administration Homes Registry*: The Homes Registry is a data system maintained by the National Center on Homelessness Among Veterans (40) that includes information on all veterans known either to be homeless, at risk of homelessness, or in a VHA homelessness program. For the current analysis, though, homelessness was determined by ICD-9-CM codes, Patient Treatment File (PTF) Inpatient Codes, and outpatient stop codes.

We augmented the information obtained in the three VHA data systems with small area geocode data available from various government databases at the levels of the Census Block Group, Census Tract, or County to characterize neighborhood socio-demographic profiles and social factors either known or suspected to be associated with suicide risk. Information on patient home address from the CDW was used to link patient records to these geocode data systems.

The small amount of missing values found in this data often were nonmissing in earlier records, allowing nearest neighbor imputations. Remaining missing values and inconsistencies were reconciled using rational imputations (e.g., a patient classified as female in one record but male in both earlier and later records was recoded male). Details about missing data patterns are available in **Supplementary Tables 1** and **2**.

#### Predictor Classes

- *History of prior suicidal behaviors:* ICD-9-CM E and V codes (see **Supplementary Table 3**) and the Veterans Administration Suicide Prevention Applications Network system provided information on history of suicidal ideation and attempts reported in inpatient, outpatient, or emergency department visits, including as a basis for the current hospitalization.
- *Psychopathological risk factors:* We created 3 variables for each of 7 retrospective time periods (past 30, 90, 180, 365, 730, 1,095 days and the veterans’ entire VHA history as of January 1, 2000) for each of the 582 diagnoses or diagnostic groupings of mental disorders in the ICD-9-CM and each of the 41 mental disorder diagnoses in the Clinical Classification Software (41): yes/no for any visit with this diagnosis during the time period; a continuous count of number of days with such visits; and a stabilized 0–4 quintile transformation of the latter count. We also created a series of composite variables for common types of comorbidity among these disorders, including comorbidities thought to predict suicide that involve a combination of mental and physical disorders [e.g., (42–44)]. ICD-9-CM codes and details about each disorder are presented in **Supplementary Table 4**.
  We also included information about medications used to treat the above disorders obtained from the VHA National Formulary. The latter is a three-level classification system that includes a total of 574 categories (32 major drug classes, 287 minor drug classes, 255 subclasses) to characterize the 29,290 individual pharmaceutical products available through VHA. We created count variables for prescriptions filled for the Central Nervous System class (of the 32 major drug classes) as well as for each of the minor and subclasses of this class in the 90 days and 365 days prior to the focal hospital admission. In addition, we created interaction terms to define the conjunction of two broad mental disorder diagnosis groups, schizophrenic psychoses and affective psychoses, with medications found to be associated with reduced suicide among patients with these disorders: clozapine, olanzapine, and quetiapine along with long-acting injectable antipsychotics for schizophrenic psychoses (45–47); and lithium for affective psychoses (23). And we included a count of the number of medications used to help offset the extrapyramidal side effects of antipsychotics that can contribute to suicidality (48) in the 90 days and 365 days prior to the focal hospital admission. See **Supplementary Table 4** for the complete list of medications.
- *Quality of care and aftercare for psychiatric inpatients:* Recent research in the UK has found that quality of care indicators, such as extent of staff turnover and short average duration of stay, are significant predictors of postdischarge suicides (5, 14). Only superficial indicators of this sort (e.g., driving time between the patient’s home and the nearest VHA treatment center) were included in the initial model-building exercise reported here, as the more comprehensive facility-level indicators we are developing were not ready at the time of analysis.
- *Time since hospital discharge:* As noted above in the section on analysis methods, we developed separate models for five risk time horizons: 1 week and 1, 3, 6, and 12 months since hospital discharge (22, 49).
- *Socio-demographics:* The CDW provided information on patient age, sex, race/ethnicity, marital status, income, religion, residential characteristics (Census Division, urbanicity, homelessness), and period of service (pre-Vietnam, Vietnam era, post-Vietnam, Persian Gulf War) (**Supplementary Table 5**).
- *ICD-9-CM E and V codes:* The latter codes were used to count information on 14 different types of accidents, physical and sexual assaults, and perpetration of child or adult abuse (**Supplementary Table 6**). We also coded medical encounters due to housing or economic circumstances and due to other family or psychosocial circumstances (38).
- *Small-area geocode data:* Annual rolling 5-year average data at the levels of the Census Block Group or Census Tract were obtained from the American Community Survey (50) on a wide range of small area characteristics that previous research has shown to cluster into two dimensions associated with variation in suicide rates: neighborhood deprivation (22 indicators; e.g., low median education, high unemployment and poverty rates, percent of households receiving public assistance) and neighborhood fragmentation (5 indicators: proportions of households with single-person occupant, vacant, occupant unmarried, occupant residing in the housing unit less than 12 months, occupant owns the housing unit) (51–54). American Community Survey data were also obtained on neighborhood race/ethnicity. Based on evidence that individual-neighborhood differences sometimes predict suicides (55, 56), we created interactions of patient race/ethnicity with the percent of neighborhood residents who were of the same race/ethnicity (non-Hispanic Black, non-Hispanic White, Hispanic, Other), a ratio of patient income to median neighborhood income, and an indicator for the percent of neighborhood residents who were veterans. Based on evidence that food insufficiency is more important than low income in predicting suicidality (57), data were obtained from the Department of Agriculture on the percent of the neighborhood that was a food desert (58). We also obtained information on the County-level suicide rate averaged over the past 2 most recently available years (59). Finally, based on evidence that economic trends are associated with trends in suicide rates (60), we obtained County-level data on the bankruptcy rate, the median debt-to-income ratio, and the unemployment rate averaged over the past 3–14 months. We also calculated changes in these statistics over the past three months or, for bankruptcy, past 2 years compared to the 3 years before that (**Supplementary Table 7**).
- *Physical disorders:* The Clinical Classification Software system was used to organize information about the roughly 13,000 ICD-9-CM diagnoses into a 646-variable 4-level hierarchical system. We created the same three variables at the seven retrospective time periods for each of these 646 variables as we did for the psychopathological risk factors, resulting in 13,566 variables about individual physical disorders. In addition, we created a series of composite measures for types of comorbidity reported in the literature (**Supplementary Table 8**) as potentially important predictors of suicide (61–64).
  We also included information about medications used to treat physical disorders from the VHA National Formulary. As noted above, the latter is a three-level classification system that includes a total of 32 major drug classes to characterize the 29,290 individual pharmaceutical products available through VHA. In addition to the count variables noted above for all three levels of Central Nervous System drugs, we created count variables for prescriptions filled for each of the other 31 major nonpsychotropic drug classes in the 90 days and 365 days prior to the focal hospital admission.
- *Medications thought to cause suicide:* Literature suggests that some medications for physical disorders might predispose to suicide (65, 66). In order to investigate this possibility, we searched Food and Drug Administration approved drug labeling documents in the Food and Drug Administration Label Database (67) using the search terms *suicidality, suicidal behavior, suicidal ideation, suicide attempt, suicidal*, and *suicide* and found 49 medications that indicated suicide as an adverse reaction in the box warning section of the drug label, 112 that included suicide in the warnings and precautions section, and 79 that included suicide in the adverse reactions section. We created separate count variables for each of these three levels of possible risks to describe prescriptions in the 90 days and 365 days prior to the focal hospital admission (**Supplementary Table 9**).
- *Medical procedures:* The CDW uses ICD-9-CM procedure codes to record inpatient procedures and the American Medical Association Current Procedural Terminology codes to recode outpatient procedures. We included measures for a mix of ranges and specific procedures within each system for each of the same seven retrospective time periods used to code diagnoses (**Supplementary Table 10**).

### Analysis Methods

Numerous recent studies predicted suicide death or attempted suicide in high-risk patient populations from electronic health record data using machine learning (ML) methods. These studies focused either on psychiatric inpatients after hospital discharge (20), psychiatric outpatients after recent visits (68), or emergency department patients deemed to need a suicide risk assessment (69, 70). Results showed that ML methods have considerable promise even though all these studies were limited in a number of ways discussed elsewhere (71). We attempted to build on these prior studies by introducing five improvements:

- Rather than choose only one or compare across a small number of alternative ML classifiers, we used the Super Learner (**SL**) ensemble ML method to combine predicted probabilities of suicide at the level of hospitalization across a large number of different ML algorithms (the “ensemble”) (72). This is an important improvement over previous studies because no single ML algorithm is universally optimal. SL has a guarantee to be at least as accurate and typically has a considerably higher level of prediction accuracy than the best-performing algorithm in the ensemble. Following recent recommendations (73), we used a wide range of algorithms in the ensemble to optimize performance (**Table 1**). These included a generalized linear model with a logistic link function, a series of penalized regressions with different mixing model parameters, a series of support vector machines with different kernels, Bayesian adaptive regression trees, neural networks, random forest, and a series of gradient boosted decision trees that differed in depth and shrinkage.
- We used three different feature selection methods: univariate p value less than .10; and, within this set, Least Absolute Shrinkage and Selection Operator regression and random forest (see **Table 1** for descriptions) to reduce the number of potential predictors included in SL. This kind of initial feature pruning can improve out-of-sample model performance substantially (80).
- A number of the algorithms in our SL library require hyper-parameter tuning for optimal performance. We addressed this problem in simple cases by including a series of models for a single algorithm with different hyper-parameter values in the SL ensemble (e.g., five penalized regression classifiers that differed in values of the mixing parameter, several different support vector machines that differed in kernels). In more complex cases we used the random search method in the Classification and Regression Training package to select optimal hyper-parameter values separately for each time horizon (81).
- Suicide is a very rare outcome even among recently discharged psychiatric inpatients, with a case-control ratio of about 3:1,000. This kind of extreme class imbalance can pose problems for estimation because most algorithms aim to optimize overall classification accuracy and fail to adjust for the fact that false negatives may be more costly than false positives, leading the algorithms to focus on correctly classifying the much more common noncases at the expense of misclassifying the rare cases (82). A number of strategies involving under-sampling controls, pseudo-sampling cases, and combinations have been developed to address this problem (83) and shown to improve model performance [e.g., (84, 85)]. We did this by making five copies of each case record and subsampling an equal number of control records using stratified probability sampling to create a balanced dataset for estimation. Once the model was estimated and predicted probabilities of suicide were assigned to each record, we reweighted each record in the balanced dataset by the inverse of its probability of selection to recover true unit-level predicted probabilities of suicide. We then used these weight-corrected estimates to evaluate model fit in the training sample. Five-fold cross-validation was used for internal SL cross-validation both to build optimal models with each classifier and to determine optimal weighting across classifiers in the ensemble. All five replicates of a stratified 20% of case records were included in a single five-fold cross-validation fold in order to address the problem that overfitting can occur when cases are duplicated.
- Given that the time horizon for intervention can vary substantially depending on whether the concern is with imminent risk (e.g., suicide shortly after hospital discharge) or subsequent readjustment back into the community (e.g., suicide within 12 months of hospital discharge), we built separate models for each of 5 risk time horizons: 1 week and 1, 3, 6, and 12 months after hospital discharge.

**Table 1:** Overview of the algorithms used in the Super Learner ensemble.

Algorithm	R package	Description
Logistic regression	stats	Traditional parametric logistic regression Prone to overfit if independent variables are highly collinear Optimal functional form of independent variables unknown (e.g., linear versus nonlinear)
Elastic net regularization (74)	glmnet	Penalized regression reduces overfit due to collinear independent variables Ridge regression shrinks coefficients for collinear independent variables toward zero, but does not fully-eliminate any independent variable Elastic net regression allows various penalties where coefficients for collinear independent variables are shrunk toward zero (but not eliminating contributions to the predicted probability) and/or to zero (eliminating their contributions to the predicted probability) Mixing parameter penalty (alpha) is set somewhere between .01 and .99 Least Absolute Shrinkage and Selection Operator (LASSO) regression shrinks coefficients for collinear covariate coefficients to zero, eliminating their contributions to the predicted probability
Random forest decision trees (75)	ranger	Decision tree methods capture interactions and non-linear associations Independent variables are partitioned (based on values) and stacked to build decision trees and ensemble an aggregate “forest” Random forest builds numerous trees in bootstrapped samples and generates an aggregate tree by averaging across trees (reducing overfit) Suitable for large data sets, but may be unstable and overfitting
Bayesian additive regression trees (76)	bartMachine	Bayesian trees are based on an underlying probability model (priors) for the structure and likelihood for data in terminal nodes The aggregate tree is generated by averaging across tree posteriors (reducing overfit)
Extreme gradient boosting (77)	xgboost	Extreme gradient boosting decision tree algorithm Final predictions are formulated by models sequentially built (using gradient descent algorithm to minimize loss) to resolve residual error made by existing models
Support vector machines (78)	ksvm	Support vector machines treats independent variables as dimensions in high dimensional space and attempts to identify the best hyperplane to separate the sample into classes (e.g., cases and noncases) Goal is to find the hyperplane with the maximum margin between the two closest points in space Captures linear associations, but alternate kernels can be used to capture nonlinearities (polynomial and radial basis kernels were used here)
Linear kernel		
Polynomial kernel		
Radial kernel		
Neural networks (79)	nnet	Connections between predictors and the outcome are modeled as a network Predictors affect the outcome through intermediate layers
		Weights are assigned to connections Capture interactions and non linear associations Low interpretability

Standard evaluations of model performance were used in the test sample. We began by generating the receiver operating characteristic (ROC) curves and then calculating area under the receiver operating characteristic curve (AUC) for the SL model developed to predict suicides over each time horizon. Each of the five SL models was used to predict suicides over each of the five thresholds in the holdout sample to determine if prediction accuracy for shorter thresholds than 12 months would be improved by developing models for each threshold rather than only developing a model to predict suicides over all 12 months.

We then calculated operating characteristics, including sensitivity (the proportion of suicide cases that were above a given prediction threshold), specificity (the proportion of suicide noncases that were below the same prediction threshold) and positive predictive value (PPV; the probability of suicide above the decision threshold within the time horizon) for a variety of thresholds. The latter included the 5%, 10%, 20%, and 60% of observations with highest predicted probabilities of suicide based on the model as well as the thresholds needed to achieve fixed values of sensitivity and specificity. We then calculated a modification of PPV designed to adjust for differences across time horizons by computing the number of suicides per 100 patient-years rather than per 100 patients. Adjusted PPV is of interest because it allows us to estimate the expected number of patients who would otherwise die by suicide *over the time horizon* a clinician would work with under alternative scenarios in which the clinician either treated a larger number of patients over a shorter period of time or treated a smaller number of patients over a longer period of time. This is a useful distinction because conditional suicide risk is much higher in the first weeks and months after hospital discharge. This is difficult to see when focusing on a conventional PPV measure, as the latter increases as the time horizon increases. However, adjusted PPV decreases as the time horizon increases when conditional suicide risk decreases in this same way.

We then investigated which predictor variables were of greatest overall importance by using the Extreme Gradient Boosting ensemble decision tree algorithm to predict SL predicted probabilities of suicide for each time horizon (77). The importance of the splitting variable at each node of each tree was determined by examining the extent to which prediction performance at the node changed when the splitting variable was replaced by random noise. This importance measure for each split was then weighted by the proportion of the sample involved in the split and these predictor-specific weighted importance measures were summed across all nodes of all trees to arrive at a summary measure of “gain” in model prediction accuracy due to each predictor variable (86). The sum of gain across predictors was normed to 1.0. We then grouped predictors by the 10 broad categories of predictors described above.

## Results

### Outcome Distribution

There were 771 suicides among the 195,349 veterans who were hospitalized by VHA for a psychiatric problem during calendar years 2010–2013 and who died in the 365 days after discharge. These 771 veterans had 1,195 psychiatric hospitalizations in 2010–2013 out of the 391,018 such hospitalizations during this time period. As noted in the sample section, we used hospitalization as the unit of analysis, which means that we had 1,195 “cases” (i.e., hospitalizations followed within 12 months by a suicide) and 389,823 controls (i.e., other hospitalizations). The 70% of case hospitalizations with the earliest months of discharge (n=864; January 1, 2010–October 22, 2012) were combined with all control hospitalizations up through the same discharge month to create a training sample in which the prediction model was developed. The remaining 30% of case hospitalizations (n=331) and the associated controls were held out to validate the model.

**Figure 1:**
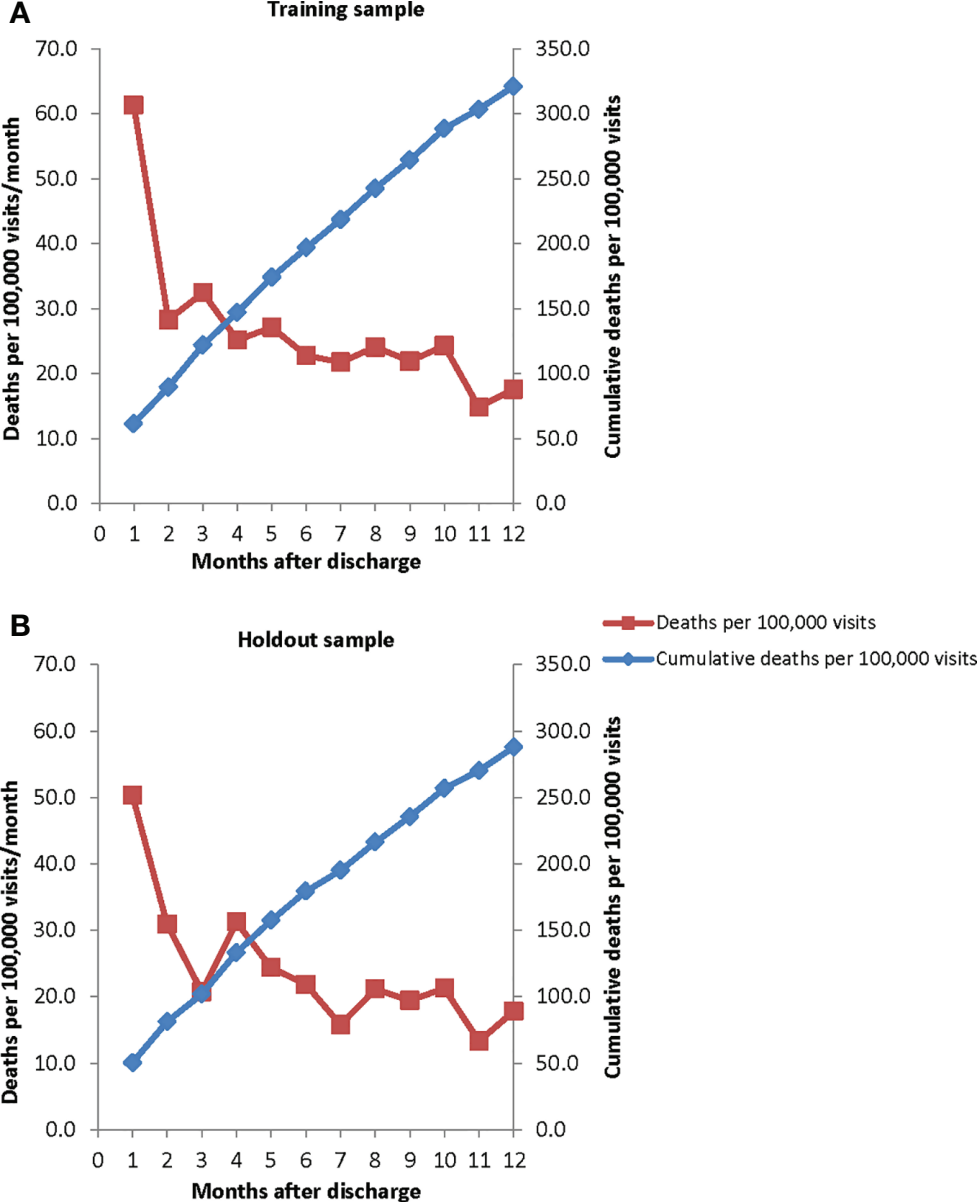
Monthly suicide hazard rates and cumulative incidence rates over the 12 months after psychiatric hospital discharge in **(A)** the training sample (January 1, 2010–October 22, 2012) and **(B)** holdout sample (October 23, 2012–December 31, 2013).

The observed suicide rate at the level of hospitalization over the 12 months after hospital discharge was 315.5 per 100,000 person-years in the training sample (**Figure 1A**) and 282.5 in the holdout sample (**Figure 1B**). In both samples, the suicide rate varied significantly and inversely with time since discharge (χ^2^_11_ = 115.3–51.3, p < .001), with the highest suicide rate in the first week after discharge (1,104.3–1,290.7 per 100,000 person-years), a much lower rate in the remainder of the first month after discharge (626.7–399.9), an even lower rate in the 2–3 months after discharge (363.4–309.3), and a more gradually decreasing rate over subsequent months (from 299.0–308.9 4–6 months after discharge to 246.9–215.8 7–12 months after discharge).

### Stratification Variable Distributions

We noted above that the sample was stratified to match the population of all psychiatric hospital admissions over the study period on the cross-classification of diverse socio-demographic and geographic variables as well as on a measure of whether the patient had a suicide risk flag on their medical record in the month prior to their hospitalization (**Supplementary Table 11**). A discussion of VHA suicide risk flags is presented elsewhere (87). The great majority of patients in both the training sample and the holdout sample were male (92.6%–93.4%) and had a median age of 54. Consistent with this age distribution, the plurality served most recently either in the Vietnam era (37.1%–39.4%), followed by the Persian Gulf War era (29.9%–36.3%) and the years between the Vietnam and Persian Gulf War eras (22.9%–25.1%). The majority were non-Hispanic White (60.1%–61.3%) and others mostly non-Hispanic Black (24.3%–24.9%). The plurality was either divorced (38.4%–38.8%) and the others mostly either married (24.3%–26.0%) or never married (23.0%–23.3%). The majority lived in Census-defined metropolitan statistical areas of more than 1 million residents (51.4%–53.5%) or 250,000–1 million residents (23.1%–23.8%) and only a small proportion (8.0%–9.8%) lived in areas with populations of less than 20,000. The vast majority reported being Christian (72.8%–73.3%) and most of these either Baptists (24.1%–28.2%) or Roman Catholics (18.9%–19.8%), whereas most others (19.9%–21.1%) reported having no religion. A strikingly high 35.6%–41.1% had been homeless at some time in the 12 months before hospitalization, including 16.1%–19.0% homeless at time of admission. Finally, 15.1% in the training sample and 8.3% in the holdout sample had a high risk of suicide flag on their medical records in the month prior to the time of their hospitalization. In comparing these distributions to those of all patients making VHA visits for any reason over the same time periods weighted by number of visits, the stratification variables most strongly associated with psychiatric hospitalization were ages 20–55 (55.4%–57.7% versus 26.7%–27.6%), never married (23.0%–23.3% versus 12.4%–12.6%), separated-divorced (47.3%–61.7% versus 31.5%–31.8%), post-Vietnam era (22.9%–25.1% versus 12.0%–13.4%), Persian Gulf War era (29.9%–36.3% versus 18.0%–20.5%), currently or recently homeless (35.6%–41.1% versus 8.7%–10.3%), and having a high risk flag (8.3%–15.1% versus 0.5%–0.8%).

### Stratification Variable Models

We estimated initial multivariate logistic regression models that used all the stratification variables to predict suicides over each of the five risk time horizons. Logistic regression coefficients were exponentiated to create odds-ratios (OR). As described below, these models were subsequently used as controls to screen each other potential predictor one at a time. The stratification variable models were globally significant for each time horizon (χ^2^_35_^=^ 59.8–535.6, p < .001). Only three variables were significant in the 1-week model: race/ethnicity, with significant ORs of 4.2 for non-Hispanic Whites and 5.3 for “other” race/ethnicity (not non-Hispanic White, non-Hispanic Black, and other Hispanic) compared to non-Hispanic Blacks; religion, with significantly reduced ORs of 0.00–0.17 for Black and other Baptists and 0.12 for “other” Christians (not Roman Catholic) compared to other Protestants; and having a high risk suicide flag on the medical record prior to the time of hospitalization (OR=2.5) (**Supplementary Table 12**). Most of these predictors remained significant in models for longer time horizons (OR=2.4–3.0 for non-Hispanic White; OR=3.4–4.5 for “other” race/ethnicity; OR=0.26–0.5 for Baptists, OR=1.8–2.6 high risk flag), a pattern also found for most of the predictors that became significant only in models with longer time horizons. But the OR for “other” Christians was no longer significant over longer time horizons (OR=0.7–0.8) and the ORs for Roman Catholics, non-Christians, and veterans with no religion became significant (OR=0.5–0.6 for Roman Catholics in the 3- through 12-month models; OR=0.5–0.6 for non-Christians in the 3-month and 12-month models; OR=0.8–0.7 for no religion in the 6-month and 12-month models).

Four additional variables became significant in the 1-month model: male gender (OR=3.3, decreasing from 2.2 to 1.3 over longer time horizons); age (OR=0.4–0.5 for the two youngest quintiles, increasing to 0.6–0.8 over longer time horizons; and nonsignificant OR=0.6–0.9 for the two oldest quintiles, decreasing to significant OR=0.7 in the 12-month model); marital status (OR=2.1 for never married increasing to OR=2.5–2.9 in the 6- and 12-month models; and nonsignificant OR=1.1–1.3 for currently married in the 1- and 3-month models increasing to significant OR=1.6–1.7 in the 6- and 12-month models compared to currently separated); most recent era of active duty service (OR=2.6 for Persian Gulf War era in the 1-month model and OR=2.0–2.3 in models for longer time horizons; a nonsignificant OR=1.0 for the Pre-Vietnam era that became significant OR=1.9 in the 12-month model; and a nonsignificant OR=0.9 for the Vietnam era that became significant ORs=1.5–1.8 in the 6- and 12-month models compared to the post-Vietnam era). Homelessness became significant in the 3-month model (OR=0.7 for currently homeless remaining significant OR=0.6–0.07 in the 6- and 12-month models; OR=0.7 for recently homeless remaining significant in the 6-month model OR=0.7 but not in the 12-month model OR=0.9). Census Region and patient income became significant in the 6-month model (OR=1.8–1.6 for Midwest, OR=1.7–1.6 for South, and OR=1.4–1.4 for West in the 6- and 12-month models compared to the Northeast; OR=1.5 in the 12-month model for no income, OR=1.5–1.4 in the 6- and 12-month models for low income, and OR=1.3–1.7 for high-average and high incomes in the 6- and 12-month models compared to low-average income).

### Super Learner Results

#### Feature Selection

As noted above in the *Analysis Methods* section, we used three different feature selection methods to prune the more than 89,000 potential predictors included in the dataset. This process resulted in the selection of 1,221 features for the 1-week model, 2,411 for the 1-month model, 4,074 for the 3-month model, 5,675 for the 6-month model, and 8,071 for the 12-month model.

#### Classifier Weighting

As noted above in the *Analysis Methods* section, SL generates a cross-validated weight that defines the relative importance of the different classifiers in the ensemble. Neural network was the best classifier for the 1-week model and random forest was best for the other models and second most important for the 1-week model (**Supplementary Table 13**). Extreme gradient boosting was one of the top 5 classifiers in all models, support vector machines (with varying kernels) in 4 of the 5, generalized linear models in 4 of the 5, Bayesian additive regression trees in 1 of the 5, and elastic net in 1 of the 5.

#### Area Under the Receiver Operating Characteristic Curve

The AUCs of the five SL models when applied to the holdout sample were in the range .67–.74. Models for the shortest time horizons had the lowest AUCs (.67 for 1 week, .68 for 1 month). Models for the longer time horizons had higher AUCs (.73 for 3 months, .74 for 6 months, .74 for 12 months). These results are shown in the diagonals entries in **Table 2**. But a comparison of the performance of each model predicting suicides over each time horizon (i.e., comparing all entries in a single column of **Table 2**) found an unexpected result: that the model built to predict suicides over the 12-month time horizon also outperformed all other models predicting suicides over each shorter time horizon. AUC = .79 versus .67–.77 to predict suicides within the first week of hospital discharge, AUC = .82 versus .63–.77 to predict suicides within the first month of discharge, AUC = .78 versus .61–.73 to predict suicides within 3 months of discharge, AUC = .80 versus .61–.74 to predict suicides within 6 months of discharge, and AUC = .74 versus .60–.71 to predict suicides within 12 months of discharge. With the exception of a single inversion in predicting suicides within the first week of discharge (between the SL models designed to predict suicides within 3 and 6 months after discharge), a consistently monotonic association was found within each time horizon for AUC to increase as the time horizon for model development increased. Based on this result, we focused subsequent analyses on the SL model developed to predict suicides within 12 months of discharge. Consistent with the guarantee that SL outperforms the best classifiers in the ensemble, the AUCs of this best SL model averaged 0.02 higher than the best individual classifier in the ensemble (random forest) across all time horizons (**Supplementary Figure 1**).

**Table 2:** Area under the receiver operating characteristic curve (AUC) of the super learner model’s developer in the training sample for each time horizon to predict suicides in the holdout sample over each of the five time horizons.

	Time horizon for prediction in the holdout sample				
	1 week	1 month	3 months	6 months	12 months
**Time horizon for model development**					
1-week	.67	.63	.61	.62	.60
1-month	.71	.68	.70	.70	.67
3-month	.77	.76	.73	.72	.69
6-month	.75	.77	.73	.74	.71
12-month	.79	.82	.78	.80	.74

#### Operating Characteristics

**Figure 2:**
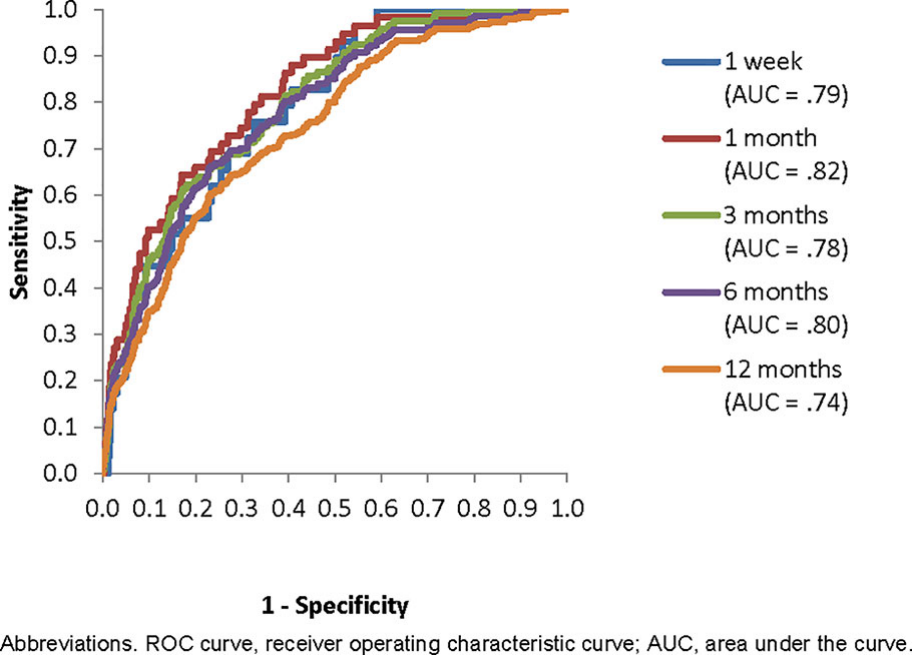
Receiver operating characteristic (ROC) curve for the best Super Learner model (to predict suicides within 12 months of hospital discharge) applied in the holdout sample to predict suicides over each of the five time horizons.

Inspection of the ROC curves for the best-fitting SL model (i.e., the model developed to predict suicides over the 12-month time horizon) in the holdout sample showed that the slope was steepest for 1-specificity in the range 0–0.05, which corresponds roughly to the 5% of patients with highest predicted suicide risk in the model (**Figure 2**). The sensitivities at this threshold show that these patients accounted for 24.1% of all suicides in the holdout sample that occurred in the 1 week after hospital discharge, 32.2% in 1 month, 26.9% in 3 months, 26.4% in 6 months, and 22.4% in 12 months (**Table 3**). This means that an intensive postdischarge case management program that was delivered only to the 5% of hospitalized patients with highest predicted suicide risk would capture 22.4%–32.2% of the patients who would otherwise die by suicide by one or more of the time horizons. Other consistent inflection points in the slope were at 1-specificity of about 0.2 and 0.6. A case management program delivered to the 20% or 60% of patients with highest predicted risk would capture 55.2%–66.1% (20% decision threshold) and 90.3%–100% (60% decision rule) of the patients who would otherwise die by suicide at one or more of the time horizons.

**Table 3:** Operating characteristics at a range of thresholds of the best Super Learner model (developed to predict suicides within 12 months of hospital discharge) applied in the holdout sample to predict suicides over each of the five time horizons.

Threshold P	SN		SP		PPV		Adjusted PPV	
	%	(SE)	%	(SE)	S/100k	(SE)	%	(SE)
**Suicides within 1 week of hospital discharge**								
.05	24.1	(0.1)	94.9	(0.1)	0.12	(0.01)	6.1	(0.5)
.10	44.8	(0.2)	89.9	(0.1)	0.11	(0.01)	5.7	(0.5)
.20	55.2	(0.2)	79.9	(0.1)	0.07	(0.01)	3.5	(0.4)
.60	100.0	(0.0)	39.9	(0.1)	0.04	(0.01)	2.1	(0.3)
**Suicides within 1 month of hospital discharge**								
.05	32.2	(0.1)	94.9	(0.1)	0.3	(0.0)	3.8	(0.2)
.10	52.5	(0.2)	89.9	(0.1)	0.3	(0.0)	3.1	(0.2)
.20	66.1	(0.1)	79.9	(0.1)	0.2	(0.0)	2.0	(0.1)
.60	98.3	(0.0)	39.9	(0.1)	0.1	(0.0)	1.0	(0.1)
**Suicides within 3 months of hospital discharge**								
.05	26.9	(0.1)	94.9	(0.1)	0.5	(0.0)	2.1	(0.1)
.10	46.2	(0.2)	89.9	(0.1)	0.5	(0.0)	1.9.	(0.1)
.20	63.0	(0.1)	79.9	(0.1)	0.3	(0.0)	1.3	(0.1)
.60	95.8	(0.1)	39.9	(0.1)	0.2	(0.0)	0.6	(0.0)
**Suicides within 6 months of hospital discharge**								
.05	26.4	(0.1)	94.9	(0.1)	0.9	(0.0)	1.8	(0.1)
.10	40.4	(0.1)	89.9	(0.1)	0.7	(0.0)	1.4	(0.0)
.20	61.5	(0.1)	79.9	(0.1)	0.5	(0.0)	1.1	(0.0)
.60	93.8	(0.1)	39.9	(0.1)	0.3	(0.0)	0.6	(0.0)
**Suicides within 12 months of hospital discharge**								
.05	22.4	(0.1)	94.9	(0.1)	1.2	(0.0)	1.2	(0.0)
.10	35.0	(0.1)	90.0	(0.1)	1.0	(0.0)	1.0	(0.0)
.20	55.3	(0.2)	80.0	(0.1)	0.8	(0.0)	0.8	(0.0)
.60	90.3	(0.1)	40.0	(0.1)	0.4	(0.0)	0.4	(0.0)

Threshold P, the proportion of patients classified at above the clinical threshold based on their predicted probabilities of suicide; SN, Sensitivity; SP, specificity; PPV, positive predictive value stated in terms of suicides expected per 100 patients over the time horizon; S, suicides; Adjusted PPV, PPV adjusted for the length of the time horizon to reflect the expected ratio of suicides to number of person-years of intervention over that time horizon; SE, standard error.

The proportion of patients receiving the intervention who would otherwise go on to die by suicide (i.e., PPV) is an important consideration in determining the potential value of any targeted suicide prevention intervention. As noted above in the section on analysis methods, PPV increases as the number of patients above the decision threshold decreases and as the time horizon increases. The highest PPV for our model is 1.2% for the .05 threshold over a 12-month time horizon. In other words, this is the proportion of patients above that threshold who would be expected to die by suicide in the 12 months after hospital discharge in the absence of any interventions beyond those currently provided by VHA. PPV decreases to 0.4% at the most liberal threshold considered (.60) over the same time horizon. By far the lowest PPVs are for the 1-week time horizon, where values are in the range 0.12%–0.04% across thresholds. That is, we would expect 0.12%–0.04% of patients above the .60 and .05 thresholds, respectively, to die by suicide in the first week after hospital discharge.

The adjusted PPVs, in comparison, decrease rather than increase as time horizons increase. The highest adjusted PPV is 6.1% for the .05 threshold over a 1-week time horizon. In other words, we would expect 6.1 deaths per year during the first week after hospital discharge for every 100 patients discharged per week over that time period (i.e., a total of 5,200 patients, each considered to be at risk for only the first week after discharge). This comparatively high adjusted PPV speaks to the potential value of special interventions focused on the high rates of imminent risk among patients shortly after discharge. Although actual benefit will depend on effectiveness, we see here that the potential benefit for a fixed level of clinical effort of longer preventive interventions with the small proportion of patients at high risk is greater than the benefit of shorter interventions for larger proportions of patients. For example, if the alternative interventions were equally effective and limited to the time horizons considered, our results suggest that more lives would be saved for a fixed level of effort by intervening with the 10% of patients at highest risk for 6 months (Adj PPV = 1.4%) than with the 20% of patients at highest risk for 3 months (Adj PPV = 1.3%) or the 60% of patients at highest risk for 1 month (Adj PPV = 1.0%).

In considering optimal allocation of intervention resources in this way, it is important to note that patients with high suicide risk also have significantly elevated risk of other negative outcomes, such as other types of death by external cause, suicide attempts, severe and permanently disabling injuries, and repeat psychiatric hospitalizations (20, 88). These other outcomes might be reduced by intensive postdischarge interventions designed to reduce suicide death. A formal analysis of intervention net benefit would be needed to take these added benefits into consideration. This would require us to make estimates of intervention effects among patients at different levels of risk across interventions that vary in duration. An added complication is that intervention effectiveness might vary depending on patient severity related to differential suicide risk. We discuss the logic of such an analysis below in the subsection on *Criticisms of Suicide Prediction Models* as part of the description of the concept of intervention net benefit.

#### Predictor Importance

The top 10 predictors accounted for 43.7% of overall model performance based on the gain metric in the Extreme Gradient Boosting algorithm (77), the top 25 predictors for 58.1%, the top 50 predictors for 70.3%, and the top 100 predictors for 81.4% of model performance. 3,521 predictors were needed to account for 100% of model performance. Sorting these predictors into categories shows that psychopathological risk factors were most important (accounting for 51.1% of overall model performance) followed by social determinants of health (26.2% of overall model performance, including 11.8% for socio-demographics, 2.9% for V codes, and 11.5% for small area geocode data) and history of suicidal behaviors (14.8%) (**Table 4**). The other categories of predictors were much less important (physical disorders 6.6%; medications classified by the US Food and Drug Administration as potential risk factors for suicide 0.7%).

**Table 4:** Predictor variable importance^1^ overall by category and for the predictors in the top 10, 11–25, and 26–50 in the best Super Learner model (to predict suicides within 12 months of hospital discharge)^2^.

**I. History of prior suicidal behaviors: 14.8% total importance**
4 in the top 10: 1Y suicide attempt (D); intake suicide attempt (D); 30D and 2Y suicide attempt (D) 2 in the top 11–25: 3Y suicide attempt (D); High risk flag in month prior to admission (D)
**II. Psychopathological risk factors: 51.1% total importance**
3 in the top 10: LT psychiatric hospitalizations (C); LT outpx cocaine dependence (D); LT outpx drug dependence (C) 8 in the top 11–25: LT outpx neurotic, personality, and other nonpsychotic disorders (C); LT inpx cocaine dependence (D); 1Y nonopioid analgesic medication (C); LT outpx unspecified schizophrenia (C); LT inpx cocaine dependence (Q); LT outpx unspecified schizophrenia (D); LT ED neurotic, personality and other nonpsychotic disorders (C); Current mood disorder (D) 9 in the top 26–50: LT outpx specialty MH neurotic, personality, and other nonpsychotic disorders (C); 90D sedative/hypnotic medication (C); 90D outpx recurrent MDD (C); Current episodic mood disorder (D); 3Y outpx nondependent drug abuse (C); LT outpx specialty MH drug dependence (C); LT PCP outpx alcohol dependence (C); LT inpx neurotic, personality, and other nonpsychotic disorders (C); 2Y outpx recurrent MDD (C)
**III. Quality of care:** Inadequately assessed
**IV. Time since hospital discharge:** Not included in the model^1^
**V-VII. Social determinants of health: 26.2%**
**V. Socio-demographics: 11.8% total importance**
2 in the top 10: Non-Hispanic Black (D); Age (C) 2 in the top 26–50: Sex (D); Non-Hispanic White (D)
**VI. ICD-9-CM E and V codes: 2.9% total importance**
1 in the top 11–25: 2Y housing problem (C) 2 in the top 26–50: Current other psychological or physical stress (D); 2Y multiple housing problems (C)
**VII. Small-area geocode data: 11.5% total importance**
1 in the top 10: BG % Non-Hispanic White x Px Non-Hispanic White (C) 3 in the top 11–25: County social capital (% voted in presidential election) (C); BG % Non-Hispanic Black x Px Non-Hispanic Black (C); County bankruptcy rate change, past 2Y vs. 3–5Y (C) 8 in the top 26–50: BG % Non-Hispanic Black (C); BG % Hispanic (C); County social capital (composite) (C); County social capital (charity rate) (C); County suicide rate past 3Y (C); County violent crime rate past 1Y (C); County unemployment rate past 1Y (C); County debt-to-income ratio change past 2Y vs. 3–5Y (C)
**VIII. Physical disorders: 6.6% total importance**
1 in the top 11–25: LT pain diagnosis (D) 4 in the top 26–50: 1Y vitamin medications (C); LT heart disease (D); LT high-risk physical disorders (C)^3^; 3Y chronic obstructive pulmonary disease (D)
**IX. Medications classified by FDA as increased risk of suicide: 0.7% total importance**
0 in the top 50
**X. Medical procedures: 0.2% total importance**
0 in the top 50

ICD-9-CM, International Classification of Diseases, Ninth Revision, Clinical Modification; FDA, US Food and Drug Administration.

^1^Variable importance was defined in terms of the Extreme Gradient Boosting (XGBoost) “gain” measure (77). We did not consider time since hospital discharge, which was category (iv) in our list of predictor categories, because the model was designed to predict suicide at any time in the 12 months after hospital discharge.

^2^**Geocode:** BG = The small-area geocode variable was defined over the Block Group of the patient’s residence; County = The small-area geocode variable was defined over the County of the patient’s residence; **Variable metric:** D = A yes-no dichotomous variable; C = A continuous count variable; Q = A truncated continuous variable transformed to quintiles of the count distribution; **Time:** 30D = The predictor was assessed over the 30 days before hospital admission; 1Y = The predictor was assessed over the 1 year (365 days) before hospital admission; 2Y = The predictor was assessed over the 2 years (730 days) before hospital admission; 3Y = The predictor was assessed over the 3 years (1,095 days) before hospital admission; LT = The predictor was assessed over the lifetime of the patient’s contact with the VHA system beginning January 1, 2000; **Treatment sector:** Intake = At the time of the focal hospitalization; ED = Emergency department; Inpx = Psychiatric inpatient; Outpx = Any outpatient treatment; Specialty outpx = Outpatient treatment by a mental health treatment provider; PCP outpx = Outpatient treatment by anyone other than a mental health treatment provider; No mention of treatment sector = Aggregation of the diagnosis or treatment across all sectors.

^3^High-risk physical disorders were defined based on Ahmedani et al. (32) as asthma, back pain, cancer (esophageal; head and neck; Hodgkin lymphoma; lung; mesothelioma; pancreatic; prostate; stomach; testicular), congestive heart failure, chronic obstructive pulmonary disease, diabetes mellitus, epilepsy, fibromyalgia, heart disease, HIV/AIDS, hypertension, migraine, multiple sclerosis, osteoporosis, Parkinson’s disease, psychogenic pain, renal disease, sleep disorders, and traumatic brain injury.

## Discussion

### Comparisons With Other Suicide Prediction Models

Most prior efforts to develop suicide prediction tools focused on one of three partially overlapping high-risk patient populations—patients in emergency departments with suicidal intent or after a suicide attempt, psychiatric inpatients during hospitalization, and psychiatric inpatients after discharge. These models are designed for use either at intake or discharge to help guide treatment planning. Meta-analyses suggest that the suicide rate among emergency department patients presenting with suicide intent or after a suicide attempt is about 1,600/100,000 within 1 year of the emergency department visit (89), that the suicide rate among inpatients is about 150/100,000 inpatient-years (90), and that the suicide rate after psychiatric hospital discharge is between 3,000/100,000 person-years in the first week after discharge and 650/100,000 person-years 4–12 months after discharge (49, 91). Although only about 2% of the US population in a given year either visit an emergency department with suicide intent, visit an emergency department after a suicide attempt, or are hospitalized for a psychiatric problem, such individuals account for nearly one-third of all US suicides (92).

As reviewed elsewhere, ML methods with a single classifier were used in most recent studies aimed at building suicide prediction models in these high-risk patient populations using predictors that included information collected from patient self-report scales, clinical rating scales, and administrative data (71). Prediction accuracy in our model was generally comparable to that in these earlier models even though we did not use any patient self-report data or clinician ratings data in building our model. Sensitivity was typically 0.6–0.7 in these earlier models when specificity was set at 0.8 (compared to sensitivity of 0.55–0.66 in our model) and 0.4–0.5 when specificity was set at 0.9 (compared to sensitivity of 0.35–0.52 in our model). In other words, the performance of our model is roughly comparable to that of previous ML models designed to predict suicide in high-risk patient samples, although the vast majority of previous studies focused on samples of emergency department patients or patients who made suicide attempts rather than on hospitalized patients. As noted below, we anticipate that ongoing refinements will improve the prediction accuracy of our model, perhaps substantially, but it is likely that an optimal version of such a model will have operating characteristics not dramatically higher than those found here unless a breakthrough occurs in the discovery of a critical biomarker.

In terms of predictors, our finding that psychopathological risk factors and prior suicidality were important is not surprising. It is somewhat surprising, though, that bipolar disorder did not figure more prominently than it did among the psychopathological risk factors. It is noteworthy in this regard that bipolar disorder was a powerful predictor when considered alone in the initial screening models, but other predictors more efficiently captured the variance due to bipolar disorder in the SL analysis. In particular, patients with bipolar disorder had a stronger history of suicidality and more risk factors involving social determinants of health than other patients. It is important to recognize in this regard, though, that predictor importance was defined in terms of prediction and not intervention. This means that interventions focused on improving bipolar disorder treatment among high-risk patients might very well be useful in reducing suicides even though a diagnosis of bipolar disorder was not one of the most important variables in making up the composite risk index.

Another surprising finding was that social determinants of health were quite important in making up the index. This might mean that we need to think in terms of upstream interventions to address the suicide problem, a possibility of increasing interest in many areas of medicine (93–95). However, as an opposite side of the coin in the above discussion of bipolar disorder, the fact that social determinants of health indicators were important *predictors* does not necessarily mean that they would be useful intervention targets, as they might be risk markers rather than causal risk factors (96). The association of homelessness with reduced probability of suicide in the model predicting SL scores is a case in point. The gross association of homelessness with suicide is positive, but the OR for homelessness in the multivariate model suggests that homelessness is protective. This is due to the fact that the high suicide rate among homeless patients is considerably lower than expected based on the fact that these patients experience a wide array of other risk factors for suicide that are assessed in the model. This kind of subadditive multivariate interaction should not be taken as evidence that homelessness is somehow protective against suicide, but as an indication that homelessness is a strong marker of the existence of this subadditive interaction.

A final surprising result involved our finding that the model developed for the 12-month time horizon outperformed the models developed for shorter time horizons in predicting suicides across those shorter time horizons. This is an important finding given that most prior ML models to predict suicides from administrative data used much shorter time horizons than 12 months. In particular, the VHA Reach Vet model (88), which is used to target preventive interventions to the roughly 35,000 VHA patients each year considered to be at highest suicide risk, is based on a 30-day time horizon, which we showed clearly to be suboptimal in the current application to inpatients. Our finding might reflect the fact that we had more statistical power to detect meaningful associations in the 12-month model because of the larger number of cases than in the models for shorter time horizons. This might explain why the number of predictors that passed our feature screening step increased substantially as the time horizon increased. Another possibility is that suicides became more predictable after the early weeks and months after hospital discharge, but this possibility is inconsistent with the finding in an ancillary analysis not reported here that SL models estimated for *conditional* suicide risk between the 1^st^ week and 1^st^ month after hospital discharge and between 2 and 3, 4 and 6, and 7 and 12 months after discharge did not vary substantially in their AUCs. Based on this result, it is possible that models designed specifically to predict imminent suicide risk in the first week after discharge or for longer time horizons less than 12 months (e.g., 1, 3, or 6 months) might improve on the model developed to predict 12-month suicides if a sufficiently large sample was available for training. Larger samples are available for other segments of the VHA population (e.g., outpatients with common mental disorders who report suicidality). As a result, the issue of the optimal time horizon for model development needs to be revisited anew each time a new population segment is targeted for model building.

Another observation related to model optimality involves the fact that we developed our model specifically for a particular segment of the patient population (i.e., psychiatric inpatients) at a particular time when clinical decision-making occurs (i.e., at time of discharge, when a discharge plan needs to be formulated). Other recently developed suicide risk ML models have this same characteristic, such as a model for suicide risk in the months after an outpatient primary care visit designed to provide decision support for clinicians in making specialty referrals [e.g., (97)]. It is possible that models of this sort perform better than models based on total populations of health plans [e.g., (88, 98)]. An interesting point of comparison is the VHA Reach Vet model, which was developed by analyzing data available from all veterans seen in the VHA system regardless of whether they carried diagnoses of or received treatment for mental disorders (88). The Reach Vet model attempted to isolate the 0.1% of all VHA users with highest suicide risk. These veterans were found to account for approximately 2% of all suicide deaths in VHA. Our model, in comparison, focuses on the roughly 1% of VHA patients who are hospitalized for a psychiatric disorder in a year. We found that 10% of this 1% of patients (i.e., the same 0.1% of all VHA patients defined as being above the risk threshold in the Reach Vet model) account for 35% of all the suicides that occur among inpatients in the 12 months after discharge. As noted in the introduction, psychiatric inpatients account for 12% of all VHA suicides over the 12 months after discharge, which means that the 10% of these ex-inpatients with highest risk account for approximately 4% of all VHA suicides (i.e., 35% of 12%), which is roughly twice as high a proportion as in the Reach Vet model. This might reflect the more sophisticated modeling procedures or expanded set of predictors in our model compared to the Reach Vet model, but the targeted focus on one segment of the patient population might also have played a part. We plan to investigate this issue in ongoing analyses by developing parallel models for other segments of the VHA patient population and determining if these models improve on the performance of a model for the overall VHA population.

### Criticisms of Suicide Prediction Models

Many critics have argued that prediction models like the one we presented here are not strong enough to justify use for clinical decision-making even if the costs of generating, updating, and making model results available to clinicians for decision support are de minimus (99–102). Two reasons are typically given for this conclusion: first, that the low PPV of the models at the decision thresholds would mean that interventions focused on patients classified high-risk would “subject many patients, who will never die by suicide, to excessive intrusion or coercion” (103); and second, that the low sensitivity of the models at these thresholds would mean that only a minority of suicides occurred among patients classified high-risk. Clinicians unaware of this low sensitivity might draw “false reassurance” from negative predictions and deny needed treatment to patients who have a meaningful risk of suicide but are incorrectly classified low-risk (102). These criticisms have become institutionalized in clinical practice guidelines that advise clinicians against using structured suicide prediction models and instead recommend that clinicians implement “an integrated and comprehensive psychosocial assessment” (104) of needs and risks with all psychiatric inpatients, psychiatric emergency department patients, and other patients considered to be at elevated suicide risk (105, 106).

But are clinical evaluations any more accurate than structured assessments in predicting subsequent suicides and SRBs? The evidence suggests not. Statistical models have long been known to be superior to unstructured clinical judgments in predicting a wide range of clinical outcomes (107), although varying over settings and decisions (108). Consistent with this evidence, a meta-analysis of 13 studies examining risk factors for suicide within 12 months of psychiatric hospital discharge found that clinical judgments at discharge were not much stronger predictors of subsequent suicides than were several other social, historical, and clinical variables assessed by patient self-report or extracted from administrative databases (26). A more recent meta-analysis of seven studies found that clinical assessments were only weakly associated with subsequent suicides among patients after hospital treatment of SRBs (109). Based on this evidence, review authors conclude that clinicians should focus on need for services rather than on suicide risk in assessing suicidal patients. But this recommendation overlooks the fact that clinical decisions about need for services should be informed by perceived suicide risk. This fact is recognized in the strategy for suicide prevention advanced by the US National Action Alliance for Suicide Prevention (110) as well as in related guidelines for identifying risk and protective factors, assessing level of risk, and developing an intervention plan based on clinical judgments (111, 112). However, given the greater accuracy of prediction models than clinical judgments about patient suicide risk, it makes sense for clinicians to have access to the results of these models as input in developing intervention plans.

There is a third criticism that could be raised here involving our suggestion that a prediction model such as the one we present could be used to target a new kind of intensive postdischarge case management intervention. It is important to note that we have no way to know if such an intervention would be effective in reducing postdischarge suicides and, if so, if it would be cost-effective to do so compared to existing interventions. Our assumption at the onset was that this kind of intervention would be too expensive to provide to all patients being discharged from psychiatric hospitalizations and that any hope of it being practical would require targeting. But it needs to be said that the existence of a prediction model would not make it practical to implement the intervention if it was not cost-effective relative to other uses of the equivalent clinical resources.

Assuming that such an intervention could be cost-effective if it was targeted correctly, where should the decision threshold be set using a model of the sort we developed? No agreement has emerged on this question (113). The thresholds we considered were based on observed inflection points in the ROC curves, but PPV will inevitably be very low for a rare outcome such as suicide for any decision threshold that includes more than a very small proportion of patients. And this reintroduces the criticism that prediction models with low PPV are not clinically useful. However, this argument is incorrect. As discussed in more detail elsewhere (70), Net Benefit (NB), not PPV, should be considered the key operating characteristic in evaluating the value of new interventions. NB is the standardized difference between the number of true positives at or above a decision threshold and the discounted number of false positives at or above that threshold, where the discount rate explicitly evaluates the value of intervening with a true positive (i.e., someone who would die by suicide in the absence of intervention) relative to the costs (both direct and indirect) of intervening with a false positive. Once the cost-benefit ratio is determined, an optimal decision threshold can be calculated, noting that the optimal decision threshold might be 0; that is, it is shown empirically that the intervention is not cost-effective for any patient. It is important to note that NB can be positive even when PPV is low if the costs of intervening with false positives are low relative to the benefits of intervening with true positives. That is why it is considered cost-effective to prescribe statins to adults aged 40–75 with mildly elevated total cholesterol even though annual PPV is only .0075 (which is lower than the PPVs rejected by critics of suicide prediction models as too low for targeting interventions) and statin treatment requires nearly 500 person-years of treatment to prevent one case of atherosclerotic cardiovascular disease (114).

### Future Directions

#### Improving Model Performance

We are continuing to make refinements by expanding the predictor set in several important ways. First, as noted in the introduction, recent UK research found suggestive evidence that a number of inpatient unit characteristics, such as staff turnover and average length of stay, were significant predictors of postdischarge suicide rates (14, 15). We are in the process of assembling a unit-level time series dataset for all the roughly 100 psychiatric inpatient units in VHA to assess these and other unit characteristics as potential indicators of treatment quality relevant to suicide risk. The same UK studies found that a number of policies for treating high-risk outpatients, such as the use of community outreach teams, were important predictors of geographic variation in suicide rates. We are assembling a time series dataset with an expanded set of such indicators for each VHA outpatient clinic where VHA psychiatric inpatients are transferred after hospital discharge.

Second, we are trying to expand the indicators of social determinants of health by using natural language processing (NLP) methods to elicit additional information from clinical notes. A growing number of methodological studies have shown that NLP can be used to generate such measures from clinical notes (115–118) and to elicit information about a wide range of social determinants of health relevant to suicide beyond the information captured in ICD-9 V codes (119–123).

Third, based on the unexpectedly strong influence of social determinants of health in our model, we are expanding the assessment of this domain in our next phase of model-building by adding the 450 variables in the LexisNexis Social Determinants of Health database to our predictor set (124). These variables assess various aspects of employment, finances, marital status, parenting status, and involvement with the criminal justice system for close to 300 million Americans and their neighborhoods. In addition to using these individual-level and neighborhood-level variables additively, we will also create more complex multivariate profiles to characterize mismatch between patients and their neighborhoods on a wide range of characteristics.

#### Precision Treatment

The criticism noted above that suicide prediction models have low sensitivity speaks to an important issue not addressed by these models: that the patients at highest suicide risk are not necessarily the patients most likely to be helped by existing interventions. As it happens, though, another class of models can be developed to help predict which available intervention is most likely to help a specific patient and the extent of that help (71). The estimates of predicted risk based on models of the sort presented in this paper can be used as input to such precision treatment models. Or the intervention could be limited to patients with meaningfully elevated predicted risk based on an initial model of this type. Or the predicted values from a model like the one developed here could be provided to clinicians as input to their clinical decision-making. But the critical distinction between the type of model developed in the current paper and precision treatment models is that the latter focus on interactions between patient characteristics and specific treatment alternatives with the goal of developing an individualized treatment rule (ITR) that predicts which treatment option is likely to be best for which patients (70, 125, 126). We plan to develop such a model as part of a pragmatic trial for intensive case management after psychiatric hospital discharge focused on the 60% of patients with meaningfully elevated risk of postdischarge suicide.

It is also possible to develop preliminary precision treatment models from the kinds of observational study designs used in the current report (70). However, this can be done only for intervention that is already being used in practice. If promising ITRs are developed in such studies, rigorous evaluation is needed in pragmatic trials. This is a much more feasible order of operations in some cases than beginning with a controlled trial sufficiently large to support the development of an ITR. As an example, we are involved in an investigation of this sort to study the circumstances under which patients should versus should not be hospitalized after nonfatal suicide attempts (SAs). As detailed elsewhere (71), it is unclear whether hospitalization (which occurs after 50% of VHA outpatient SAs) reduces subsequent SRBs (either suicide or subsequent SAs). A recent study carried out by UK investigators attempted to address this issue in a prospective observational study that used propensity score methods to adjust for baseline differences among acutely suicidal patients managed in four different ways (psychiatric hospitalization, general hospital admission as a psychiatric inpatient, psychiatric outpatient treatment, specialist evaluation without referral for treatment). Differences in subsequent 12-month suicidal behaviors across the four groups were largely nonsignificant after this risk adjustment (127, 128), implying either that whether a patient was hospitalized had no effect or that hospitalization was beneficial for some patients and harmful for a roughly equal number of patients. We are investigating the latter possibility.

Although theorizing exists about the patients most likely to be helped and those most likely to be hurt by hospitalization (129), little empirical research exists on these hypotheses (90). We are attempting to develop an ITR to provide guidance in making this decision in the immediate aftermath of an outpatient suicide attempt. It is infeasible to use controlled treatment trials to study the aggregate effects of these decisions given the large samples required (130) and rarity of SRBs other than among high-risk patients for whom randomization would be unethical. However, modern statistical methods applied to large electronic health record databases to adjust for (“balance”) baseline differences in patients across types of treatment can be used to estimate aggregate treatment effects (131, 132). Such methods often yield results similar to those in controlled treatment trials (133, 134). Extensions exist to develop ITRs using ML methods (135–137). This is, in fact, what we are attempting to do: to see if ensemble ML methods can be used to develop an ITR that allows us to estimate which specific patients should be hospitalized and which ones not after SAs, with the goal of reducing subsequent SRBs. If our observational analysis suggests that a useful ITR can be developed, we will implement a pragmatic trial to determine the validity of that conclusion. Such an ITR could have considerable value for clinical practice in providing empirical guidance in making this critical treatment decision.

#### Implementation Beyond VHA

Some of the key predictors in our model are unavailable in health systems other than the VHA, making it impossible to apply our model outside of VHA. The fact that most VHA patients are males is another unique characteristic of our sample. However, several innovations implemented here could be used in building models to predict suicides in other health systems. The most notable of these are expansions of the predictor set, investigation of diverse time horizons, and use of ensemble ML methods. With regard to the predictor set, we made more use than previous ML suicide studies of small-area geocode data and E-V codes to generate additional information about social determinants of health. We constructed composite condition indices that cut across the ICD hierarchy using information from external sources regarding such organizing constructs as pain severity and Food and Drug Administration drug warnings. And we used unsupervised analysis methods to develop multimorbidity profiles that we considered along with composites based on the ICD hierarchy. Extensive detail on predictor variable construction is provided in **Supplementary Tables**.

### Conclusions

We found that a model could be developed using only administrative data available while patients are still hospitalized to target inpatients with high risk of postdischarge suicide for intensive postdischarge case management. If an effective intervention of this or another sort could be provided to the 60% of patients classified by our model as having highest suicide risk prior to hospital discharge, we estimate that 90.3%–100% of the patients who would otherwise go on to die by suicide would be reached. If additional intervention could be provided only to the 20% of patients classified by our model as having highest suicide risk, we estimate that 55.3%–66.1% of the patients who would otherwise go on to die by suicide would be reached. It is important to note, though, that we provided no evidence to suggest that an additional intervention would be effective in this way. It might not be. This remains to be seen.

However, we noted at the onset that intensive postdischarge case management programs, which are not used in VHA, have been shown elsewhere to be effective in reducing suicides after psychiatric hospital discharge (9–16), leading to recommendations to add such programs to existing postdischarge suicide preventive interventions (17). The motivation for our model development exercise was the assumption that these labor-intensive programs could not be implemented cost-effectively given the rarity of postdischarge suicide unless they were targeted to recently discharged patients at high suicide risk. Our aim was to determine whether a prediction model could be developed that had a sufficiently high concentration of risk to make the consideration of targeting plausible. We have done that, showing, for example, that nearly one-third of all suicides occurring in the first month after hospital discharge occur among the 5% of patients classified by our model as having highest risk. Whether this is the optimal decision threshold for implementing a new postdischarge intervention or, indeed, if such an intervention would be cost-effective at any threshold in VHA is beyond the scope of this report. A cost-effectiveness analysis based on simulated data using information about the effects of existing interventions and our PPV estimates would be the next logical step in deciding whether the evidence is sufficiently strong to justify implementing a pragmatic trial [e.g., (138)].

## Data Availability Statement

The datasets generated for this study will not be made publicly available. The data used in this report were obtained from VHA clinical records based on a VA IRB-approved protocol. We are prohibited from sharing the data outside of those listed on the IRB-approved protocol. Requests to access these datasets should be directed to the corresponding author.

## Ethics Statement

The studies involving human participants were reviewed and approved by Research Ethics Committee of the VA Center of Excellence for Suicide Prevention and Harvard Medical School with a waiver of informed consent based on the fact that the data were deidentified. Written informed consent for participation was not required for this study in accordance with the national legislation and the institutional requirements.

## Author Contributions

RK, TB, SD, EK, MN, WP, LW, and RB conceptualized and designed the study. SG, HL, and NS organized the database. MB, SD, SG, JG, EK, JK, SL, WM, WP, NS, and JS contributed to the selection of predictors. HL and RB performed the statistical analysis with direct supervision from MP and NS. OD, AL, and PM worked with RK to develop the analysis plan. RK wrote the first draft of the manuscript. All authors contributed to manuscript revision, read and approved the submitted version.

## Funding

The research reported here was supported in part by the VISN 2 Center of Excellence for Suicide Prevention and the Precision Treatment of Mental Disorders Foundation. OD was additionally supported by 5K01HL135342 awarded by the National Heart, Lung, and Blood Institute of the National Institutes of Health and by grant 7IGMV33860009 from the American Heart Association.

## Conflict of Interest

In the past 3 years, RK received support for his epidemiological studies from Sanofi Aventis; was a consultant for Datastat, Inc, Sage Pharmaceuticals, and Takeda.

The remaining authors declare that the research was conducted in the absence of any commercial or financial relationships that could be construed as a potential conflict of interest
