# Supplementary material for: Using Administrative Data to Predict Suicide After Psychiatric Hospitalization in the Veterans Health Administration System
Source: Front Psychiatry. 2020 May 6;11:390. doi: 10.3389/fpsyt.2020.00390 (PMC7219514; doi:10.3389/fpsyt.2020.00390)
Supplement: Supplementary file 1 [file Table_1.docx]

| **Supplementary Table 1. Percent missing among VHA psychiatric hospitalizations, January 1, 2010 - December 31, 2013 (weighted n = 391,017.9)** | | | | | | |
| --- | --- | --- | --- | --- | --- | --- |
|  | | | | | | |
|  |  | **Total** |  | **Training** |  | **Validation** |
|  |  | **%** |  | **%** |  | **%** |
| Latitude/longitude |  | 4.52 |  | 4.78 |  | 3.89 |
| Race/ethnicity |  | 1.35 |  | 1.35 |  | 1.36 |
| Marital status |  | 0.35 |  | 0.38 |  | 0.30 |
| Religion |  | 0.03 |  | 0.03 |  | 0.03 |
|  |  |  |  |  |  |  |

| **Supplementary Table 2. Description of missing values** | |
| --- | --- |
|  |  |
| **Value** | **Description** |
| Latitude/longitude for all geographic variables | - Geographic variables were coded according to the year and month of each hospitalization. Variables were matched to hospitalizations using the patients' place of residence, which was represented by latitude-longitude coordinates that are updated on a quarterly basis by the VA. We matched these coordinates to a 12-digit FIPS code, which represents the state, county, census tract, and block group of a given location as determined by the 2010 Decennial Census to create all geographic variables. If the latitude/longitude for a visit was missing, we looked up the 5-digit FIPS code (which denotes only the state and county of the residence) that was recorded at the patient’s most recent visit prior to hospitalization. If the 5-digit FIPS code for this visit matched the FIPS code generated from that last latitude/longitude on file, we used the most recent non-missing latitude/longitude to assign the patient's 12-digit FIPS code for the present hospitalization. When the most recent 5-digit FIPS code did not match the 5-digit FIPS code from the last visit in which the patient had the coordinates of his/her residence recorded, we randomly assigned the patient visit to a block group in the county represented by the most recent 5-digit FIPS code. For random assignment, block groups were sampled with a probability proportional to the population size of the block group relative to the county represented by the patient’s most recent 5-digit FIPS code. If a geographic variable was missing for a specific block group or tract, we took the rate of that geographic variable from the next highest level of aggregation. |
|  |  |
| Sex | - Missings were rationally imputed as “male.” This was done prior to case-control selection. No one in the current sample had their sex imputed. |
|  |  |
| Age | - Date of birth came from the VHA Corporate Data Warehouse (CDW). If date of birth was missing in CDW, date of birth was taken from the National Death Index for those who died. Those still missing date of birth were excluded. |
|  |  |
| Race/ethnicity | - Race and ethnicity are recorded separately. Race/ethnicity was coded using the following hierarchy:   1. Hispanic: Endorsed Hispanic ethnicity   2. Non-Hispanic Black: Endorsed Black race and either did not endorse Hispanic ethnicity or ethnicity was missing   3. Non-Hispanic White: Endorsed White race and either did not endorse Hispanic ethnicity or ethnicity was missing   4. Missing: Missing race and ethnicity   5. Other: All others |
| Marital status, religion, urbanicity, census region, period of service | - All of these variables are recorded during outpatient encounters. The most recent value on or before the admission date was used. Religion and period of service also looked forward in time if they were missing prior to admission. Those still missing period of service were excluded. |
| Homelessness | - Homelessness was set to 0 if the associated ICD-9-CM codes, PTF codes (inpatient stays), or Stop Codes (outpatient services) were not present in the year prior to admission. |

| **Supplementary Table 2. Description of missing values** | |
| --- | --- |
|  |  |
| **Value** | **Description** |
|  |  |
|  |  |
| High risk flag | - High risk flag was set to 0 if it was not present in the patients’ records prior to admission. |
|  |  |
| Suicide attempts | - Suicide attempt was set to 0 if the associated ICD-9-CM codes or a SPAN report were not present prior to admission. |
| Driving times | - Driving times are available from 2009 and are updated at the end of each year. For those admitted before 2010, driving times were set to the times from 2009. For those admitted in 2010 or later, driving times were set to the times from the calendar year prior to admission. However, driving times for 2009 are not very complete, so those who were admitted in 2010 or earlier and were missing driving times had their driving times set to the times in 2010. For those still missing driving times, a random driving time with a matching ZIP code was selected. |
|  |  |
| Pain scale | - Pain scale was set to 0 if self-reported pain was missing prior to admission. |
|  |  |
| All others (diagnoses, E-codes, V-codes, medications, etc.) | - Values were set to 0 if it was not present prior to admission. |
|  |  |

| **Supplementary Table 3. History of prior suicidal behaviors** | | |
| --- | --- | --- |
|  |  |  |
| **Predictor** | **Identifier** | **Description** |
| **I. Suicide and self-inflicted injury** | ICD-9-CM codes: E950.X, E951.X, E952.X, E953.X, E954, E955.X, E956, E957.X, E958.X, E959; CCS code: 5.13 | A history of suicidal behavior is one of most significant predictors of suicide [1-3]. Suicidality was characterized with ICD-9-CM E codes, 1 ICD-9-CM V code, and 1 Clinical Classifications Software (CCS) code. We created a variable to indicate whether the patient was admitted for a suicide attempt, if they had a high risk flag at the time of admission [4, 5], and also yes/no indicators for each ICD-9-CM code. We also looked at 7 time periods before hospital admission (past 30 days, 90 days, 180 days, 365 days, 730 days, 1095 days, entire VHA history) and considered 5 treatment sectors: emergency department, psychiatric inpatient, any outpatient treatment, outpatient treatment by a mental health treatment provider, outpatient treatment by anyone other than a mental health treatment provider. We created the following at each time period/treatment sector: yes/no indicators for each ICD-9-CM code, counts of number of days with the diagnosis, 0-4 quintiles of the latter count, and number of suicide attempts. Also 4 indicators for whether there was a diagnosis of suicidal ideation in 3, 12, and 24 months before hospitalization (diagnosis in all 3 time periods, any combination of 2, only 1 time period, or none) [6-8]. |
| **II. Suicidal ideation** | V62.84 |  |
| **III. High risk** | VHA Patient Record Flags |  |
|  |  |  |

| **Supplementary Table 4.** **Psychopathological risk factors** | | |
| --- | --- | --- |
|  |  |  |
| **Predictor** | **Identifier** | **Description** |
| **I. International Classification of Diseases, Ninth Revision, Clinical Modification (ICD-9-CM)** |  |  |
| Organic psychotic conditions | 290.XX, 291.XX, 292.XX, 293.XX, 294.XX | There are 582 mental health ICD-9-CM diagnosis codes: 30 first level, 182 second level, and 370 third level. The codes are grouped into 4 diagnostic categories: 1) organic psychotic disorders, 2) other psychoses, 3) neurotic disorders, personality disorders, and other nonpsychotic mental disorders, and 4) intellectual disabilities. We looked at 7 time periods before hospitalization (past 30 days, 90 days, 180 days, 365 days, 730 days, 1095 days, entire VHA history) and considered 5 treatment sectors for each time period: emergency department, psychiatric inpatient, any outpatient treatment, outpatient treatment by a mental health treatment provider, outpatient treatment by anyone other than a mental health treatment provider. We created the following variables at each time period/treatment sector: yes/no indicators, continuous count of days, and quintiles of the count of days for each ICD-9-CM code and the 4 main diagnostic categories. Additionally, we created yes/no indicators for each diagnosis during current hospital admission [6, 9]. We also took a subset of ICD-9-CM physical illness codes that are park of mental illness in the Clinical Classifications Software (CCS). |
| Other psychoses | 295.XX, 296.XX, 297.X, 298.X, 299.XX |  |
| Neurotic, disorders, personality disorders, and other nonpsychotic mental disorders | 300.XX, 301.XX, 302.XX, 303.XX, 304.XX, 305.XX, 306.XX, 307.XX, 308.X, 309.XX, 310.XX, 311, 312.XX, 313.XX, 314.XX, 315.XX, 316 |  |
| Intellectual disabilities | 317, 318.X, 319 |  |
|  |  |  |
| **II. Multi-Level Clinical Classifications Software (CCS)** |  |  |
| Mental illness | 5, 5.1, 5.2, 5.3, 5.3.1, 5.3.2, 5.3.3, 5.4, 5.5, 5.5.1, 5.5.2, 5.5.3, 5.5.4, 5.5.5, 5.6, 5.6.1, 5.6.2, 5.6.3, 5.6.4, 5.7, 5.8, 5.8.1, 5.8.2, 5.9, 5.1, 5.11, 5.12, 5.13, 5.14, 5.14.1, 5.14.2, 5.15, 5.15.1, 5.15.2, 5.15.3, 5.15.4, 5.15.5, 5.15.6, 5.15.7, 5.15.8, 5.15.9 | The Clinical Classifications Software (CCS) is a hierarchical categorization system that groups ICD-9-CM diagnosis and procedure codes into more manageable categories. The CCS contains a single-level and multi-level classification system. There are 41 mental illness codes in the multi-level system: 1 first level, 15 second level, and 25 third level codes. We used the multi-level system to create the same set of variables as above [10]. |
|  |  |  |
| **III. Other disorders** |  |  |
| Sleep disorders | 291.82, 307.4X, 327.XX, 780.5X | There is evidence of a relationship between sleep difficulties and suicide. Owen-Smith et al. [11] found that those who died by suicide were 4 times as likely to have sleep disturbances. ICD-9-CM diagnosis codes were used to identify |

| **Supplementary Table 4 continued. Psychopathological risk factors** | | |
| --- | --- | --- |
|  |  |  |
| **Predictor** | **Identifier** | **Description** |
|  |  | sleep disorders and an indicator was created for having a sleep disorder at the 7 time periods before hospitalization (past 30 days, 90 days, 180 days, 365 days, 730 days, 1095 days, entire VHA history) [12]. |
| Fibromyalgia | 729.1 | Living with chronic pain is a major risk factor for suicide. The risk and frequency of suicide is particularly elevated in people who have fibromyalgia, psychogenic pain, and migraines [12-19]. We identified these conditions using ICD-9-CM diagnosis codes from Ahmedani et al. [12] and created an indicator for each condition at the 7 time periods before hospitalization. |
| Migraine | 346.XX |  |
| Psychogenic pain | 307.80, 307.89 |  |
|  |  |  |
| **IV. Polytrauma Clinical Triad (PCT)** |  |  |
| Posttraumatic stress disorder | 309.81 | The Polytrauma Clinical Triad, which is the co-occurrence of posttraumatic stress disorder, traumatic brain injury, and chronic pain, increases the risk for suicidal behavior and has been associated with suicide, particularly among Veterans [20-23]. The full triad or any combination of the PCT conditions can present (i.e., PTSD and chronic pain without TBI). We included depression in our triad as it is often comorbid with the PCT conditions and is a known predictor for suicide [22, 24]. We used ICD-9-CM codes from Pugh et al. [23] to identify each condition and created the following variables at the 7 time periods before hospitalization: a continuous count of the number of PCT conditions present, indicator for having all 4 PCT conditions, 3 out of 4, and 2 out of 4 PCT conditions. |
| Depression | 296.2X, 296.3X, 300.4, 311 |  |
| Traumatic brain injury | 310.2, 800.XX, 801.XX, 803.XX, 804.XX, 850.XX, 851.XX, 852.XX, 853.XX, 854.XX, 905.0, 907.0, 950.1, 950.2, 950.3, 959.01, 959.9, V15.52 |  |
| Chronic pain | 053, 307.81, 308.71, 337.2X, 338.0, 338.2X, 338.4, 350.X, 351.1, 352.1, 353.X, 355.XX, 356.X, 357.XX, 707.9, 710.5, 712.XX, 713.X, 714.XX, 715.XX, 716.XX, 720.XX, 721.XX, 722.XX, 723.0, 723.1, 723.3, 723.4, 724.0X, 724.1, 724.2, 724.3, 724.4, 724.5, 725, 729.1, 729.2, 729.5 |  |
|  |  |  |
| **V. Comorbid clusters** |  |  |
| Cluster 1: Polytrauma clinical triad + chronic diseases | PTSD: 309.81; Depression: 296.2X, 296.3X, 300.4, 311; TBI: 310.2, 800.XX, 801.XX, 803.XX, 804.XX, 850.XX, 851.XX, 852.XX, 853.XX, 854.XX, 905.0, 907.0, 950.1, 950.2, 950.3, 959.01, 959.9, V15.52; Chronic pain: 053, 307.81, 308.71, 337.2X, 338.0, 338.2X, 338.4, 350.X, 351.1, 352.1, 353.X, 355.XX, 356.X, 357.XX, 707.9, 710.5, 712.XX, 713.X, 714.XX, 715.XX, 716.XX, 720.XX, 721.XX, 722.XX, 723.0, 723.1, 723.3, 723.4, 724.0X, 724.1, 724.2, 724.3, 724.4, 724.5, 725, 729.1, 729.2, 729.5  Cardiac: 093.2X, 394.X, 395.X, 396.X, 397.X, 398.XX, 402.01, 402.11, 402.91, 404.01, 404.03, 404.11, 404.13, 404.91, 404.93, 410.XX, 411.XX, 412, 413.X, | Pugh et al. [23] identified 6 comorbid clusters of disorders in US Veterans Cluster from Afghanistan and Iraq: Cluster 1 = PCT + chronic disease; Cluster 2 = PCT; Cluster 3 = mental health + substance abuse; Cluster 4 = sleep, amputation, chronic disease; Cluster 5 = pain, moderate PTSD; Cluster 6 = relatively healthy. We included clusters 1 and 3 in our model to investigate their association with suicidality. We used ICD-9-CM codes from Pugh et al. [23] and defined each cluster as such: Cluster 1 = PCT (PTSD + depression + TBI + chronic pain) |

| **Supplementary Table 4 continued. Psychopathological risk factors** | | |
| --- | --- | --- |
|  |  |  |
| **Predictor** | **Identifier** | **Description** |
|  | 414.XX, 416.X, 417.9, 424.XX, 426.10, 426.11, 426.13, 426.2, 426.3, 426.4, 426.51, 426.52, 426.53, 426.6, 426.7, 426.8X, 427.0, 427.2, 427.31, 427.60, 427.9, 428.XX, 746.3, 746.4, 746.5, 746.6, 785.0, V42.1, V42.2, V43.3, V45.0X; Hypertension: 401.X, 402.10, 402.90, 404.10, 404.90, 405.XX, 437.2, 642.XX; Diabetes: 250.XX; Obesity: 278.0X; Osteoarthritis: 715.XX; Irritable bowel disease: 555.X, 556.X; Peripheral vascular disease: 440.XX, 441.2, 441.4, 441.7, 441.9, 443.1, 443.2X, 443.8X, 443.9, 447.1, 557.1, 557.9, V43.4; Cerebrovascular disease: 430, 431, 432.X, 433.XX, 434.XX, 435.X, 436, 437.X, 438.XX; Seizures: 345.XX; Cognitive impairment/dementia: 046.1X, 046.3, 290.XX, 294.XX, 331.XX; Other neurological conditions: 332.0, 333.4, 333.5, 334.X, 335.XX, 340, 341.XX, 345.0X, 345.1X, 345.4X, 345.5X, 345.8X, 345.9X, 348.1, 348.3X, 780.3X, 784.3; Rheumatoid arthritis/collagen disease: 701.0, 710.X, 714.XX, 720.XX, 725 | and at least one chronic disease (cardiac, hypertension, diabetes, obesity, osteoarthritis, irritable bowel disease, peripheral vascular disease, cerebrovascular disease, seizures, cognitive impairment/dementia, other neurological conditions, rheumatoid arthritis/collagen disease); Cluster 3 = at least 2 mental health disorders (PTSD, MDE, GAD, BPD) and substance use disorder. We did not include schizophrenia in cluster 3. We created yes/no indicators for each cluster at the 7 time periods before hospitalization (past 30 days, 90 days, 180 days, 365 days, 730 days, 1095 days, entire VHA history). |
| Cluster 3: Mental health disorders + substance abuse disorders | Posttraumatic Stress Disorder: 309.81; Depression: 296.2X, 296.3X, 300.4, 311; Anxiety: 300.0X, 300.2X, 300.3, 309.21; Bipolar Disorder: 296.0X, 296.1X, 296.4X, 296.5X, 296.6X, 296.7, 296.8X, 296.9X, 301.13  Alcohol-induced mental disorders: 291.XX; Drug-induced mental disorders: 292.XX; Alcohol dependence syndrome: 303.XX; Drug dependence syndrome: 304.XX; Nondependent abuse of drugs: 305.XX |  |
|  |  |  |
| **VII. Homicidal ideation** | V62.85 | Suicide and homicide are closely related [25]. We created 4 indicators for homicidal ideation before hospitalization: 1) Homicidal ideation at 3, 12, and 24 months 2) at 2 out of 3 time periods, 3) at 1 time period, 4) No homicidal ideation at any of the 3 time periods [6, 8]. We also created a yes/no indicator for homicidal ideation at time of hospitalization and an indicator and count of number of days at the 7 time periods before hospitalization for each of the 5 treatment sectors. |
|  |  |  |
| **VIII. ICD-9-CM V codes for mental and behavioral problems** |  |  |
| Personal history of mental disorder | V11.X | ICD-9-CM V codes provide details about circumstances, problems, or other factors that influence health status and health services [6]. We chose 16 V codes to characterize mental and behavioral problems: 2 first level, 12 second level, and 2 third level. We looked at 7 time periods before hospitalization (past 30 days, 90 days, 180 days, 365 days, 730 days, 1095 days, entire VHA history) and considered 5 treatment sectors for |

| **Supplementary Table 4 continued. Psychopathological risk factors** | | |
| --- | --- | --- |
|  |  |  |
| **Predictor** | **Identifier** | **Description** |
| Mental and behavioral problems | V40.XX | each time period: emergency department, psychiatric inpatient, any outpatient treatment, outpatient treatment by a mental health treatment provider, outpatient treatment by anyone other than a mental health treatment provider. We created yes/no indicators and continuous count of days for each of the 16 V codes. Additionally, we created yes/no indicators for each V code assigned during current hospital admission. |
|  |  |  |
| **X. Length of current hospitalization** | Number of days between admission and discharge | Studies have reported that length of hospital stay is a possible predictor for suicide, particularly psychiatric hospitalization [3, 26]. |
|  |  |  |
| **XI. Psychiatric hospitalization** | Count of previous psychiatric hospitalizations | Psychiatric hospitalization is associated with suicide, especially upon discharge [3, 27-29]. Forte et al. [30] conducted a review of 48 studies that looked at suicide and discharge from psychiatric hospitals and found that suicide post discharge was 23 times that of the general population. |
|  |  |  |
| **XII. Noncompliance with treatment** | V15.81, V62.6 | Troister et al. [3] and others found that noncompliance with treatment was a risk factor for suicide. We created 4 indicators for noncompliance with treatment before hospitalization using 2 ICD-9-CM V codes: 1) Noncompliance at 3, 12, and 24 months 2) Noncompliance at 2 out of 3 time periods, 3) Noncompliance at 1 time period, 4) No noncompliance at any time period [3, 6]. |
|  |  |  |
| **XIII. VA National Formulary Drug Classification: Central nervous system medications** |  |  |
| Opioid analgesics | CN101, CN102 | Psychotropic medications have many adverse side effects, including suicidal behaviors [31-34]. The VA National Formulary (VANF) is listing of drugs and supplies available at all VA facilities. As of October 2019, the VANF contained 29,290 individual pharmaceutical products, divided into 32 major drug classes, 287 minor drug classes, and 255 sub-classes [35]. The Central Nervous System Medications (CN000) in the VANF are divided into 12 minor classes and 21 sub-classes. We took the 3,087 psychotropic medications in our dataset and created |
| Non-opioid analgesics | CN103, CN104 |  |
| Other analgesics | CN100, IN500 |  |
| Antimigraine agents | CN105 |  |
| Anesthetics | CN200, CN201, CN202, CN203, CN204, CN205 |  |
| Sedatives/hypnotics | CN300, CN301, CN302, CN309 |  |
| Other sedatives/hypnotics | IN520, IN570 |  |
| Anticonvulsants | CN400 |  |

| **Supplementary Table 4 continued. Psychopathological risk factors** | | |
| --- | --- | --- |
|  |  |  |
| **Predictors** | **Identifier** | **Description** |
| Other anticonvulsants | IN530 | 19 sub-classes and 3 minor classes of CNS drugs (antidepressants), using the VANF minor/sub-classes from the CN000 and IN000 (only CNS drugs) group [36]. We assigned a code for each different kind of CNS drug listed in our dataset (e.g., code 1=DIAZEPAM 2 MG TAB; code 2=DIAZEPAM 5 MG TAB), which resulted in 560 different drugs prescribed to the Veterans in our sample. We then counted every different kind of drug prescribed to each Veteran in the 19 sub-classes and 3 minor classes in the past 90 and 365 days. |
| Antiparkinson agents | CN500 |  |
| Antivertigo agents | CN550 |  |
| Antidepressants |  |  |
| Tricyclic antidepressants | CN601 |  |
| Monamine oxidase inhibitor antidepressants | CN602 |  |
| Other antidepressants | CN600, CN609, IN550 |  |
| Antipsychotics | CN700, CN701, CN709 |  |
| Lithium salts | CN750 |  |
| Other antipsychotics | IN580 |  |
| Stimulants | CN800, CN801, CN802, CN809, CN850 |  |
| Other central nervous system medications | CN900, IN590 |  |
| Other antidotes/deterrents | IN595 |  |
| Central nervous system investigational drugs | IN500, IN520, IN530, IN550, IN570, IN580, IN590, IN595 |  |
|  |  |  |
| **XIV. Protective medications** |  |  |
| Antipsychotics | clozapine, olanzapine, quetiapine | There is a high prevalence of suicide among patients with psychotic disorders. Certain antipsychotic medications are known to be better for preventing suicide in schizophrenic psychosis. Based on the literature, we chose to include clozapine, olanzapine, and quetiapine in our model to determine if there are protective effects of these drugs. Including clozapine was an obvious decision, as noted on the FDA label: “Clozapine tablets are indicated for reducing the risk of recurrent suicidal behavior in patients with schizophrenia or schizoaffective disorder who are judged to be at chronic risk for re-experiencing suicidal behavior, based on history and recent clinical state. The effectiveness of clozapine tablets in reducing the risk of recurrent suicidal behavior was demonstrated over a two-year treatment period in the InterSePT trial” [37]. Additionally, a large multicentre RCT of schizophrenic patients at high risk for suicide found decreased risk of suicidal behavior in patients taking clozapine vs olanzapine [38]. Pompili et al. [39] reviewed the literature and |

| **Supplementary Table 4 continued. Psychopathological risk factors** | | |
| --- | --- | --- |
|  |  |  |
| **Predictor** | **Identifier** | **Description** |
|  |  | found that the top 3 antipsychotics with antisuicidal effects were clozapine, olanzapine, and quetiapine. We created a yes/no indicator for a diagnosis of psychotic disorder (ICD-9- CM codes 295.XX, 297.X, 298.X) who are prescribed clozapine, olanzapine, or quetiapine in the past 90 days and 365 days. |
| Long-acting injectable antipsychotics (LAIs) | aripiprazole, fluphenazine, haloperidol, olanzapine, paliperidone, risperidone | Long-acting injectable antipsychotics are intramuscular injectable antipsychotics that are long lasting, administered less frequently, and decrease risk factors (e.g., nonadherence) commonly associated with suicide in patients with psychotic disorders [40-44]. We created indicator for taking a long-acting injectable antipsychotic medication in past 90 days and 365 days and having any psychotic disorder (ICD-9-CM codes 295.XX, 297.X, 298.X). |
| Lithium | lithium | The rate of suicide and risk of suicidal behavior is also very high in patients with bipolar disorder. Lithium is considered the gold standard for suicide prevention in bipolar disorder. Over the years, multiple studies have found a significant reduction in suicide rate and depression among patients with severe, recurrent unipolar depression who are treated with lithium [45-48]. Lithium in public drinking water decreased suicide rate in Texas [49-51]. We created a yes/no indicator for a diagnosis of bipolar disorder (ICD-9-CM codes 296.0X, 296.1X, 296.4X, 296.5X, 296.6X, 296.7, 296.8X, 296.9X, 301.13) and prescribed lithium in the past 90 days and 365 days. |
| Medications to manage extrapyramidal side effects of antipsychotics | Medications: amantadine, atenolol, benztropine, biperiden, clonazepam, diphenhydramine, lorazepam, metoprolol, orphenadrine, procyclidine, propranolol, trihexyphenidyl; Antipsychotics: acetophenazine, aripiprazole, asenapine, brexpiprazole, cariprazine, chlorpromazine, chlorprothixene, clozapine, fluphenazine, haloperidol, iloperidone, loxapine, lurasidone, mesoridazine, methotrimeprazine, molindone, olanzapine, paliperidone, perphenazine, pimavanserin, piperacetazine, promazine, quetiapine, risperidone, thioridazine, thiothixene, trifluoperazine, triflupromazine, ziprasidone | Antipsychotic medications have a wide range of side effects, include problems such as akathisia, movement problems, parkinsonism. Some of these side effects carry risks, including suicide. These 12 medications can offset the deleterious side effects of antipsychotics that often contribute to suicidality [52-54]. We counted the number of prescribed medications to past 90 and 365 days and multiplied that by also using an antipsychotic medication to generate a 0-12 score. A score of 0-12 was assigned to each case |
|  |  |  |

| **Supplementary Table 5. Social determinants of health: Socio-demographics** | |
| --- | --- |
|  |  |
| **Predictor** | **Identifier** |
| **I. Age** | 20-40, 41-50, 51-55, 56-61, 62-98 |
|  |  |
| **II. Sex** | Male, female |
|  |  |
| **III. Census region** | Midwest, Northeast (including Puerto Rico and US Virgin Islands), South, West (including Pacific territories) |
|  |  |
| **IV. Marital status** | Married, divorced (including previously married & missing), never married, separated, widowed |
|  |  |
| **V. Income** | No income, low ($1-7,632), low-average ($7,633 - $15,264), high-average/high (>$15,264) |
|  |  |
| **VI. Period of service** | Persian Gulf War, Post-Vietnam, Pre-Vietnam, Vietnam era |
|  |  |
| **VII. Race/ethnicity** | Hispanic, Non-Hispanic Black, Non-Hispanic White, other |
|  |  |
| **VIII. Religion** | Baptist (Black Baptist, Evangelical and other Baptist), Other Protestant, Roman Catholic, Other Christian, Other non-Christian, none |
|  |  |
| **IX. Urbanicity** | Metro area with population greater than 1 million, 250,000 to 1 million, less than 250,000; Urban area with population greater than 19,000; Urban/rural area population with less than 20,000 |
|  |  |

| **Supplementary Table 6. Social determinants of health: ICD-9-CM E & V codes** | | |
| --- | --- | --- |
|  |  |  |
| **Predictor** | **Identifier** | **Description** |
| **I. Accidents** | E800.X, E801.X, E802.X, E803.X, E804.X, E805.X, E806.X, E807.X, E810.X, E811.X, E812.X, E813.X, E814.X, E815.X, E816.X, E817.X, E818.X, E819.X, E820.X, E821.X, E822.X, E823.X, E824.X, E825.X, E826.X, E827.X, E828.X, E829.X, E830.X, E831.X, E832.X, E833.X, E834.X, E835.X, E836.X, E837.X, E838.X, E840.X, E841.X, E842.X, E843.X, E844.X, E845.X, E846, E847, E848, E849.X, E850.X, E851, E852.X, E853.X, E854.X, E855.X, E856, E857, E858.X, E860.X, E861.X, E862.X, E863.X, E864.X, E865.X, E866.X, E867, E868.X, E869.X, E880.X, E881.X, E882, E883.X, E884.X, E885.X, E886.X, E887, E888.X, E890.X, E891.X, E892, E893.X, E894, E895, E896, E897, E898.X, E899, E900.X, E901.X, E902.X, E903, E904.X, E905.X, E906.X, E907, E908.X, E909.X, E910.X, E911, E912, E913.X, E914, E915, E916, E917.X, E918, E919.X, E920.X, E921.X, E922.X, E923.X, E924.X, E925.X, E926.X, E927.X, E928.X | ICD-9-CM E codes are used to describe external causes of injury and poisoning [6, 8]. There are 14 major categories of accidents: railway accidents; motor vehicle traffic accidents; motor vehicle nontraffic accidents; other road vehicle accidents; water transport accidents; air and space transport accidents; vehicle accidents, not elsewhere classifiable; accidental poisoning by drugs, medicinal substances, and biologicals; accidental poisoning by other solid and liquid substances, gases, and vapors; accidental falls; accidents caused by fire and fames; accidents due to natural and environmental factors; accidents caused by submersion, suffocation, and foreign bodies; other accidents. We counted the number of E codes present for each of the 14 different categories of accidents at 3 time periods: at the time of hospitalization, in the past 90 days, and the past 365 days. |
|  |  |  |
| **II. Psychosocial problems** |  |  |
| Problems with housing, material resources, and social isolation | V60.0, V60.1, V60.2, V60.3, V60.4, V60.89 | Adverse social determinants of health are strong predictors of suicidality and self-harm. ICD-9-CM V codes provide details about circumstances, problems, or other factors that influence health status and health services [6]. A subset of V codes related to psychosocial problems that are often associated with mental disorders and suicide were chosen [8]. We grouped these V codes into 5 categories: problems with housing, material resources, and social isolation; separation, divorce, or bereavement; counseling for marital problems; other psychosocial circumstances or stresses [55-60]. We created a count of the total number of V codes in the 3, 12, and 24 months before hospitalization. We also created 4 indicators for each of the 5 categories of psychosocial problems: 1) problem present in 3, 12, and 24 months 2) problem at 2 out of 3 time periods, 3) problem at 1 time period, 4) No problem at any time period [6]. |
| Separation, divorce, or bereavement | V61.03, V61.07, V62.82 |  |
| Counseling for marital problems | V61.1X |  |
| Unemployment | V62.0 |  |
| Other psychological or physical stress | V62.29, V62.3, V62.4, V62.5, V62.6, V62.81, V62.89, V62.9 |  |
| **III. Homelessness** | ICD-9-CM code: V60.0; Patient Treatment File (PTF) Inpatient Codes: 28, 37; Outpatient Stop Codes: 522, 528, 529, 530, 590 | Homelessness is a risk factor for suicide, especially among US Veterans [61]. We created an indicator variable for current homelessness, homeless in the past 12 months, and not homeless in the past 12 months. We defined homelessness as having the ICD-9-CM V code “lack of housing,” an inpatient code for “homeless stays,” or an outpatient stop code for “homeless services.” |
| **Supplementary Table 6. Social determinants of health: ICD-9-CM E & V codes** | | |
|  |  |  |
| **Predictor** | **Identifier** | **Description** |
| **IV. Abuse and assault** |  |  |
| Perpetrator of abuse | E967.X | Sexual assault is strongly associated with suicide [62]. ICD-9-CM E codes describe external causes of injury and poisoning [6]. We created a variable to indicate rape, the victim of assault, or the perpetrator of abuse at the time of hospitalization. We also look at the past 90 and 365 days and created indicators for rape and perpetrator of abuse along with a count of number of days as victim of assault. |
| Victim of assault | E960.0, E961, E962.X, E963, E964, E965.X, E966, E967.X, E968.X |  |
| Victim of rape | E960.1 |  |
|  |  |  |
| **VI. Other psychosocial circumstances & stresses** | V15.XX, V60.XX, V61.XX, V62.XX, V69.X | We chose a subset of V codes to characterize other psychosocial circumstances. We looked at 7 time periods before hospitalization (past 30 days, 90 days, 180 days, 365 days, 730 days, 1095 days, entire VHA history) and considered 5 treatment sectors for each time period: emergency department, psychiatric inpatient, any outpatient treatment, outpatient treatment by a mental health treatment provider, outpatient treatment by anyone other than a mental health treatment provider. We created yes/no indicators and continuous count of days for each of the V codes. Additionally, we created yes/no indicators for each V code assigned during current hospital admission [6]. |
|  |  |  |

| **Supplementary Table 7. Social determinants of health: Small area geocode data** | |
| --- | --- |
|  |  |
| **Predictor** | **Description** |
| **I. Alcohol outlets** | Researchers have investigated the contributions of both “on-premise” alcohol outlets (viz., bars and nightclubs) with “off-premise” alcohol outlets (viz., beer, wine, and liquor stores) in one’s neighborhood on suicide. On- and off-premise alcohol outlet density are associated with alcohol-related suicides at the county level and zip code level [63, 64]. We summed the number of on-premise and off-premise alcohol outlets in each block group using the Infogroup US Historical Business datasets [65]. We then computed the number of alcohol outlets per 100,000 people in each block group. Finally, we assigned to each patient visit the alcohol outlets rate from two years prior to the visit. |
|  |  |
| **II. Bankruptcy rate** | Bankruptcy is associated with suicidal behavior [66, 67] at the individual level. We are using the neighborhood level variable as a rough proxy for the individual level variable. In addition, there is a possibility that there is a contextual effect as a measure of neighborhood deprivation (see below). We downloaded the annual number of bankruptcy cases in each county from the U.S. Public Access to Court Electronic Records (2019) system [68]. We summed the number of bankruptcies filed by residents of a given county. We computed the number of total bankruptcy cases in each county per 100,000 people for each year using Census county population estimates [69]. We assigned to patient visits the bankruptcy rate in the patient’s county in the two most recently available years, as well as the interaction between the bankruptcy rate in the previous two years and the change in bankruptcy rate in the previous two years compared to the three years before that. |
|  |  |
| **III. Unemployment** | Geographic variation in unemployment is positively associated with geographic variation in suicide rates [70, 71]. One study estimates that suicide rates are the highest when a high proportion of labor force participants have remained without a job for 15-26 weeks [72]. We averaged the county-level unemployment rate from 3 and 4 months prior as a proxy for high suicide risk induced by remaining unemployed for 15+ weeks. We downloaded the county-level monthly unemployment rate from the Bureau of Labor Statistics [73]. We also downloaded the 5-year block group unemployment rate from the American Community Survey [74]. We also downloaded the 5-year block group unemployment rate from the American Community Survey. We used five variables in our final models, which together represent the absolute unemployment rate (1, 2), fluctuations in unemployment rate (3), and how severe the unemployment rate is in one’s immediate neighborhood relative to the broader area in which one might look for a job (4, 5). The following were included as features: (1) County-level unemployment averaged for 3 and 4 months ago; (2) County-level unemployment from 3-14 months ago; (3) The proportional change in unemployment in the past 3-4 months compared to the previous year (i.e., difference between (1) and (2), divided by (2)); (4) annual block group unemployment rate divided by annual county-level unemployment for that year; and (5) the interaction between (2) and (4). |
|  |  |
| **IV. Social capital** | Social capital refers to the properties of a social structure that facilitate cooperation for mutual benefit [75]. Suicide rates are inversely rated to social capital at both the state and county level in the U.S. [70, 76]. Because social capital manifests itself in individuals through participation in associational activities, Rupasingha et al. [77] chose the following four indicators as measures of social capital: Census Participation Rate; Number of Charitable Organizations; Presidential Voting Rate; and Association Density. In addition to coding these four variables individually, we also created an unweighted composite of the variables. |
| Census participation | We retrieved the 2000 and 2010 census tract participation rates from the [78]. We imputed the county-level rate for missing data at the tract level. |
| Charitable organizations | We obtained the number of tax-exempt non-profit organizations from the Urban Institute’s National Center for Charitable Statistics (2013) [79]. We excluded charities that have not filed for tax-exempt non-profit status in the past two years as well as charities with an international focus. |
| Presidential voting rate | The percentage of people in each county who voted was retrieved from Pennsylvania State University’s Northeast Regional Center for Rural Development, which provides the county-level social capital index for various years. They purchased the data from Dave Leip’s Atlas of U.S. Presidential Elections (<https://uselectionatlas.org/>). Patient visits were assigned the county-level rate from the most recent presidential election. |

| **Supplementary Table 7 continued. Social determinants of health: Small area geocode data** | |
| --- | --- |
|  |  |
| **Predictor** | **Description** |
| Association density | We summed the number of associations or organizations that promote social capital in each block group using the Infogroup US Historical Business datasets [65]. We then computed the number of associations per 100,000 people in each block group. Finally, we assigned to each patient visit the association rate from two years prior to the visit. We counted organizations included in Rupasingha et al.’s [77] measure of association density rate and supplemented this list with those included by Steelesmith et al. [80] in their index of social capital, which was inversely associated with suicide rates at the county level. |
|  |  |
| **V. Debt to income ratio** | Several studies indicate that high personal debt relative to assets in the previous 12 months predicts suicidal ideation, attempts, and completion at the individual level [81, 82]. We don’t have a measure of debt to income ratio at the individual level and are using the geographic variable as a rough proxy for individual variable. In addition, there may be a contextual effect of this variable as this could be used as a measure of neighborhood deprivation (see below). We measured debt using each county’s median household debt-to-income ratio. The Federal Reserve (2018) [83] computes quarterly median debt-to-income ratios based on debt information from the Federal Reserve Bank of New York Consumer Credit Panel/Equifax Data, and household income information from the Bureau of Labor Statistics. |
|  |  |
| **VI. Deprivation** | Researchers have identified many indicators of deprivation that are higher in geographic areas with elevated suicide rates, including income inequality, low educational attainment, poverty, unemployment, household crowding, and not owning a motor vehicle [71, 84-86]. We created a composite based on a version of the Area Deprivation Index [87] used by Steelesmith et al. [80], who found that it was positively associated with county-level suicide rates. However, Steelesmith et al. [80] coded the variables at the county level. In addition, we added a measure of blue-collar occupation prevalence, and removed a measure of owner-occupied housing because it was redundant with the measure of rented housing in the fragmentation measure. We had 22 total deprivation measures: median number of rooms per housing unit (block group), proportion of housing units with 4 or more bedrooms (block group), proportion of households with more than 1 person per room (“crowding”; block group), proportion of population aged 25+ with < 9 years of education (“no high school”; census tract), proportion of population aged 25+ with at least a high school diploma (census tract), proportion of labor force unemployed (block group), proportion of employed persons in white-collar (managerial, professional, art/science) occupations (census tract), median family income (census tract), proportion of population exceeding 150% of poverty threshold (block group), proportion of families below poverty line (block group), income Gini coefficient (block group), proportion of households with one parent of children aged under 18 (“single parent households”; census tract), median home value (census tract), median gross rent (census tract), median monthly mortgage (block group), median annual real estate taxes (block group), employed persons in unskilled blue-collar (i.e., service, construction, or transportation) occupations (census tract), proportion of households without a motor vehicle (census tract), proportion of households without a telephone (block group), proportion of occupied housing units without complete plumbing (census tract), proportion of households receiving public assistance income (block group), and proportion of households receiving supplemental security income (SSI; block group). We obtained all 22 measures from the American Community Survey [74]. After reverse-coding variables in which higher values reflected greater wealth, we summed these variables together to create a composite deprivation variable. |
|  |  |
| **VII. Firearm outlets** | Steelesmith et al. [80] found that the county rate of firearms dealers predicted county-level suicide rates. And households with guns have higher rates of suicide by gunshot but not by other methods [88]. We used the North American Industry Classification System (NAICS) codes identified in Steelesmith et al. [80] to sum the number of establishments selling firearms in each block group using the Infogroup US Historical Business datasets [65]. We then computed the number of firearms outlets per 100,000 people in each block group. Finally, we assigned to each patient visit the firearms outlets rate from two years prior to the visit. |
|  |  |
| **VIII. Food desert** | A food desert is a geographic area in which residents do not live close to a supermarket, limiting their ability to purchase healthy food such as fresh fruit and vegetables [89]. Living in a food desert raises is associated with poor health outcomes such as obesity. Interestingly, U.S. counties with higher obesity rates interestingly have lower suicide rates [90]. The United States Department of Agriculture provides the Food Access Research Atlas [91], from which we retrieved the percentage of each census tract that as of 2006 met |

| **Supplementary Table 7 continued. Social determinants of health: Small area geocode data** | |
| --- | --- |
|  |  |
| **Predictor** | **Description** |
|  | their definition of food desert. The Food Access Research Atlas defines a census-tract as a food desert if it is (1) "low income" (i.e., poverty rate is 20% or higher) and (2) either 33 percent of the population or at least 500 people live more than 1 mile from a supermarket (or 10 miles, if in a rural census tract). |
|  |  |
| **IX. Fragmentation** | Fragmentation, or a lack of a social integration in one's neighborhood, was one of the earliest geographic constructs to receive attention as a predictor of suicide [92]. More recent studies confirm that areas with higher rates of migration, private renting, people living alone, unmarried persons, residential mobility, and boarded-up vacant housing [80, 85, 93-95] have higher rates of suicide. Moreover, studies find that the effect of neighborhood fragmentation is independent of its positive association with neighborhood deprivation [85, 95]. We followed Steelesmith et al. [80] in including in our measure of fragmentation the proportion of people living alone (block group), proportion of renters (block group), proportion of unmarried persons (census tract), and proportion of residents that had moved within the past year (census tract) in each neighborhood according to the American Community Survey [74]. We also added proportion of vacant houses (block group) to the measure. We summed these variables together to create a composite fragmentation variable. |
|  |  |
| **X. Small area geographic predictors** | There is evidence that being a demographic minority relative to the composition of one’s neighborhood is a risk factor for poor mental health outcomes, including suicide [96, 97]. Accordingly, we created variables representing the interaction between the proportion of a patient’s block group composed of a given race/ethnicity and whether the patient was of that race/ethnicity. We also coded the proportion of a patient’s block group that was composed of fellow veterans. Although counties with more Veterans have higher suicide rates [80], it is possible that living among other Veterans reduces risk for suicide. All demographics data was taken the 5-year American Community Survey [74]. We obtained all American Community Survey data using the total census package in R [98]. |
|  |  |
| **XI. Violent crime rate** | Crime and homicide rates are positively correlated with suicide rates at the state level in the U.S. and the borough level in the U.K. [25, 99]. The Uniform Crime Reports (UCR), a program within the Federal Bureau of Investigation, tabulates the number of reported violent crimes (i.e., murder, nonnegligent manslaughter, non-statutory rape, robbery, and aggravated assault) in each county [100]. UCR transfers their counts to the Inter-university Consortium for Political and Social Research, who estimates county-level rates after imputing missing data. The UCR does not track crimes that were not reported to the police. For each patient visit we used the most recently available year, which ranged from two years prior to five years prior to the year of the hospitalization. |
|  |  |
| **XII. Suicide rate** | All the geographic variables considered so far are thought to explain why there is geographic variation in suicide rates. Therefore, the actual measure of geographic variation in suicide rates should also be included as an important predictor. There are 2 limitations in measuring this. First, suicide rates are only available at the county level. Second, the CDC suppresses the annual suicide rate for counties with fewer than 10 suicide deaths out of concern for confidentiality. Most counties report fewer than 20 suicides a year, which the CDC uses as a minimum cutoff for reliability [101]. Aggregating across multiple years and using small-area estimation methods (e.g., incorporating data from neighboring counties) is necessary to produce reliable estimates for counties with small populations or low suicide rates [102]. We used 5-year age-standardized county-level suicide rate estimates incorporating small-area estimation that spanned from 1980-2014 [103]. We computed 5-year estimates for each year by weighting the two 5-year intervals nearest to the focal year by their relative distance to that year. We averaged the 5-year estimates from the three most recent available years to use as a predictor in models. |
|  |  |

| **Supplementary Table 8. Physical disorders** | | |
| --- | --- | --- |
|  |  |  |
| **Predictor** | **Identifier** | **Description** |
| **I. Multi-Level Clinical Classifications Software (CCS)** |  |  |
| Physical illness | 1-4, 6-18 | The Clinical Classifications Software (CCS) is a hierarchical categorization system that groups ICD-9-CM diagnosis and procedure codes into more manageable categories. The CCS contains a single-level and multi-level classification system. There are 646 physical disease diagnosis codes in the multi-level system: 17 first level, 120 second level, 300 third level, and 209 fourth level codes. We used the multi-level system and looked at the 7 time periods before hospitalization (past 30 days, 90 days, 180 days, 365 days, 730 days, 1095 days, entire VHA history) and considered 5 treatment sectors: emergency department, psychiatric inpatient, any outpatient treatment, outpatient treatment by a mental health treatment provider, outpatient treatment by anyone other than a mental health treatment provider. We created yes/no indicators, continuous count of days, and quintiles for each of the 646 diagnosis codes. We also created yes/no indicators for each diagnosis at time of hospital admission [10]. |
|  |  |  |
| **II. Combined comorbidity score** |  |  |
| Metastatic cancer | 196.X, 197.X, 198.XX, 199.X | We used the Gagne method [104] to create a combined comorbidity score by combining the Romano approach [105] of scoring the 17 conditions in the Charlson Index [106] with van Raven’s method [107] of scoring the 30 conditions used in the Elixhauser Comorbidity Classification system [108]. Gagne et al. [104] found that this combined comorbidity scale performed better than either the Charlson or the Elixhauser in a study of 120,679 Pennsylvania Medicare enrollees. |
| Congestive heart failure | 402.01, 402.11, 402.91, 404.01, 404.03, 404.11, 404.13, 404.91, 404.93, 425.XX, 428.XX |  |
| Dementia | 331.0X, 331.1X, 331.2X, 290.XX |  |
| Renal failure | 403.11, 403.91, 404.12, 404.92, 585.X, 586, V42.0X, V45.1X, V56.0X, V56.8X |  |
| Weight loss | 260, 263.X |  |
| Hemiplegia | 342.XX, 344.XX |  |
| Alcohol abuse | 291.1, 291.2, 291.5, 291.8X, 291.9, 303.9X, 305.0X, V11.3X |  |
| Any tumor | 140.X, 141.X, 142.X, 143.X, 144.X, 145.X, 146.X, 147.X, 148.X, 149.X, 150.X, 151.X, 152.X, 153.X, 154.X, 155.X, 156.X, 157.X, 158.X, 159.X, 160.X, 161.X, 162.X, 163.X, 164.X, 165.X, 170.X, 171.X, 174.X, 175.X, 176.X, 179, 180.X, 181, 182.X, 183.X, 184.X, 185, 186.X, 187.X, 188.X, 189.X, 190.X, 191.X, 192.X, 193, 194.X, 195.X, 200.XX, 201.XX, 202.XX, 203.XX, 204.XX, 205.XX, 206.XX, 207.XX, 208.XX, 273.0, 273.3, V10.XX |  |
| Cardiac arrhythmia | 426.10, 426.11, 426.13, 426.2, 426.3, 426.4, 426.50, 426.51, 426.52, 426.53, 426.6, 426.7, 426.8X, 427.0, 427.2, 427.31, 427.60, 427.9, 785.0, V45.0X, V53.3X |  |
| Chronic pulmonary disease | 415.0, 416.8, 416.9, 491.XX, 492.X, 493.XX, 494.X, 496 |  |
| **Supplementary Table 8 continued. Physical disorders** | | |
|  |  |  |
| **Predictor** | **Identifier** | **Description** |
| Coagulopathy | 286.XX, 287.1, 287.3X, 287.4X, 287.5 |  |
| Complicated diabetes | 250.4X, 250.5X, 250.6X, 250.7X, 250.9X |  |
| Deficiency anemias | 280.X, 281.X, 285.9 |  |
| Fluid and electrolyte disorders | 276.XX |  |
| Liver disease | 070.32, 070.33, 070.54, 456.0, 456.1, 456.2X, 571.0, 571.2, 571.3, 571.4X, 571.5, 571.6, 571.8, 571.9, 572.3, 572.8, V42.7X |  |
| Peripheral vascular disorder | 440.XX, 441.2, 441.4, 441.7, 441.9, 443.XX, 447.1, 557.1, 557.9, V43.4 |  |
| Psychosis | 295.XX, 296.XX, 297.X, 298.X, 299.1X |  |
| Pulmonary circulation disorders | 416.X, 417.9 |  |
| HIV/AIDS | 042, 043, 044, V08 |  |
| Hypertension | 401.1, 401.9, 402.10, 402.90, 404.10, 405.1X, 405.9X |  |
|  |  |  |
| **III. 19 major physical health conditions** |  |  |
| Asthma | 493.XX | Previous research has found 19 major physical health conditions (asthma, back pain, cancer, congestive heart failure, COPD, diabetes, epilepsy, fibromyalgia, heart disease, HIV/AIDS, hypertension, migraine, multiple sclerosis, osteoporosis, Parkinson’s disease, psychogenic pain, renal disease, sleep disorders, TBI) to be associated with suicide [12, 109]. ICD-9-CM diagnosis codes were used to identify each condition. All codes were chosen based on Ahmedani et al. [12], except for TBI and cancer. We identified TBI using codes from Pugh et al. [23] and selected 9 specific types of cancer (head and neck, esophageal, stomach, mesothelioma, pancreatic, lung, prostate, testicular, and Hodgkin lymphoma) known to have the highest suicide rates. Zaorsky et al. [110] found the highest risk of suicide (compared to the general population) in patients diagnosed with Hodgkin lymphoma, head and neck, lung, and testicular cancers. Kam et al. [111] also reported high prevalence of suicide among patients with head and neck cancers. Henson et al. [112] reported the risk of suicide was highest in patients with mesothelioma followed by pancreatic, esophageal, lung, and stomach. Among the most common cancer cites, Saad et al. [113] found highest suicide rates within 1 year of pancreatic and lung cancer diagnosis. Prostate cancer was also associated with increased risk of suicide [114, 115]. We created a count of |
| Back pain | 720.XX, 721.XX, 722.XX, 723.X, 724.XX |  |
| Cancer |  |  |
| Head and neck | 140.X, 141.X, 142.X, 143.X, 144.X, 145.X, 146.X, 147.X, 148.X, 149.X, 160.X, 161.X, 162.0, 195.0 |  |
| Esophageal | 150.X |  |
| Stomach | 151.X |  |
| Mesothelioma | 163.X |  |
| Pancreatic | 157.X |  |
| Lung | 162.2, 162.3, 162.4, 162.5, 162.8, 162.9, 197.0 |  |
| Prostate | 185 |  |
| Testicular | 186.X |  |
| Hodgkin lymphoma | 201.XX |  |
| Congestive heart failure | 402.01, 402.11, 402.91, 404.01, 404.03, 404.11, 404.13, 404.91, 404.93, 425.XX, 428.XX |  |
| Chronic obstructive pulmonary disease | 490, 491.XX, 492.X, 494.X, 496 |  |
| Diabetes mellitus | 250.XX |  |
| Epilepsy | 345.XX |  |
| Fibromyalgia | 729.1 |  |
| **Supplementary Table 8 continued. Physical disorders** | | |
|  |  |  |
| **Predictor** | **Identifier** | **Description** |
| Heart disease | 410.XX, 411.XX, 412, 413.X, 414.XX | the number of types of cancers, indicators for each of the 19 major physical health conditions, and a count of the total number of the 19 conditions present at the 7 time periods before hospitalization (past 30 days, 90 days, 180 days, 365 days, 730 days, 1095 days, entire VHA history). |
| HIV/AIDS | 042, 043, 044, V08 |  |
| Hypertension | 401.X |  |
| Migraine | 346.XX |  |
| Multiple sclerosis | 340 |  |
| Osteoporosis | 733.0X |  |
| Parkinson's disease | 332.X |  |
| Psychogenic pain | 307.80, 307.89 |  |
| Renal disease | 403.XX, 404.02, 404.12, 404.92, 582.XX, 583.XX, 585.X, 586, 588.XX, 593.9 |  |
| Sleep disorders | 291.82, 307.4X, 327.XX, 780.5X |  |
| Traumatic brain injury | 310.2, 800.XX, 801.XX, 803.XX, 804.XX, 850.XX, 851.XX, 852.XX, 853.XX, 854.XX, 905.0, 907.0, 950.1, 950.2, 950.3, 959.01, 959.9, V15.52 |  |
|  |  |  |
| **IV. Other physical disorders** |  |  |
| Asthma | 493.XX | Asthma is a chronic condition that decreases quality of life and is a risk factor for suicide and self-harm. We created a yes/no indicator for asthma at 7 time periods before hospitalization (past 30 days, 90 days, 180 days, 365 days, 730 days, 1095 days, entire VHA history) using ICD-9-CM codes [116-120]. |
| Chronic pancreatitis | 577.1 | Chronic pancreatitis was included as it is a painful condition and is a possible predictor for suicide [121, 122]. We created an indicator at the 7 time periods. |
| Huntington’s disease | 333.4. | Huntington’s disease is a progressive neurodegenerative condition that affects motor abilities and cognition and frequently results in psychiatric symptoms, such as suicidal ideation [123]. Paulsen et al. [124] found elevated suicide risk during the period right before diagnosis (stage 1) and the period with most significant decline in functioning (stage 2). We created an indicator at the 7 time periods for HD. |
| Psoriasis | 696.1 | We included an indicator for psoriasis at the 7 time periods, despite the mixed evidence of an association with suicidality. Singhal et al. [120] found that psoriasis was associated with |
| **Supplementary Table 8 continued. Physical disorders** | | |
|  |  |  |
| **Predictor** | **Identifier** | **Description** |
|  |  | risk of self-harm but Prabhaker et al. [125] and Wu et al. [126] did not find any correlation of the sort. Though Wu et al. did find an association between depression and psoriasis. Parisi et al. [127] reported a small elevation in risk of self-harm following diagnosis and an increased risk for those with severe psoriasis. However, at the time of diagnosis, the prevalence of mental disorders and use of psychotropic medications was higher in people with psoriasis compared to the general population. Additionally, Brodalumab (SILIQ), an effective medication for psoriasis, carries a box warning and is in the Risk Evaluation and Mitigation Strategy (REMS) program due to several reported cases of suicidal ideation, behavior, and completed suicides in patients taking the drug [128, 129]. |
| ICD-9-CM V codes | V10.XX, V12.XX, V13.XX, V14.X, V16.XX, V17.XX, V18.XX, V19.XX, V41.X, V48.X, V85 | ICD-9-CM V codes provide details about circumstances, problems, or other factors that influence health status and health services [6]. We chose a subset of V codes to characterize other physical disorders, including personal history of physical illnesses and diseases. We looked at 7 time periods before hospitalization (past 30 days, 90 days, 180 days, 365 days, 730 days, 1095 days, entire VHA history) and considered 5 treatment sectors for each time period: emergency department, psychiatric inpatient, any outpatient treatment, outpatient treatment by a mental health treatment provider, outpatient treatment by anyone other than a mental health treatment provider. We created yes/no indicators and continuous count of days for each of the V codes. Additionally, we created yes/no indicators for each V code assigned during current hospital admission. |
|  |  |  |
| **V. Contact or exposure to severe infectious disease or need for vaccination** | V01.1, V01.5, V01.6, V01.79, V01.83, V01.89, V01.9, V02.53, V02.54, V02.59, V02.61, V02.62, V02.69, V02.7, V02.9, V03.2, V03.3, V03.7, V03.81, V03.82, V03.89, V04.5, V04.81, V04.89, V05.3, V05.4, V05.8, V05.9, V06.0, V06.1, V06.2, V06.4, V06.5, V06.6, V06.8 | Individuals with infections who are treated with anti-infective agents or infections that require hospitalization have an increased risk for self-harm [130]. We used 35 ICD-9-CM V codes and created 4 indicators for contact/exposure to infectious disease/need for vaccination before hospitalization: 1) Contact/exposure at 3, 12, and 24 months 2) Contact/exposure at 2 out of 3 time periods, 3) Contact/exposure at 1 time period, 4) No contact/exposure at any time period [6] |

| **Supplementary Table 8 continued. Physical disorders** | | |
| --- | --- | --- |
|  |  |  |
| **Predictor** | **Identifier** | **Description** |
| **VI. Chronic pain** | The Pain Intensity Numeric Rating Scale (PI-NRS) | Chronic pain is a well-known risk factor for suicide [16, 17, 21, 131-133]. We used scores on the Pain Intensity Numeric Rating Scale (PI-NRS) to categorize pain severity. The PI-NRS is a single item standard scale used in VA facilities to screen for chronic pain. Patients are asked to rate their current overall pain level on a scale of 0-10. A score of 4 or more on the PI-NRS indicates moderate/severe pain and a score of 7 or more indicates chronic pain, based on the definition in Goulet et al. [134]. We looked at the past 30 and 90 days and created indicators for 1 or more visits with moderate/severe pain (NRS score of 4 or more) and 1 or more visits with chronic pain (NRS score of 7 or more). We created additional indicators for 2 or more visits with moderate/severe and chronic pain in the past 365 and 730 days. |
|  |  |  |
| **VII. The Pain Condition Crosswalk** |  |  |
| Back pain | 349.39, 353.1, 353.3, 353.4, 720.XX, 721.2, 721.3, 721.4X, 721.5, 721.6, 721.7, 721.8, 721.9X, 722.1X, 722.2, 722.3X, 722.5X, 722.6, 722.70, 722.72, 722.73, 722.80, 722.82, 722.83, 722.90, 722.92, 722.93, 724.XX, 738.3, 738.4, 738.5, 738.6, 739.2, 739.3, 739.4, 739.5, 756.11, 756.12, 756.13, 756.15, 756.16, 756.17, 756.19, 839.2X, 839.4X, 846.X, 847.1, 847.2, 847.3, 847.4, 847.9, 848.5 | The Pain Condition ICD-9-CM to ICD-10-CM Crosswalk contains 13 pain-related diagnostic clusters: 1) back pain, 2) neck pain, 3) limb/extremity pain, joint pain and nonsystemic, noninflammatory arthritic disorders, 4) fibromyalgia, 5) headache, 6) orofacial, ear, and temporomandibular disorder pain, 7) abdominal and bowel pain, 8) urogenital, pelvic, and menstrual pain, 9) musculoskeletal chest pain, 10) neuropathy, 11) systemic disorders or diseases causing pain, 12) other painful conditions, and 13) fractures, contusions, sprains, and strains. These diagnostic clusters were created based on other pain clusters and are a reliable mapping of pain conditions from ICD-9-CM and ICD-10-CM [135]. We used ICD-9-CM codes from Mayhew et al. [135] to categorize each pain condition and counted the number of days with each of the 13 pain-related diagnostic clusters. We also created and indicator for any of the 13 clusters in the 7 time periods before hospitalization (past 30 days, 90 days, 180 days, 365 days, 730 days, 1095 days, entire VHA history). |
| Neck pain | 353.2, 721.0, 721.1, 722.0, 722.4, 722.71, 722.81, 722.91, 723.X, 738.2, 739.1, 839.0X, 847.0, 848.2 |  |
| Limb/extremity pain, joint pain and non-systemic, non-inflammatory arthritic disorders | 274.XX, 353.0, 353.5, 353.6, 354.0, 354.4, 355.0, 355.1, 355.2, 355.3, 355.4, 355.5, 355.6, 355.7, 355.71, 712.XX, 713.1, 713.5, 714.4, 715.XX, 716.1X, 716.2X, 716.3X, 716.4X, 716.5X, 716.6X, 716.8X, 716.9X, 717.7, 717.81, 717.82, 717.83, 717.84, 717.85, 718.0X, 718.3X, 718.4X, 718.5X, 719.4X, 726.XX, 727.01, 727.03, 727.04, 727.05, 727.06, 727.09, 727.1, 727.2, 727.3, 727.40, 727.49, 727.51, 727.6X, 727.8X, 727.9, 728.4, 728.71, 728.85, 728.89, 729.5, 733.0X, 733.99, 739.6, 739.7, 755.67, 755.9, 830.1, 831.1X, 832.00, 832.1X, 833.1X, 834.1X, 835.1X, 836.4, 836.50, 836.6X, 837.1, 838.1X, 839.1X, 839.3X, 839.5X, 839.7X, 839.9, 905.6 |  |
| Fibromyalgia | 729.1 |  |
| Headache | 307.81, 346.XX, 339.0X, 339.1X, 339.2X, 339.4X, 339.8X, 784.0 |  |
| Orofacial, ear, and temporomandibular disorder pain | 350.2, 379.91, 524.6X, 526.89, 526.9, 739.0, 784.92, 830.0, 848.1 |  |
| Abdominal and bowel pain | 550.1X, 550.9X, 552.0X, 553.00, 553.01, 553.1, 553.2X, 553.3, 553.8, 553.9, 564.1, 574.XX, 592.0, 592.1, 739.9, 789.0X |  |
| **Supplementary Table 8 continued. Physical disorders** | | |
|  |  |  |
| **Predictor** | **Identifier** | **Description** |
| Urogenital, pelvic and menstrual pain | 098.12, 098.32, 131.03, 592.9, 594.X, 595.1, 601.0, 601.1, 601.4, 601.9, 602.0, 608.89, 608.9, 614.3, 614.4, 614.5, 614.7, 614.8, 614.9, 615.0, 615.1, 615.9, 616.0, 617.X, 625.0, 625.1, 625.2, 625.3, 625.4, 625.5, 625.7X, 625.8, 625.9 |  |
| Musculoskeletal chest pain | 411.1, 413.0, 413.1, 413.9, 733.6, 739.8, 786.50, 786.52, 786.59, 839.61, 848.3, 848.4X |  |
| Neuropathy | 053.12, 053.13, 250.6X, 337.00, 337.09, 337.1, 350.1, 350.8, 350.9, 352.1, 353.8, 353.9, 354.8, 354.9, 355.79, 355.8, 355.9, 356.4, 356.8, 356.9, 357.2, 377.33, 377.34, 377.39, 377.41, 729.2 |  |
| Systemic disorders or diseases causing pain | 026.1, 088.81, 282.41, 282.42, 282.5, 282.60, 282.63, 282.64, 282.68, 282.69, 337.20, 710.X, 711.1X, 711.2X, 714.0, 714.1, 714.2, 714.3X, 714.8X, 714.9, 719.3X, 725, 729.0, 729.4, 730.7X |  |
| Other painful conditions | 003.23, 003.24, 036.82, 056.71, 307.89, 327.52, 333.94, 338.XX, 567.31, 711.0X, 711.3X, 711.4X, 711.5X, 711.6X, 711.7X, 711.8X, 711.9X, 713.7, 726.13, 726.63,  727.00, 727.02, 727.41, 727.42, 727.43, 728.0, 728.1X 728.2, 728.3, 728.5, 728.6, 728.79, 728.81, 728.82, 728.83, 728.84, 728.86, 728.87, 728.88, 728.9,729.3X, 729.6, 729.7X, 729.8X, 729.9X, 730.0X, 730.1X, 730.2X, 730.3X, 730.8X, 730.9X, 731.X, 732.X, 733.2X, 733.3, 733.4X, 733.5, 733.7, 733.8X, 733.90, 733.91, 733.92, 734, 735.X, 736.XX, 737.XX, 738.0, 738.1X, 738.7, 738.8, 738.9,  742.5X, 756.10, 756.14, 780.96, 781.91, 781.92, 781.94, 781.99, 793.7, 806.XX, 907.2, 952.XX, V13.59, V42.4, V43.6X, V43.7, V45.4, V48.1, V48.2, V48.3, V48.6, V48.7, V49.0, V49.1, V49.2, V49.4, V49.5, V49.6X, V49.7X, V53.7, V88.2X |  |
| Fractures, contusions, sprains and strains (conditions commonly associated with acute pain) | 713.0, 713.2, 713.3, 713.4, 713.6, 713.8, 716.0X, 717.0, 717.1, 717.2, 717.3, 717.4X, 717.5, 717.6, 717.89, 717.9, 718.1X, 718.2X, 718.6X, 718.7X, 718.8X, 718.9X, 719.0X, 719.1X, 719.2X, 719.5X, 719.6X, 719.7X, 719.8X, 719.9X, 727.50, 727.59, 733.1X, 733.93, 733.94, 733.95, 733.96, 733.97, 733.98, 800.0X, 800.5X, 801.0X, 801.5X, 802.XX, 803.0X, 803.5X, 804.0X, 804.5X, 805.XX, 807.XX, 808.XX, 809.1, 810.XX, 811.XX, 812.XX, 813.XX, 814.XX, 815.XX, 816.XX, 817.X, 818.X, 819.X, 820.XX, 821.XX, 822.X, 823.XX, 824.X, 825.XX, 826.X, 827.X, 828.X, 829.X, 831.0X, 832.01, 832.02, 832.03, 832.04, 832.09, 832.2, 833.0X, 834.0X, 835.0X, 836.0, 836.1, 836.2, 836.3, 836.51, 836.52, 836.53, 836.54, 836.59, 837.0, 838.0X, 839.69, 839.8, 840.X, 841.X, 842.XX, 843.X, 844.X, 845.XX, 848.0, 848.8, 848.9, 905.0, 905.1, 905.2, 905.3, 905.4, 905.5, 905.7, 920, 921.X, 922.XX, 923.XX, 924.XX, 959.0X, 959.1X, 959.8, 959.9, V13.4, V13.51, V13.52, V54.0X, V54.1X, V54.2X, V66.4, V67.4, |  |
|  |  |  |
| **VIII. Chronic Overlapping Pain Conditions (COPCs)** |  |  |
| Fibromyalgia | 729.1 | In 2015, the National Institutes of Health coined the term “Chronic Overlapping Pain Conditions (COPC)” to describe a |
| **Supplementary Table 8 continued. Physical disorders** | | |
|  |  |  |
| **Predictor** | **Identifier** | **Description** |
| Irritable bowel syndrome | 564.1 | cluster of 10 chronic and painful conditions that often occur together and share similar symptoms and features [136]. The 10 COPCs are: Fibromyalgia, IBS, interstitial cystitis/Bladder pain syndrome, vulvodynia, migraine, chronic tension-type headache, temporomandibular disorder, chronic fatigue syndrome, endometriosis with pain, and chronic low back pain. The complexity and overlap of these conditions make them difficult to recognize, diagnose, and treat, especially if someone is suffering from multiple at the same time. Suffering from any one of these conditions alone increases the risk of suicide, let alone having more than 2 at the same time [137-139]. We converted the COPC ICD-10 codes from Schrepf et al. [138] to ICD-9-CM using Alkaline Software [140]. We created yes/no indicators for each of the 10 types of conditions and a count of the number of conditions in the 7 time periods before hospitalization. We also created a yes/no indicator for 2 or more in past 30 days and 2 or more in past 730 days. |
| Interstitial cystitis/Bladder pain syndrome | 595.1, 595.3 |  |
| Vulvodynia | 625.70, 625.71, 625.79 |  |
| Migraine | 346.0X, 346.1X, 346.2X, 346.3X, 346.4X, 346.5X, 346.7X, 346.8X, 346.9X |  |
| Chronic tension-type headache | 307.81, 339.1X |  |
| Temporomandibular disorder | 339.89, 524.60, 524.62, 524.63, 830.0 |  |
| Chronic fatigue syndrome | 780.71 |  |
| Endometriosis with pain |  |  |
| Endometriosis | 617.X |  |
| Pain | 625.0, 625.3, 625.9, 789.09 |  |
| Chronic low back pain | 307.89, 718.88, 721.5, 721.6, 721.7, 721.90, 722.10, 722.32, 722.52, 722.73, 722.8X, 722.90, 722.93, 724.01, 724.02, 724.2, 724.3, 724.5, 724.6, 724.8, 739.3, 739.4, 756.12, 756.13, 756.14, 756.15, 756.16, 756.17, 756.19, 805.4, 805.6, 846.0, 846.1, 846.9, 847.2, 847.9, 848.5 |  |
|  |  |  |
| **IX. Severe injury** |  |  |
| Amputations | 885.X, 886.X, 887.X, 895.X, 896.X, 897.X | We considered 5 severe injuries, as they are important risk factors for suicide: amputations, burns, serious eye injuries and blindness, hearing loss, and spinal cord injuries resulting in paralysis. We used ICD-9-CM E codes and diagnosis codes and created yes/no indicators for each injury, a count of the number of different injuries, and an indicator of any severe injury at the 7 time periods before hospitalization [8, 141-144]. |
| Burns | 948.XX, 949.XX, E990.0, E990.1, E990.2, E990.3 |  |
| Serious eye injuries and blindness | 369.XX, 871.X, 950.X |  |
| Hearing loss | 388.12, 388.2, 389.XX |  |
| Spinal cord injuries and resulting paralysis | 342.XX, 344.XX, 780.72, 806.XX, 952.XX |  |
|  |  |  |
| **X. VA National Formulary Drug Classification – Non-psychotropic medications** |  |  |
| Antidotes, deterrents, and poison control (AD000) | AD100, AD200, AD300, AD400, AD900 | Non-psychotropic medications can have psychiatric-related side effects, such as depression, psychosis, suicidal ideation, and can increase the risk of suicidality [145-151]. Our dataset contained a total of 9,023 individual non-psychotropic medications/products from the VA National |
| Antihistamines (AH000) | AH100, AH102, AH103, AH104, AH105, AH106, AH107, AH109 |  |
| Antimicrobials (AM000) | AM114, AM110, AM111, AM112, AM113, AM115, AM116, AM117, AM118, AM119, AM150, AM200, AM250, AM300, AM350, AM400, AM500, AM550, AM600, AM650, AM700, AM800, AM900 |  |

| **Supplementary Table 8 continued. Physical disorders** | | |
| --- | --- | --- |
|  |  |  |
| **Predictor** | **Identifier** | **Description** |
| Antineoplastics (AN000) | AN100, AN200, AN300, AN400, AN500, AN600, AN700, AN900 | Formulary (VANF), which is listing of drugs and supplies available at all VA facilities. As of October 2019, the VANF contained 29,290 individual pharmaceutical products, divided into 32 major drug classes, 287 minor drug classes, and 255 sub-classes [35]. We created count variables for the total number of drugs prescribed in each of the major non-psychotropic drug classes in the past 90 and 365 days [152]. We coded the Central Nervous System medication class differently (see above for details) and we did not create variables for the Prosthetics/Supplies/Devices (XA000) and Miscellaneous Agents (XX000) classes. |
| Antiparasitics (AP000) | AP100, AP101, AP109, AP200, AP300, AP900 |  |
| Antiseptics/disinfectants (AS000) | AS000 |  |
| Autonomic medications (AU000) | AU100, AU200, AU300, AU350, AU900 |  |
| Blood products/modifiers/volume expanders (BL000) | BL110, BL115, BL116, BL117, BL118, BL400, BL500, BL800, BL900 |  |
| Cardiovascular medications (CV000) | CV050, CV100, CV150, CV200, CV250, CV300, CV350, CV400, CV490, CV500, CV600, CV700, CV701, CV702, CV703, CV704, CV709, CV800, CV805, CV806, CV900 |  |
| Dermatological agents (DE000) | DE100, DE101, DE102, DE103, DE109, DE200, DE250, DE300, DE350, DE400, DE450, DE500, DE600, DE650, DE700, DE750, DE751, DE752, DE800, DE810, DE820, DE890, DE900 |  |
| Diagnostic agents (DX000) | DX100, DX101, DX102, DX109, DX200, DX201, DX202, DX300, DX900 |  |
| Gastrointestinal medications (GA000) | GA100, GA101, GA102, GA103, GA104, GA105, GA106, GA107, GA108, GA109, GA110, GA199, GA200, GA201, GA202, GA203, GA204, GA205, GA206, GA208, GA209, GA300, GA301, GA302, GA303, GA309, GA400, GA500, GA600, GA605, GA750, GA751, GA752, GA759, GA800, GA801, GA802, GA900 |  |
| Genitourinary medications (GU000) | GU100, GU200, GU201, GU209, GU300, GU400, GU500, GU600, GU900 |  |
| Herbs/alternative therapies (HA000) | HA000 |  |
| Hormones/synthetics/modifiers (HS000) | HS050, HS051, HS052, HS100, HS200, HS300, HS400, HS500, HS501, HS502, HS503, HS509, HS600, HS700, HS701, HS702, HS800, HS850, HS851, HS852, HS875, HS900 |  |
| Immunological agents (IM000) | IM100, IM105, IM109, IM300, IM400, IM500, IM600, IM700, IM900 |  |
| Investigational agents (non-CNS) (IN000) | IN001, IN002, IN003, IN004, IN005, IN100, IN110, IN120, IN130, IN140, IN150, IN160, IN170, IN180, IN200, IN210, IN220, IN230, IN240, IN250, IN260, IN270, IN280, IN300, IN350, IN400, IN410, IN420, IN430, IN505, IN510, IN515, IN540, IN560, IN585, IN600, IN610, IN620, IN630, IN640, IN650, IN660, IN665, IN670, IN675, IN700, IN710, IN720, IN800, IN810, IN820, IN830, IN840, IN850, IN860, IN870, IN880, IN900, IN910, IN920, IN930, IN940, IN950, IN960, IN970, IN980, IN999 |  |
| Intrapleural medications (IP000) | IP100, IP900 |  |
| Irrigation/dialysis solutions (IR000) | IR100, IR200, IR300, IR900 |  |
| Musculoskeletal medications (MS000) | MS100, MS101, MS102, MS130, MS140, MS150, MS160, MS190, MS200, MS205, MS300, MS400, MS900 |  |

| **Supplementary Table 8 continued. Physical disorders** | | |
| --- | --- | --- |
|  |  |  |
| **Predictor** | **Identifier** | **Description** |
| Nasal and throat agents, topical (NT000) | NT100, NT200, NT300, NT400, NT900 |  |
| Ophthalmic agents (OP000) | OP100, OP101, OP102, OP103, OP105, OP107, OP109, OP140, OP160, OP200, OP210, OP219, OP220, OP230, OP300, OP350, OP400, OP500, OP600, OP700, OP800, OP900 |  |
| Dental and oral agents, topical (OR000) | OR100, OR200, OR300, OR400, OR500, OR900 |  |
| Otic agents (OT000) | OT100, OT101, OT102, OT109, OT200, OT250, OT300, OT400, OT900 |  |
| Pharmaceutical aids/reagents (PH000) | PH000 |  |
| Respiratory tract medications (RE000) | RE100, RE101, RE102, RE103, RE104, RE105, RE108, RE109, RE200, RE300, RE301, RE302, RE400, RE500, RE501, RE502, RE503, RE504, RE505, RE506, RE507, RE508, RE509, RE510, RE511, RE512, RE513, RE514, RE515, RE516, RE599, RE600, RE900 |  |
| Rectal, local (RS000) | RS100, RS200, RS201, RS202, RS300, RS900 |  |
| Therapeutic nutrients/minerals/electrolytes (TN000) | TN100, TN101, TN102, TN200, TN300, TN400, TN410, TN420, TN430, TN440, TN450, TN460, TN470, TN475, TN476, TN478, TN490, TN499, TN500, TN501, TN502, TN503, TN509, TN900 |  |
| Vitamins (VT000) | VT050, VT100, VT101, VT102, VT103, VT104, VT105, VT106, VT107, VT109, VT400, VT500, VT501, VT502, VT503, VT504, VT509, VT600, VT700, VT701, VT702, VT709, VT800, VT801, VT802, VT809, VT900 |  |
|  |  |  |
| **XI. Vitamin D** | Calcifediol, calcitriol, cholecalciferol, clozapine, dihydrotachysterol, doxercalciferol, ergocalciferol, olanzapine, paricalcitol, quetiapine, vitamin d | Vitamin D deficiency has been associated with depression and other mental and physical disorders that are frequently co-morbid with suicide and self-harm. According to Tariq et al. [153], increasing vitamin D levels may have the potential to decrease suicidality. We created an indicator for prescribed vitamin D in past 90 and past 365 days. |
|  |  |  |

| **Supplementary Table 9. Medications Classified by FDA as Increased Risk of Suicide** | | |
| --- | --- | --- |
|  |  |  |
| **Predictor** | **VA Drug Class/Medication Names** | **Description** |
| **I. FDA approved drug labels** |  |  |
| Boxed warning | amitriptyline, amoxapine, aripiprazole, atomoxetine, brexpiprazole, brodalumab, bupropion, cariprazine, chlordiazepoxide and amitriptyline, citalopram, clomipramine, desipramine, desvenlafaxine, deutetrabenazine, doxepin, duloxetine, escitalopram, esketamine nasal spray, fluoxetine,fluoxetine and olanzapine, fluvoxamine, imipramine, isocarboxazid, levomilnacipran, lurasidone, maprotiline, milnacipran, mirtazapine, naltrexone hydrochloride and bupropion hydrochloride, nefazodone hydrochloride, nortriptyline, paroxetine, peginterferon alfa-2b, perphenazine and amitriptyline hcl, phenelzine, propoxyphene, propoxyphene and acetaminophen, protriptyline, quetiapine, rasagiline mesylate, selegiline, sertraline, tetrabenazine, tranylcypromine, trazodone, trimipramine, venlafaxine, vilazodone, vortioxetine | The U.S. Food and Drug Administration requires that drug manufacturers list adverse side effects on the drug product labels (including the package inserts). There are 3 sections that address adverse drug reactions, in order of severity level: Boxed warnings, warnings and precautions, and adverse reactions [154]. The box warning section describes “certain contraindications or serious warnings, particularly those that may lead to death or serious injury”; the warnings and precautions section describes “clinically significant adverse reactions (including any that are potentially fatal, are serious even if infrequent, or can be prevented or mitigated through appropriate use of the drug)”; and the adverse reactions section describes “the overall adverse reaction profile of the drug based on the entire safety database…an adverse reaction is an undesirable effect, reasonably associated with use of a drug, that may occur as part of the pharmacological action of the drug or may be unpredictable in its occurrence” [155]. We searched FDA-approved drug labeling documents for suicide as adverse drug reaction using the FDA Label Database [37]. Specific search terms were “suicidality, suicidal behavior, suicidal ideation, suicide attempt, suicidal, and suicide” [156-158]. We found 49 drugs that indicated suicide as an adverse reaction in the box warning section of the drug label, 112 drugs in the warnings and precautions section, and 79 in adverse reactions. We created variables for the number of drugs prescribed with each type of warning and a count of the number of drugs prescribed with any FDA warning in past 90 days and 365 days. |
| Warnings & precautions | acamprosate, alprazolam, amantadine, apremilast, armodafinil, belimumab, betamethasone sodium phosphate and betamethasone acetate, brexanolone, brivaracetam, cannabidiol, carbamazepine, carbidopa and entacapone and levodopa, chlordiazepoxide, ciprofloxacin, clobazam, clonazepam, clorazepate, cyclo/gaba 10/300 pack, daclizumab, delafloxacin meglumine, dexmethylphenidate hydrochloride, dextroamphetamine saccharate/sulfate and amphetamine aspartate and dextroamphetamine sulfate and amphetamine sulfate, dextroamphetamine sulfate, diazepam, divalproex, dolutegravir sodium and rilpivirine hydrochloride, efavirenz, efavirenz and emtricitabine and tenofovir, efavirenz and lamivudine and tenofovir, elagolix, emtricitabine and rilpivirine hydrochloride and tenofovir, eslicarbazepine, estazolam, eszopiclone, ethosuximide, ethotoin, felbamate, flurazepam, gabapentin, gabapentin and .gamma.-aminobutyric acid, gabapentin and lidocaine hydrochloride, gemifloxacin, hydralazine and hydrochlorothiazide and reserpine, hydroxychloroquine sulfate, iloperidone, interferon alfa-2b, interferon beta-1a, interferon beta-1b, isotretinoin, lacosamide, lamotrigine, levetiracetam, levofloxacin, levonorgestrel and ethinyl estradiol (FROM 11/13/2018 LABEL; REMOVED FROM LATEST VERSION); liraglutide (FROM 10/19/2018 LABEL; REMOVED FROM LATEST VERSION), lisdexamfetamine, lorazepam, lorcaserin, lorlatinib, magesium citrate and bisacodyl and petrolatum and polyethylene glycol 3350 and metoclopramide, mefloquine, methamphetamine hydrochloride, methsuximide, methylphenidate, metoclopramide, midazolam, modafinil, montelukast, moxifloxacin, naltrexone, olanzapine, oxcarbazepine, paliperidone, peginterferon alfa-2a, peginterferon beta-1a, pentobarbital, perampanel, pergolide, perphenazine, phentermine and topiramate, phenytoin, polythiazide and reserpine, pregabalin, primidone, prucalopride, quazepam, ramelteon, ribavirin, rilpivirine, roflumilast, rufinamide, secobarbital, sodium oxybate, stiripentol, suvorexant, tafenoquine, tegaserod, temazepam, testosterone enanthate, tiagabine, topiramate, tramadol and acetaminophen, triazolam, trimethadione, valproic acid, varenicline, vigabatrin, zaleplon, ziconotide, ziprasidone, zolpidem, zonisamide |  |
| Adverse reactions | abacavir sulfate and dolutegravir sodium and lamivudine, acitretin, aldesleukin, alemtuzumab, amlodipine and atorvastatin, amphetamine sulfate, asenapine, |  |
| **Supplementary Table 9. Medications Classified by FDA as Increased Risk of Suicide** | | |
|  |  |  |
| **Predictor** | **VA Drug Class/Medication Names** | **Description** |
|  | atorvastatin calcium trihydrate, baclofen, beclomethasone dipropionate, bictegravir sodium and emtricitabine and tenofovir alafenamide fumarate, blinatumomab, bortezomib, buspirone hydrochloride, carbidopa, carbidopa and levodopa, celecoxib, certolizumab pegol, cetirizine hydrochloride (FROM 7/19/19 LABEL; REMOVED FROM LATEST VERSION), chloroquine phosphate (FROM 10/09/2018 LABEL; REMOVED FROM LATEST VERSION), cycloserine, dapsone (FROM 05/13/2019 LABEL; REMOVED FROM LATEST VERSION), deflazacort, dolutegravir sodium, dolutegravir sodium and lamivudine, doravirine, doravirine and lamivudine and tenofovir, elvitegravir and cobicistat and emtricitabine and tenofovir alafenamide, enfuvirtide, galantamine hydrobromide (FROM 06/13/2016 LABEL; REMOVED FROM LATEST VERSION), glatiramer acetate, histrelin acetate, human papillomavirus 9-valent vaccine recombinant, human papillomavirus quadrivalent (types 6, 11, 16, and 18) vaccine recombinant, hydromorphone (FROM 3/31/2019 LABEL; REMOVED FROM LATEST VERSION), imiquimod, ketoconazole, ledipasvir and sofosbuvir, leuprolide (FROM 03/12/2019 LABEL; REMOVED FROM LATEST VERSION), leuprolide acetate and norethindrone acetate, levocetirizine dihydrochloride (FROM 07/15/2019 LABEL; REMOVED FROM LATEST VERSION), levorphanol tartrate, memantine, memantine hydrochloride and donepezil hydrochloride, meningococcal (groups a, c, y and w-135) oligosaccharide diphtheria crm197 conjugate vaccine, nafarelin acetate, natalizumab, nelfinavir mesylate, norethindrone acetate and ethinyl estradiol, norethindrone acetate and ethinyl estradiol and ferrous fumarate, octreotide acetate (FROM 04/11/2019 LABEL; REMOVED FROM LATEST VERSION), ofloxacin (FROM 05/30/2019 LABEL; REMOVED FROM LATEST VERSION), ombitasvir and paritaprevir and ritonavir, oxaliplatin, oxazepam, oxycodone and acetaminophen, oxycodone hydrochloride, pramipexole dihydrochloride, prasterone and ibuprofen, progesterone, quinine sulfate, raltegravir, rifapentine, rivastigmine (FROM 01/08/2015 LABEL; REMOVED FROM LATEST VERSION), ropinirole hydrochloride (FROM 03/26/2015 LABEL; REMOVED FROM LATEST VERSION), saquinavir mesylate, sofosbuvir, sumatriptan succinate, sumatriptan succinate and mentholum and belladonna and iris versicolor and sanguinaria canadensis, tapentadol, thalidomide, thiotepa, tizanidine hydrochloride (FROM 01/01/2019 LABEL; REMOVED FROM LATEST VERSION), tramadol, tramadol hydrochloride and .gamma.-aminobutyric acid, tramadol hydrochloride and gaba, triptorelin, valbenazine, voriconazole |  |
|  |  |  |

| **Supplementary Table 10. Medical procedures** | | |
| --- | --- | --- |
|  |  |  |
| **Predictor** | **Identifier** | **Description** |
| **I. Physical, occupational, vocational, and rehabilitation therapy** | V57.1, V57.21, V57.22, V57.3, V57.89, V57.9 | Rehabilitation therapy typically indicates the presence of a chronic physical health problem, particularly if the frequency of visits is high. As noted above, chronic physical illnesses are significant predictors of suicide. We created 4 indicators for physical/occupational therapy using 6 ICD-9-CM V codes: 1) Therapy at 3, 12, and 24 months 2) Therapy at 2 out of 3 time periods, 3) Therapy at 1 time period, 4) No therapy at any time period [6]. We also created indicators at the 7 time periods. |
|  |  |  |
| **II. Gastric bypass surgery** | 43846 | There is an elevated risk for self-harm following gastric bypass [159-161]. We used the Current Procedural Terminology (CPT) code for gastric bypass surgery to create an indicator at the 7 time periods. |
|  |  |  |
| **III. Screenings & examinations** | V67.XX, V70.X, V71.XX, V72.XX, V73.XX, V74.X, V75.X, V76.XX, V77.XX, V78.X, V79.X, V80.XX, V81.X, V82.XX | ICD-9-CM V codes provide details about circumstances, problems, or other factors that influence health status and health services [6]. We chose a subset of V codes to characterize procedures, screenings, other care, and problems associated with medical care that may be associated with suicide [3, 34, 162, 163]. For example, Vázquez et al. [142] found a high prevalence of depression and suicidal behavior after limb amputations. We looked at 7 time periods before hospitalization (past 30 days, 90 days, 180 days, 365 days, 730 days, 1095 days, entire VHA history) and considered 5 treatment sectors for each time period: emergency department, psychiatric inpatient, any outpatient treatment, outpatient treatment by a mental health treatment provider, outpatient treatment by anyone other than a mental health treatment provider. We created yes/no indicators and continuous count of days for each of the 16 V codes. Additionally, we created yes/no indicators for each V code assigned during current hospital admission. |
| **IV. Counseling** | V65.4X |  |
| **VI. Reproduction and development** | V22.X, V23.X, V24.X, V25.XX, V26.XX, V27.X, |  |
| **VI. Other procedures, care, or medical encounters** | V42.XX, V43.XX, V44.XX, V45.XX, V46.XX, V47.X, V49.XX, V50.XX, V51.X, V52.X, V53.XX, V54.XX, V55.X, V56.XX, V57.XX, V58.XX, V59.XX, V63.X, V64.XX, V66.X, V68.XX, V87.XX, V88.XX |  |
|  |  |  |

| **Supplementary Table 11. Sample distribution on stratification variables** | | | | | | | | | | | |
| --- | --- | --- | --- | --- | --- | --- | --- | --- | --- | --- | --- |
|  | | | | | | | | | | | |
|  | **Training sample**  **(January 1, 2010 - October 22, 2012)** | | | | |  | **Holdout sample**  **(October 23, 2012 - December 31, 2013)** | | | | |
|  | **Psychiatric hospitalizations**  **(n = 273,860)** | |  | **All VHA visits**  **(n = 196,986,095)** | |  | **Psychiatric hospitalizations**  **(n = 117,158)** | |  | **All VHA visits**  **(n = 89,109,223)** | |
|  | **%** | **(SE)** |  | **%** | **(SE)** |  | **%** | **(SE)** |  | **%** | **(SE)** |
| **Age** |  |  |  |  |  |  |  |  |  |  |  |
| 20-40 | 19.0 | (0.1) |  | 8.3 | (0.0) |  | 19.8 | (0.1) |  | 8.5 | (0.0) |
| 41-50 | 18.7 | (0.1) |  | 9.7 | (0.0) |  | 19.1 | (0.1) |  | 9.2 | (0.0) |
| 51-55 | 20.0 | (0.1) |  | 9.6 | (0.0) |  | 16.5 | (0.1) |  | 9.0 | (0.0) |
| 56-61 | 20.2 | (0.1) |  | 17.1 | (0.0) |  | 20.4 | (0.1) |  | 14.7 | (0.0) |
| 62+ | 22.1 | (0.1) |  | 55.2 | (0.0) |  | 24.1 | (0.1) |  | 58.5 | (0.0) |
| **Sex** |  |  |  |  |  |  |  |  |  |  |  |
| Female | 7.4 | (0.0) |  | 6.5 | (0.0) |  | 6.6 | (0.1) |  | 7.1 | (0.0) |
| Male | 92.6 | (0.0) |  | 93.5 | (0.0) |  | 93.4 | (0.1) |  | 92.9 | (0.0) |
| **Race/ethnicity** |  |  |  |  |  |  |  |  |  |  |  |
| Non-Hispanic White | 60.1 | (0.1) |  | 64.7 | (0.0) |  | 61.3 | (0.1) |  | 65.2 | (0.0) |
| Non-Hispanic Black | 24.9 | (0.1) |  | 20.6 | (0.0) |  | 24.3 | (0.1) |  | 20.8 | (0.0) |
| Hispanic | 9.9 | (0.1) |  | 7.1 | (0.0) |  | 9.0 | (0.1) |  | 6.8 | (0.0) |
| Other (including missing) | 5.1 | (0.0) |  | 7.6 | (0.0) |  | 5.4 | (0.1) |  | 7.2 | (0.0) |
| **Marital status** |  |  |  |  |  |  |  |  |  |  |  |
| Married | 26.0 | (0.1) |  | 49.6 | (0.0) |  | 24.3 | (0.1) |  | 49.4 | (0.0) |
| Divorced (including previously married and missing) | 38.8 | (0.1) |  | 27.7 | (0.0) |  | 38.4 | (0.1) |  | 28.0 | (0.0) |
| Separated | 8.5 | (0.1) |  | 3.8 | (0.0) |  | 23.3 | (0.1) |  | 3.8 | (0.0) |
| Widowed | 3.8 | (0.0) |  | 6.2 | (0.0) |  | 3.5 | (0.1) |  | 6.3 | (0.0) |
| Never married | 23.0 | (0.1) |  | 12.6 | (0.0) |  | 23.3 | (0.1) |  | 12.4 | (0.0) |
| **Income** |  |  |  |  |  |  |  |  |  |  |  |
| No income | 18.4 | (0.1) |  | 13.3 | (0.0) |  | 18.1 | (0.1) |  | 13.8 | (0.0) |
| Low | 13.7 | (0.1) |  | 14.3 | (0.0) |  | 14.1 | (0.1) |  | 14.5 | (0.0) |
| Low-average | 27.2 | (0.1) |  | 23.9 | (0.0) |  | 26.9 | (0.1) |  | 24.5 | (0.0) |
| High-average | 15.9 | (0.1) |  | 28.1 | (0.0) |  | 15.8 | (0.1) |  | 29.9 | (0.0) |
| High | 24.9 | (0.1) |  | 20.4 | (0.0) |  | 25.1 | (0.1) |  | 17.4 | (0.0) |
| **Religion** |  |  |  |  |  |  |  |  |  |  |  |
| Baptist | 28.2 | (0.1) |  | 28.7 | (0.0) |  | 24.1 | (0.1) |  | 28.2 | (0.0) |
| **Supplementary Table 11 continued. Sample distribution on stratification variables** | | | | | | | | | | | |
|  | | | | | | | | | | | |
|  | **Training sample**  **(January 1, 2010 - October 22, 2012)** | | | | |  | **Holdout sample**  **(October 23, 2012 - December 31, 2013)** | | | | |
|  | **Psychiatric hospitalizations**  **(n = 273,860)** | |  | **All VHA visits**  **(n = 196,986,095)** | |  | **Psychiatric hospitalizations**  **(n = 117,158)** | |  | **All VHA visits**  **(n = 89,109,223)** | |
|  | **%** | **(SE)** |  | **%** | **(SE)** |  | **%** | **(SE)** |  | **%** | **(SE)** |
| Black Baptist | 9.7 | (0.1) |  | 7.7 | (0.0) |  | 9.8 | (0.1) |  | 10.0 | (0.0) |
| Evangelical and other Baptist | 18.5 | (0.1) |  | 21.0 | (0.0) |  | 14.3 | (0.1) |  | 18.2 | (0.0) |
| Other Protestant | 18.9 | (0.1) |  | 19.8 | (0.0) |  | 19.6 | (0.1) |  | 18.9 | (0.0) |
| Roman Catholic | 19.8 | (0.1) |  | 20.8 | (0.0) |  | 18.9 | (0.1) |  | 19.9 | (0.0) |
| Other Christian (including Jehovah's Witnesses, Latter Day Saints, non-Specific Christian, Orthodox) | 6.4 | (0.0) |  | 4.5 | (0.0) |  | 10.2 | (0.1) |  | 5.9 | (0.0) |
| Other non-Christian (including Buddhism, Hindu, Islam, Jewish) | 6.9 | (0.0) |  | 4.5 | (0.0) |  | 6.1 | (0.1) |  | 4.4 | (0.0) |
| None | 19.9 | (0.1) |  | 16.5 | (0.0) |  | 21.1 | (0.1) |  | 17.6 | (0.0) |
| **Census region** |  |  |  |  |  |  |  |  |  |  |  |
| Northeast (including Puerto Rico & US Virgin Islands) | 19.2 | (0.1) |  | 14.4 | (0.0) |  | 19.7 | (0.1) |  | 14.3 | (0.0) |
| Midwest | 21.0 | (0.1) |  | 22.5 | (0.0) |  | 20.6 | (0.1) |  | 22.6 | (0.0) |
| South | 41.0 | (0.1) |  | 42.0 | (0.0) |  | 42.0 | (0.1) |  | 42.2 | (0.0) |
| West (including American Samoa, Guam, Northern Mariana Islands) | 18.7 | (0.1) |  | 21.1 | (0.0) |  | 17.7 | (0.1) |  | 21.0 | (0.0) |
| **Urbanicity** |  |  |  |  |  |  |  |  |  |  |  |
| Metro area with >1m population | 51.4 | (0.1) |  | 46.0 | (0.0) |  | 53.5 | (0.1) |  | 46.4 | (0.0) |
| Metro area with 250k - 1m population | 23.8 | (0.1) |  | 24.4 | (0.0) |  | 23.1 | (0.1) |  | 24.2 | (0.0) |
| Metro area with <250k population | 9.0 | (0.1) |  | 11.0 | (0.0) |  | 10.2 | (0.1) |  | 11.1 | (0.0) |
| Urban area with ≥20k population | 5.9 | (0.0) |  | 7.4 | (0.0) |  | 5.1 | (0.1) |  | 7.3 | (0.0) |
| Urban/rural area with <20k population | 9.8 | (0.1) |  | 11.2 | (0.0) |  | 8.0 | (0.1) |  | 11.0 | (0.0) |
| **Homelessness** |  |  |  |  |  |  |  |  |  |  |  |
| Currently homeless | 16.1 | (0.1) |  | 2.8 | (0.0) |  | 19.0 | (0.1) |  | 3.6 | (0.0) |
| Homeless in the past 12 months but not currently | 19.5 | (0.1) |  | 5.9 | (0.0) |  | 22.1 | (0.1) |  | 6.7 | (0.0) |
| Not homeless in the past 12 months | 64.4 | (0.1) |  | 91.3 | (0.0) |  | 58.9 | (0.1) |  | 89.7 | (0.0) |
| **Era of service** |  |  |  |  |  |  |  |  |  |  |  |
| Pre-Vietnam | 5.7 | (0.0) |  | 21.8 | (0.0) |  | 3.6 | (0.1) |  | 18.4 | (0.0) |
| Vietnam | 39.4 | (0.1) |  | 48.3 | (0.0) |  | 37.1 | (0.1) |  | 47.8 | (0.0) |
| Post-Vietnam | 25.1 | (0.1) |  | 12.0 | (0.0) |  | 22.9 | (0.1) |  | 13.4 | (0.0) |
| Persian Gulf War | 29.9 | (0.1) |  | 18.0 | (0.0) |  | 36.3 | (0.1) |  | 20.5 | (0.0) |
| **High risk of suicide** |  |  |  |  |  |  |  |  |  |  |  |
| **Supplementary Table 11 continued. Sample distribution on stratification variables** | | | | | | | | | | | |
|  | | | | | | | | | | | |
|  | **Training sample**  **(January 1, 2010 - October 22, 2012)** | | | | |  | **Holdout sample**  **(October 23, 2012 - December 31, 2013)** | | | | |
|  | **Psychiatric hospitalizations**  **(n = 273,860)** | |  | **All VHA visits**  **(n = 196,986,095)** | |  | **Psychiatric hospitalizations**  **(n = 117,158)** | |  | **All VHA visits**  **(n = 89,109,223)** | |
|  | **%** | **(SE)** |  | **%** | **(SE)** |  | **%** | **(SE)** |  | **%** | **(SE)** |
| Yes | 15.1 | (0.1) |  | 0.8 | (0.0) |  | 8.3 | (0.1) |  | 0.5 | (0.0) |
| No | 84.9 | (0.1) |  | 99.2 | (0.0) |  | 91.7 | (0.1) |  | 99.5 | (0.0) |
|  | | | | | | | | | | | |

Abbreviations. VHA = Veterans Health Administration; SE = standard error.

| **Supplementary Table 12. Stratification variable models estimated in the training sample (January 1, 2010-October 22, 2012) to predict post-discharge suicides over 5 time horizons** | | | | | | | | | | | | | | |
| --- | --- | --- | --- | --- | --- | --- | --- | --- | --- | --- | --- | --- | --- | --- |
|  | | | | | | | | | | | | | | |
|  | **Time horizon for prediction in the training sample** | | | | | | | | | | | | | |
|  | **1-week** | |  | **1-month** | |  | **3-months** | |  | **6-months** | |  | **12-months** | |
|  | **OR** | **(95% CI)** |  | **OR** | **(95% CI)** |  | **OR** | **(95% CI)** |  | **OR** | **(95% CI)** |  | **OR** | **(95% CI)** |
| **Age** |  |  |  |  |  |  |  |  |  |  |  |  |  |  |
| 20-40 | 0.6 | (0.2-2.0) |  | 0.4* | (0.2-0.8) |  | 0.6* | (0.4-1.0) |  | 0.7* | (0.4-1.0) |  | 0.7* | (0.5-1.0) |
| 41-50 | 0.8 | (0.3-1.9) |  | 0.5* | (0.3-0.9) |  | 0.8 | (0.5-1.2) |  | 0.8 | (0.6-1.2) |  | 0.8 | (0.6-1.1) |
| 51-55 | 1.0 | Ref |  | 1.0 | Ref |  | 1.0 | Ref |  | 1.0 | Ref |  | 1.0 | Ref |
| 56-61 | 1.4 | (0.4-4.2) |  | 0.6 | (0.3-1.2) |  | 0.8 | (0.5-1.3) |  | 0.8 | (0.6-1.1) |  | 0.7* | (0.5-0.9) |
| 62-98 | 1.6 | (0.4-6.1) |  | 0.9 | (0.5-1.9) |  | 0.8 | (0.5-1.3) |  | 0.7 | (0.5-1.0) |  | 0.7* | (0.5-0.9) |
| χ^2^_4_ | 1.5 | p=.83 |  | 9.6* | p=.048 |  | 5.7 | p=.23 |  | 7.2 | p=.13 |  | 11.1* | p=.026 |
| **Sex** |  |  |  |  |  |  |  |  |  |  |  |  |  |  |
| Female | 1.0 | Ref |  | 1.0 | Ref |  | 1.0 | Ref |  | 1.0 | Ref |  | 1.0 | Ref |
| Male | 2.0 | (0.6-6.5) |  | 3.3* | (1.3-8.1) |  | 2.2* | (1.3-3.8) |  | 1.8* | (1.2-2.6) |  | 1.3 | (1.0-1.7) |
| χ^2^_1_ | 1.3 | p=.26 |  | 6.7* | p=.010 |  | 8.7* | p=.003 |  | 8.8* | p=.003 |  | 3.4 | p=.06 |
| **Race/ethnicity** |  |  |  |  |  |  |  |  |  |  |  |  |  |  |
| Non-Hispanic White | 4.2* | (1.3-13.9) |  | 3.0* | (1.6-5.6) |  | 2.8* | (1.8-4.3) |  | 2.4* | (1.8-3.4) |  | 2.8* | (2.2-3.7) |
| Non-Hispanic Black | 1.0 | Ref |  | 1.0 | Ref |  | 1.0 | Ref |  | 1.0 | Ref |  | 1.0 | Ref |
| Hispanic | 0.4 | (0.0-4.0) |  | 1.0 | (0.4-2.5) |  | 1.4 | (0.8-2.6) |  | 1.3 | (0.8-2.0) |  | 1.2 | (0.8-1.8) |
| Other (including missing) | 5.3* | (1.3-22.0) |  | 4.5* | (2.1-9.7) |  | 4.2* | (2.4-7.2) |  | 3.5* | (2.3-5.3) |  | 3.4* | (2.4-4.8) |
| χ^2^_3_ | 11.3* | p=.010 |  | 23.1* | p<.001 |  | 35.6* | p<.001 |  | 49.4* | p<.001 |  | 89.8* | p<.001 |
| **Marital status** |  |  |  |  |  |  |  |  |  |  |  |  |  |  |
| Married | 0.8 | (0.3-2.3) |  | 1.1 | (0.6-2.3) |  | 1.3 | (0.8-2.2) |  | 1.6* | (1.0-2.4) |  | 1.7* | (1.2-2.5) |
| Divorced | 0.6 | (0.2-1.7) |  | 1.1 | (0.6-2.3) |  | 1.2 | (0.7-1.9) |  | 1.3 | (0.9-2.1) |  | 1.5* | (1.1-2.1) |
| Separated | 1.0 | Ref |  | 1.0 | Ref |  | 1.0 | Ref |  | 1.0 | Ref |  | 1.0 | Ref |
| Widowed | 0.0 | (0.0->999.9) |  | 0.6 | (0.2-2.4) |  | 0.7 | (0.3-1.8) |  | 1.0 | (0.5-2.0) |  | 1.4 | (0.9-2.4) |
| Never married | 1.5 | (0.6-4.1) |  | 2.1* | (1.0-4.2) |  | 2.0* | (1.2-3.2) |  | 2.5* | (1.6-3.8) |  | 2.9* | (2.0-4.1) |
| χ^2^_4_ | 7.3 | p=.12 |  | 13.7* | p=.009 |  | 18.6* | p=.002 |  | 40.5* | p<.001 |  | 75.3* | p<.001 |
| **Income** |  |  |  |  |  |  |  |  |  |  |  |  |  |  |
| No income | 1.3 | (0.5-3.1) |  | 1.5 | (0.9-2.4) |  | 1.4 | (1.0-2.0) |  | 1.3 | (1.0-1.7) |  | 1.5* | (1.2-1.9) |
| Low | 1.9 | (0.8-4.4) |  | 1.5 | (0.9-2.5) |  | 1.4 | (1.0-2.0) |  | 1.5* | (1.1-2.0) |  | 1.4* | (1.1-1.8) |
| Low-average | 1.0 | Ref |  | 1.0 | Ref |  | 1.0 | Ref |  | 1.0 | Ref |  | 1.0 | Ref |
| High-average | 1.9 | (0.8-4.4) |  | 1.8* | (1.1-3.0) |  | 1.5* | (1.1-2.1) |  | 1.5* | (1.1-1.9) |  | 1.7 * | (1.4-2.1) |

| **Supplementary Table 12 continued. Stratification variable models estimated in the training sample (January 1, 2010-October 22, 2012) to predict post-discharge suicides over 5 time horizons** | | | | | | | | | | | | | | |
| --- | --- | --- | --- | --- | --- | --- | --- | --- | --- | --- | --- | --- | --- | --- |
|  | | | | | | | | | | | | | | |
|  | **Time horizon for prediction in the training sample** | | | | | | | | | | | | | |
|  | **1-week** | |  | **1-month** | |  | **3-months** | |  | **6-months** | |  | **12-months** | |
|  | **OR** | **(95% CI)** |  | **OR** | **(95% CI)** |  | **OR** | **(95% CI)** |  | **OR** | **(95% CI)** |  | **OR** | **(95% CI)** |
| High | 1.4 | (0.6-3.2) |  | 1.4 | (0.9-2.3) |  | 1.3 | (0.9-1.7) |  | 1.3* | (1.0-1.7) |  | 1.5* | (1.2-1.8) |
| χ^2^_4_ | 3.0 | p=.56 |  | 6.1 | p=.19 |  | 6.4 | p=.17 |  | 10.2* | p=0.38 |  | 26.0* | p<.001 |
| **Religion** |  |  |  |  |  |  |  |  |  |  |  |  |  |  |
| Baptist |  |  |  |  |  |  |  |  |  |  |  |  |  |  |
| Black Baptist | 0.0 | (0.0->999.9) |  | 0.3 | (0.1-1.2) |  | 0.2* | (0.0-0.5) |  | 0.2* | (0.1-0.4) |  | 0.2* | (0.1-0.4) |
| Evangelical and other Baptist | 0.2* | (0.1-0.5) |  | 0.4* | (0.2-0.6) |  | 0.4* | (0.3-0.6) |  | 0.5* | (0.3-0.6) |  | 0.6* | (0.4-0.7) |
| Other Protestant | 1.0 | Ref |  | 1.0 | Ref |  | 1.0 | Ref |  | 1.0 | Ref |  | 1.0 | Ref |
| Roman Catholic | 0.8 | (0.4-1.5) |  | 0.7 | (0.4-1.0) |  | 0.6 | (0.4-0.8) |  | 0.6* | (0.4-0.7) |  | 0.6* | (0.5-0.7) |
| Other Christian (including Jehovah's Witnesses, Latter Day Saints, non-Specific Christian, Orthodox) | 0.1* | (0.0-0.9) |  | 0.7 | (0.4-1.4) |  | 0.8 | (0.5-1.2) |  | 0.7 | (0.5-1.0) |  | 0.8 | (0.6-1.0) |
| Other non-Christian (including Buddhism, Hindu, Islam, Jewish) | 0.3 | (0.1-1.4) |  | 0.6 | (0.3-1.2) |  | 0.5 | (0.3-0.8) |  | 0.7 | (0.5-1.0) |  | 0.6* | (0.5-0.9) |
| None | 0.6 | (0.3-1.1) |  | 0.7 | (0.5-1.1) |  | 0.8 | (0.6-1.1) |  | 0.8* | (0.6-1.0) |  | 0.7* | (0.6-0.8) |
| χ^2^_6_ | 15.2* | p=.019 |  | 15.7* | p=.015 |  | 35.0* | p<.001 |  | 50.2* | p<.001 |  | 58.3* | p<.001 |
| **Census region** |  |  |  |  |  |  |  |  |  |  |  |  |  |  |
| Northeast (including Puerto Rico & US Virgin Islands) | 1.0 | Ref |  | 1.0 | Ref |  | 1.0 | Ref |  | 1.0 | Ref |  | 1.0 | Ref |
| Midwest | 1.0 | (0.4-2.3) |  | 1.6 | (1.0-2.6) |  | 1.6* | (1.1-2.3) |  | 1.8* | (1.3-2.4) |  | 1.6* | (1.3-2.0) |
| South | 1.5 | (0.7-3.2) |  | 1.4 | (0.9-2.3) |  | 1.5* | (1.1-2.1) |  | 1.7* | (1.3-2.3) |  | 1.6* | (1.3-2.0) |
| West (including American Samoa, Guam, Northern Mariana Islands) | 1.2 | (0.5-2.7) |  | 1.3 | (0.7-2.1) |  | 1.2 | (0.8-1.8) |  | 1.4* | (1.0-1.9) |  | 1.4* | (1.1-1.8) |
| χ^2^_3_ | 1.9 | p=.58 |  | 3.5 | p=.33 |  | 7.7 | p=.05 |  | 19.1* | p<.001 |  | 20.0* | p<.001 |
| **Urbanicity** |  |  |  |  |  |  |  |  |  |  |  |  |  |  |
| Metro area with >1m population | 1.0 | Ref |  | 1.0 | Ref |  | 1.0 | Ref |  | 1.0 | Ref |  | 1.0 | Ref |
| Metro area with 250k - 1m population | 0.9 | (0.4-1.8) |  | 1.0 | (0.7-1.5) |  | 1.0 | (0.8-1.3) |  | 1.0 | (0.8-1.3) |  | 1.1 | (1.0-1.3) |
| Metro area with <250k population | 2.1* | (1.0-4.4) |  | 1.7* | (1.1-2.7) |  | 1.3 | (0.9-1.8) |  | 1.2 | (0.9-1.5) |  | 1.1 | (0.9-1.4) |
| Urban area with ≥20k population | 1.0 | (0.4-3.0) |  | 1.0 | (0.5-1.8) |  | 0.7 | (0.4-1.2) |  | 0.8 | (0.5-1.1) |  | 0.8 | (0.6-1.1) |
| Urban/rural area with <20k population | 0.9 | (0.4-2.2) |  | 0.9 | (0.5-1.5) |  | 0.9 | (0.6-1.2) |  | 0.9 | (0.7-1.2) |  | 0.9 | (0.7-1.1) |
| χ^2^_4_ | 5.7 | p=.23 |  | 7.7 | p=.10 |  | 5.8 | p=.21 |  | 4.0 | p=.40 |  | 7.3 | p=.12 |
| **Homelessness** |  |  |  |  |  |  |  |  |  |  |  |  |  |  |
| Currently homeless | 1.0 | (0.5-2.1) |  | 0.7 | (0.4-1.1) |  | 0.7* | (0.5-1.0) |  | 0.6* | (0.4-0.8) |  | 0.7* | (0.5-0.9) |
| Homeless in the past 12 months but not currently | 0.8 | (0.4-1.8) |  | 0.7 | (0.5-1.2) |  | 0.7* | (0.5-1.0) |  | 0.7* | (0.5-0.9) |  | 0.9 | (0.7-1.0) |

| **Supplementary Table 12 continued. Stratification variable models estimated in the training sample (January 1, 2010-October 22, 2012) to predict post-discharge suicides over 5 time horizons** | | | | | | | | | | | | | | |
| --- | --- | --- | --- | --- | --- | --- | --- | --- | --- | --- | --- | --- | --- | --- |
|  | | | | | | | | | | | | | | |
|  | **Time horizon for prediction in the training sample** | | | | | | | | | | | | | |
|  | **1-week** | |  | **1-month** | |  | **3-months** | |  | **6-months** | |  | **12-months** | |
|  | **OR** | **(95% CI)** |  | **OR** | **(95% CI)** |  | **OR** | **(95% CI)** |  | **OR** | **(95% CI)** |  | **OR** | **(95% CI)** |
| Not homeless in the past 12 months | 1.0 | Ref |  | 1.0 | Ref |  | 1.0 | Ref |  | 1.0 | Ref |  | 1.0 | Ref |
| χ^2^_2_ | 0.2 | p=.89 |  | 3.3 | p=.20 |  | 7.1* | p=.029 |  | 17.6* | p<.001 |  | 11.6* | p=.003 |
| **Era of service** |  |  |  |  |  |  |  |  |  |  |  |  |  |  |
| Pre-Vietnam | 0.3 | (0.0-1.7) |  | 1.0 | (0.4-2.5) |  | 1.7 | (0.9-3.4) |  | 1.6 | (0.9-2.8) |  | 1.9* | (1.2-3.0) |
| Vietnam | 0.3* | (0.1-0.9) |  | 0.9 | (0.5-1.8) |  | 1.2 | (0.8-2.0) |  | 1.5* | (1.0-2.2) |  | 1.8* | (1.3-2.5) |
| Post-Vietnam | 1.0 | Ref |  | 1.0 | Ref |  | 1.0 | Ref |  | 1.0 | Ref |  | 1.0 | Ref |
| Persian Gulf War | 1.3 | (0.5-3.3) |  | 2.6* | (1.5-4.7) |  | 2.3* | (1.5-3.4) |  | 2.0* | (1.4-2.8) |  | 2.1* | (1.6-2.8) |
| χ^2^_3_ | 5.6 | p=.13 |  | 11.8* | p=.008 |  | 16.4* | p<.001 |  | 17.8* | p<.001 |  | 36.5* | p<.001 |
| **High risk of suicide** |  |  |  |  |  |  |  |  |  |  |  |  |  |  |
| Yes | 2.5* | (1.4-4.5) |  | 1.8* | (1.2-2.6) |  | 2.2* | (1.7-2.8) |  | 2.2* | (1.8-2.6) |  | 2.6* | (2.3-3.0) |
| No | 1.0 | Ref |  | 1.0 | Ref |  | 1.0 | Ref |  | 1.0 | Ref |  | 1.0 | Ref |
| χ^2^_1_ | 9.3* | p=.002 |  | 8.7* | p=.003 |  | 36.4* | p<.001 |  | 57.1* | p<.001 |  | 155.8* | p<.001 |
| χ^2^_35_ | 59.8* | p=.006 |  | 116.8* | p<.001 |  | 206.7* | p<.001 |  | 305.0* | p<.001 |  | 535.6* | p<.001 |
|  | | | | | | | | | | | | | | |

*Significant at .05 level, two sided test.

Abbreviations. OR = odds ratio; CI = confidence interval.

| **Supplementary Table 13. Super Learner classifier weights in each of the 5 ensembles** | | | | | | | | | | | | | | | | | |
| --- | --- | --- | --- | --- | --- | --- | --- | --- | --- | --- | --- | --- | --- | --- | --- | --- | --- |
|  | | | | | | | | | | | | | | | | | |
|  |  |  |  | **Super learner importance rankings and weights for the different time horizons** | | | | | | | | | | | | | |
|  |  |  |  | **1-week** | |  | **1-month** | |  | **3-month** | |  | **6-month** | |  | **12-month** | |
| **Classifier^1^** |  | **FS Method^2^** |  | **Rank** | **Weight** |  | **Rank** | **Weight** |  | **Rank** | **Weight** |  | **Rank** | **Weight** |  | **Rank** | **Weight** |
| Logistic regression |  | P value |  | 8 | .057 |  | 10 | .024 |  | 17 | .000 |  | 17 | .000 |  | 17 | .000 |
|  |  | LASSO |  | 3 | .109 |  | 5 | .078 |  | 2 | .059 |  | 5 | .021 |  | 6 | .012 |
| Elastic net regularization |  | P value |  | 5 | .079 |  | 9 | .025 |  | 17 | .000 |  | 17 | .000 |  | 17 | .000 |
|  |  | LASSO |  | 11 | .048 |  | 17 | .000 |  | 6 | .008 |  | 17 | .000 |  | 7 | .005 |
| Random forest decision trees |  | P value |  | 2 | .169 |  | 1 | .391 |  | 1 | .811 |  | 1 | .493 |  | 1 | .509 |
|  |  | LASSO |  | 17 | .000 |  | 2 | .136 |  | 17 | .000 |  | 17 | .000 |  | 17 | .000 |
| Support vector machines linear kernel |  | P value |  | 9 | .055 |  | 17 | .000 |  | 3 | .051 |  | 17 | .000 |  | 17 | .000 |
|  |  | LASSO |  | 10 | .049 |  | 12 | .000 |  | 17 | .000 |  | 6 | .000 |  | 17 | .000 |
| Support vector machines radial kernel |  | P value |  | 17 | .000 |  | 4 | .090 |  | 17 | .000 |  | 17 | .000 |  | 5 | .019 |
|  |  | LASSO |  | 7 | .062 |  | 17 | .000 |  | 17 | .000 |  | 4 | .074 |  | 4 | .101 |
| Support vector machines polynomial kernel |  | P value |  | 6 | .067 |  | 7 | .033 |  | 17 | .000 |  | 17 | .000 |  | 17 | .000 |
|  |  | LASSO |  | 17 | .000 |  | 17 | .000 |  | 17 | .000 |  | 7 | .000 |  | 17 | .000 |
| Bayesian additive regression trees |  | P value |  | 17 | .000 |  | 17 | .000 |  | 17 | .000 |  | 17 | .000 |  | 17 | .000 |
|  |  | LASSO |  | 17 | .000 |  | 6 | .073 |  | 5 | .023 |  | 17 | .000 |  | 17 | .000 |
| Extreme gradient boosting |  | P value |  | 4 | .093 |  | 3 | .117 |  | 7 | .000 |  | 2 | .255 |  | 2 | .205 |
|  |  | LASSO |  | 12 | .003 |  | 8 | .031 |  | 4 | .047 |  | 3 | .158 |  | 3 | .148 |
| Neural Networks |  | RF |  | 1 | .210 |  | 11 | .000 |  | 17 | .000 |  | 17 | .000 |  | 8 | .000 |
|  |  |  |  |  |  |  |  |  |  |  |  |  |  |  |  |  |  |

^1^GLM = Generalized linear model; RF = Random forest; SVM = Support Vector Machine with either a linear, radial, or polynomial kernel; BART = Bayesian Additive Regression Trees; XGBoost = Extreme Gradient Boosting; NN= Neural Network.

^2”^FS Method” = feature selection method. As noted in the text, we used three different feature selection methods to reduce the number of potential predictors included in Super learner: (i) PV = univariate p value less than .10 controlling for the stratification variables and, within this set; (ii) LASSO and (iii) RF = Random Forest.

**References**

1. Bostwick JM, Pabbati C, Geske JR, McKean AJ. Suicide attempt as a risk factor for completed suicide: even more lethal than we knew. Am J Psychiatry (2016) 173:1094-100. doi:10.1176/appi.ajp.2016.15070854

2. Simon GE, Johnson E, Lawrence JM, Rossom RC, Ahmedani B, Lynch FL, et al. Predicting suicide attempts and suicide deaths following outpatient visits using electronic health records. Am J Psychiatry (2018) 175:951-60. doi:10.1176/appi.ajp.2018.17101167

3. Troister T, Links PS, Cutcliffe J. Review of predictors of suicide within 1 year of discharge from a psychiatric hospital. Curr Psychiatry Rep (2008) 10:60-5. doi:10.1007/s11920-008-0011-8

4. Berg JM, Malte CA, Reger MA, Hawkins EJ. Medical records flag for suicide risk: predictors and subsequent use of care among veterans with substance use disorders. Psychiatr Serv (2018) 69:993-1000. doi:10.1176/appi.ps.201700545

5. US Department of Veterans Affairs. Patient record flags (PRF) user guide (2019). https://www.va.gov/vdl/documents/Clinical/Patient_Record_Flags/patient_record_flags_user_guide.pdf [Accessed October 15, 2019].

6. Centers for Disease Control and Prevention. International Classification of Diseases, Ninth Revision, Clinical Modification (ICD-9-CM) (2013). http://www.cdc.gov/nchs/icd/icd9cm.htm [Accessed October 11, 2019].

7. Bachmann S. Epidemiology of suicide and the psychiatric perspective. Int J Environ Res Public Health (2018) 15:E1425. doi:10.3390/ijerph15071425

8. Kessler RC, Stein MB, Petukhova MV, Bliese P, Bossarte RM, Bromet EJ, et al. Predicting suicides after outpatient mental health visits in the Army Study to Assess Risk and Resilience in Servicemembers (Army STARRS). Mol Psychiatry (2017) 22:544-51. doi:10.1038/mp.2016.110

9. Forehand JA, Peltzman T, Westgate CL, Riblet NB, Watts BV, Shiner B. Causes of excess mortality in veterans treated for posttraumatic stress disorder. Am J Prev Med (2019) 57:145-52. doi:10.1016/j.amepre.2019.03.014

10. Elixhauser A, Steiner C, Palmer L. Clinical Classifications Software (CCS). US Agency for Healthcare Research and Quality (2015). http://www.hcup-us.ahrq.gov/toolssoftware/ccs/ccs.jsp [Accessed October 30, 2019].

11. Owen-Smith AA, Ahmedani BK, Peterson E, Simon GE, Rossom RC, Lynch FL, et al. The mediating effect of sleep disturbance on the relationship between nonmalignant chronic pain and suicide death. Pain Pract (2019) 19:382-9. doi:10.1111/papr.12750

12. Ahmedani BK, Peterson EL, Hu Y, Rossom RC, Lynch F, Lu CY, et al. Major physical health conditions and risk of suicide. Am J Prev Med (2017) 53:308-15. doi:10.1016/j.amepre.2017.04.001

13. Breslau N. Migraine, suicidal ideation, and suicide attempts. Neurology (1992) 42:392-5.

14. Calandre EP, Vilchez JS, Molina-Barea R, Tovar MI, Garcia-Leiva JM, Hidalgo J, et al. Suicide attempts and risk of suicide in patients with fibromyalgia: a survey in Spanish patients. Rheumatology (2011) 50:1889-93. doi:10.1093/rheumatology/ker203

15. Cheatle MD. Depression, chronic pain, and suicide by overdose: on the edge. Pain Med (2011) 12:S43-S8. doi:10.1111/j.1526-4637.2011.01131.x

16. Ilgen MA, Kleinberg F, Ignacio RV, Bohnert ASB, Valenstein M, McCarthy JF, et al. Noncancer pain conditions and risk of suicide. JAMA Psychiatry (2013) 70:692-7. doi:10.1001/jamapsychiatry.2013.908

17. Racine M. Chronic pain and suicide risk: a comprehensive review. Prog Neuropsychopharmacol Biol Psychiatry (2018) 87:269-80. doi:10.1016/j.pnpbp.2017.08.020

18. Raphael KG, Janal MN, Nayak S, Schwartz JE, Gallagher RM. Psychiatric comorbidities in a community sample of women with fibromyalgia. Pain (2006) 124:117-25. doi:10.1016/j.pain.2006.04.004

19. Wolfe F, Hassett AL, Walitt B, Michaud K. Mortality in fibromyalgia: a study of 8,186 patients over thirty-five years. Arthritis Care Res (2011) 63:94-101. doi:10.1002/acr.20301

20. Cifu DX, Taylor BC, Carne WF, Bidelspach D, Sayer NA, Scholten J, et al. Traumatic brain injury, posttraumatic stress disorder, and pain diagnoses in OIF/OEF/OND Veterans. J Rehabil Res Dev (2013) 50:1169-76. doi:10.1682/JRRD.2013.01.0006

21. Ilgen MA, Zivin K, Austin KL, Bohnert ASB, Czyz EK, Valenstein M, et al. Severe pain predicts greater likelihood of subsequent suicide. Suicide Life Threat Behav (2010) 40:597-608. doi:10.1521/suli.2010.40.6.597

22. Finley EP, Bollinger M, Noël PH, Amuan ME, Copeland LA, Pugh JA, et al. A national cohort study of the association between the polytrauma clinical triad and suicide-related behavior among US veterans who served in Iraq and Afghanistan. Am J Public Health (2015) 105:380-7. doi:10.2105/AJPH.2014.301957

23. Pugh MJV, Finley EP, Copeland LA, Wang C-P, Noel PH, Amuan ME, et al. Complex comorbidity clusters in OEF/OIF Veterans: the polytrauma clinical triad and beyond. Med Care (2014) 52:172-81. doi:10.1097/MLR.0000000000000059

24. Bryan CJ, Clemans TA. Repetitive traumatic brain injury, psychological symptoms, and suicide risk in a clinical sample of deployed military personnel. JAMA Psychiatry (2013) 70:686-91. doi:10.1001/jamapsychiatry.2013.1093

25. Kennedy HG, Iveson RCY, Hill O. Violence, homicide and suicide: strong correlation and wide variation across districts. Br J Psychiatry (1999) 175:462-6. doi:10.1192/bjp.175.5.462

26. Chung DT, Ryan CJ, Hadzi-Pavlovic D, Singh SP, Stanton C, Large MM. Suicide rates after discharge from psychiatric facilities: a systematic review and meta-analysis. JAMA Psychiatry (2017) 74:694-702. doi:10.1001/jamapsychiatry.2017.1044

27. Winkler P, Mladá K, Csémy L, Nechanská B, Höschl C. Suicides following inpatient psychiatric hospitalization: a nationwide case control study. J Affect Disord (2015) 184:164-9. doi:10.1016/j.jad.2015.05.039

28. Luxton DD, Thomas EK, Chipps J, Relova RM, Brown D, McLay R, et al. Caring letters for suicide prevention: implementation of a multi-site randomized clinical trial in the U.S. military and veteran affairs healthcare systems. Contemp Clin Trials (2014) 37:252-60. doi:10.1016/j.cct.2014.01.007

29. Swaraj S, Chung D, Curtis J, Firth J, Ramanuj PP, Sara G, et al. Meta‐analysis of natural, unnatural, and cause‐specific mortality rates following discharge from inpatient psychiatric facilities. Acta Psychiatr Scand (2019) 140:244-64. doi:10.1111/acps.13073

30. Forte A, Buscajoni A, Fiorillo A, Pompili M, Baldessarini RJ. Suicidal risk following hospital discharge: a review. Harv Rev Psychiatry (2019) 27:209-16. doi:10.1097/HRP.0000000000000222

31. Forsman J, Taipale H, Masterman T, Tiihonen J, Tanskanen A. Adherence to psychotropic medication in completed suicide in Sweden 2006–2013: a forensic-toxicological matched case-control study. Eur J Clin Pharmacol (2019) 75:1421-30. doi:10.1007/s00228-019-02707-z

32. O'Neill S, Graham B, Ennis E. Prescribed pain and mental health medication prior to suicide: a population based case control study. J Affect Disord (2019) 246:195-200. doi:10.1016/j.jad.2018.12.018

33. Reneflot A, Kaspersen SL, Hauge LJ, Kalseth J. Use of prescription medication prior to suicide in Norway. BMC Health Serv Res (2019) 19:215. doi:10.1186/s12913-019-4009-1

34. Windfuhr K, While D, Kapur N, Ashcroft DM, Kontopantelis E, Carr MJ, et al. Suicide risk linked with clinical consultation frequency, psychiatric diagnoses and psychotropic medication prescribing in a national study of primary-care patients. Psychol Med (2016) 46:3407-17. doi:10.1017/S0033291716001823

35. US Department of Veterans Affairs. VA National Formulary - Pharmacy Benefits Management Services (2019). https://www.pbm.va.gov/nationalformulary.asp [Accessed October 1, 2019].

36. Pfeifer P, Greusing S, Kupferschmidt H, Bartsch C, Reisch T. A comprehensive analysis of attempted and fatal suicide cases involving frequently used psychotropic medications. Gen Hosp Psychiatry (2019) [epub ahead of print]. doi:10.1016/j.genhosppsych.2019.07.011

37. US Food & Drug Administration. FDALabel: Full-text search of drug labeling (2019). https://www.fda.gov/science-research/bioinformatics-tools/fdalabel-full-text-search-drug-labeling [Accesed October 16, 2019].

38. Meltzer HY. Clozapine treatment for suicidality in schizophrenia: International Suicide Prevention Trial (InterSePT). Arch Gen Psychiatry (2003) 60:82-91. doi:10.1001/archpsyc.60.1.82

39. Pompili M, Baldessarini RJ, Forte A, Erbuto D, Serafini G, Fiorillo A, et al. Do atypical antipsychotics have antisuicidal effects? a hypothesis-generating overview. Int J Mol Sci (2016) 17:E1700.. doi:10.3390/ijms17101700

40. Pompili M, Orsolini L, Lamis DA, Goldsmith DR, Nardella A, Falcone G, et al. Suicide prevention in schizophrenia: do long-acting injectable antipsychotics (LAIs) have a role? CNS Neurol Disord Drug Targets (2017) 16:454-62. doi:10.2174/1871527316666170223163629

41. Whyte A, Parker C. A review of the efficacy and tolerability of antipsychotic long-acting injections. Prog Neurol Psychiatry (2016) 20:22-8. doi:10.1002/pnp.436

42. Rainer MK. Risperidone long-acting injection: a review of its long term safety and efficacy. Neuropsychiatr Dis Treat (2008) 4:919-27.

43. Schneider-Thoma J, Efthimiou O, Huhn M, Krause M, Reichelt L, Röder H, et al. Second-generation antipsychotic drugs and short-term mortality: a systematic review and meta-analysis of placebo-controlled randomised controlled trials. Lancet Psychiatry (2018) 5:653-63. doi:10.1016/S2215-0366(18)30177-9

44. Altamura AC, Sassella F, Santini A, Montresor C, Fumagalli S, Mundo E. Intramuscular preparations of antipsychotics: uses and relevance in clinical practice. Drugs (2003) 63:493-512. doi:10.2165/00003495-200363050-00004

45. Caley CF, Perriello E, Golden J. Antiepileptic drugs and suicide-related outcomes in bipolar disorder: a descriptive review of published data. Ment Health Clin (2018) 8:138-47. doi:10.9740/mhc.2018.05.138

46. Arana A, Wentworth CE, Ayuso-Mateos JL, Arellano FM. Suicide-related events in patients treated with antiepileptic drugs. N Engl J M ed (2010) 363:542-51.

47. Abou-Saleh MT, Müller-Oerlinghausen B, Coppen AJ. Lithium in the episode and suicide prophylaxis and in augmenting strategies in patients with unipolar depression. Int J Bipolar Disord (2017) 5:11. doi:10.1186/s40345-017-0080-x

48. Hafeman DM, Rooks B, Merranko J, Liao F, Gill MK, Goldstein TR, et al. Lithium versus other mood stabilizing medications in a longitudinal study of bipolar youth. J Am Acad Child Adolesc Psychiatry (2019) [epub ahead of print]. doi:10.1016/j.jaac.2019.06.013

49. Blüml V, Regier MD, Hlavin G, Rockett IRH, König F, Vyssoki B, et al. Lithium in the public water supply and suicide mortality in Texas. J Psychiatr Res (2013) 47:407-11. doi:10.1016/j.jpsychires.2012.12.002

50. Kapusta ND, Mossaheb N, Etzersdorfer E, Hlavin G, Thau K, Willeit M, et al. Lithium in drinking water and suicide mortality. Br J of Psychiatry (2011) 198:346-50. doi:10.1192/bjp.bp.110.091041

51. Hesdorffer DC, Berg AT, Kanner AM. An update on antiepileptic drugs and suicide: are there definitive answers yet? Epilepsy Curr (2010) 10:137-45. doi:10.1111/j.1535-7511.2010.01382.x

52. Saltz BL, Robinson DG, Woerner MG. Recognizing and managing antipsychotic drug treatment side effects in the elderly. Prim Care Companion J Clin Psychiatry (2004) 6:14–19.

53. Stroup TS, Gray N. (2018). Management of common adverse effects of antipsychotic medications. World Psychiatry (2018) 17:341–56. doi: 10.1002/wps.20567

54. Seemüller F, Lewitzka U, Bauer M, Meyer S, Musil R, Schennach R, et al. The relationship of akathisia with treatment emergent suicidality among patients with first-episode schizophrenia treated with haloperidol or risperidone. Pharmacopsychiatry (2012) 45:292-96. doi: 10.1055/s-0032-1309004

55. Blosnich JR, Montgomery AE, Dichter ME, Gordon AJ, Kavalieratos D, Taylor L, et al. Social determinants and military veterans’ suicide ideation and attempt: a cross-sectional analysis of electronic health record data. J Gen Intern Med (2019) [epub ahead of print]. doi:10.1007/s11606-019-05447-z

56. Dobscha SK, Denneson LM, Kovas AE, Teo A, Forsberg CW, Kaplan MS, et al. Correlates of suicide among veterans treated in primary care: case-control study of a nationally representative sample. J Gen Intern Med (2014) 29:853-60. doi:10.1007/s11606-014-3028-1

57. Chen T, Roberts K. Negative life events and suicide in the National Violent Death Reporting System. Arch of Suicide Res (2019) [epub ahead of print]. doi:10.1080/13811118.2019.1677275

58. Torres JM, Lawlor J, Colvin JD, Sills MR, Bettenhausen JL, Davidson A, et al. ICD social codes: an underutilized resource for tracking social needs. Med Care (2017) 55:810-6. doi:10.1097/MLR.0000000000000764

59. Butterfield RC, Gottschalk M, LaBrec PA, Health M. Development of an individual level social determinants of health (SDoH) Model (2018). https://pdfs.semanticscholar.org/6c31/2be712e72b48d6fda3ee218826cc99c4c033.pdf [Accessed October 29, 2019].

60. Ajdadic-Gross V, Ring M, Gadola E, Lauber C, Bopp M, Gutzwiller F, et al. Suicide after bereavement: an overlooked problem. Psychol Med (2008) 38:673-6.

61. Tsai J, Cao X. Association between suicide attempts and homelessness in a population-based sample of US veterans and non-veterans. J Epidemiol Community Health (2019) 73:346-52. doi:10.1136/jech-2018-211065

62. Dworkin ER, Menon SV, Bystrynski J, Allen NE. Sexual assault victimization and psychopathology: a review and meta-analysis. Clin Psychol Rev (2017) 56:65-81. doi:10.1016/j.cpr.2017.06.002

63. Giesbrecht N, Huguet N, Ogden L, Kaplan MS, McFarland BH, Caetano R, et al. Acute alcohol use among suicide decedents in 14 US states: impacts of off‐premise and on‐premise alcohol outlet density. Addiction (2015) 110:300-7. doi:https://doi.org/10.1111/add.12762

64. Johnson FW, Gruenewald PJ, Remer LG. Suicide and alcohol: do outlets play a role? Alcohol Clin Exp Res (2009) 33:2124-33. doi:https://doi.org/10.1111/j.1530-0277.2009.01052.x

65. Infogroup. Infogroup US Historical Business Data, Harvard Dataverse, V9 (2016). doi:10.7910/DVN/PNOFKI

66. Gururaj G, Isaac MK, Subbakrishna DK, Ranjani R. Risk factors for completed suicides: a case–control study from Bangalore, India. Inj Control Saf Promot (2004) 11:183-91. doi:10.1080/156609704/233/289706

67. Kidger J, Gunnell D, Jarvik JG, Overstreet KA, Hollingworth W. The association between bankruptcy and hospital-presenting attempted suicide: a record linkage study. Suicide Life Threat Behav (2011) 41:676-84. doi:10.1111/j.1943-278X.2011.00063.x

68. Public Access to Court Electronic Records (PACER). Bankruptcy Statistics, Report F-5A (2019). http://www.uscourts.gov/Statistics/BankruptcyStatistics.aspx [Accessed December 8, 2019].

69. US Census Bureau. Population estimates (2018). https://www2.census.gov/programs-surveys/popest/datasets/ [Accessed October 28, 2019].

70. Recker NL, Moore MD. Durkheim, social capital, and suicide rates across US counties. Health Sociology Review (2016) 25:78-91. doi:10.1080/14461242.2015.1101703

71. Rehkopf DH, Buka SL. The association between suicide and the socio-economic characteristics of geographical areas: a systematic review. Psychol Med (2006) 36:145-57. doi:10.1017/S003329170500588X

72. Classen TJ, Dunn RA. The effect of job loss and unemployment duration on suicide risk in the United States: a new look using mass-layoffs and unemployment duration. Health Econ (2012) 21:338-50. doi:10.1002/hec.1719

73. US Department of Labor. Bureau of Labor Statistics, Local Area Unemployment Statistics program (2010). https://download.bls.gov/pub/time.series/la/ [Accessed November 10, 2019].

74. US Census Bureau. Detailed characteristics, 2006-2010 and 2007-2011 American Community Survey 5-year estimates (2011). https://factfinder.census.gov/faces/nav/jsf/pages/searchresults.xhtml?refresh=t [Accessed November 3, 2019].

75. Coleman JC. Social capital in the creation of human capital. American Journal of Sociology (1988) 94: S95-S120.

76. Smith NDL, Kawachi I. State-level social capital and suicide mortality in the 50 U.S. states. Soc Sci Med (2014) 120:269-77. doi:10.1016/j.socscimed.2014.09.007

77. Rupasingha A, Goetz SJ, Freshwater D. The production of social capital in US counties. Journal of Socio-Economics (2006) 35:83-101. doi:10.1016/j.socec.2005.11.001

78. US Census Bureau. (2010). 2010 Census Participation Rates. https://www.census.gov/data/datasets/2010/dec/2010-participation-rates.html [Accessed November 16, 2019].

79. Urban Institute, National Center for Charitable Statistics. Internal Revenue Service, Exempt Organizations Business Master File (2013). https://nccs-data.urban.org [Accessed November 20, 2019].

80. Steelesmith DL, Fontanella CA, Campo JV, Bridge JA, Warren KL, Root ED. Contextual factors associated with county-level suicide rates in the United States, 1999 to 2016. JAMA Netw Open (2019) 2:e1910936. doi:10.1001/jamanetworkopen.2019.10936

81. Meltzer H, Bebbington P, Brugha T, Jenkins R, McManus S, Dennis MS. Personal debt and suicidal ideation. Psychol Med (2011) 41:771-8. doi:10.1017/S0033291710001261

82. Richardson T, Elliott P, Roberts R. The relationship between personal unsecured debt and mental and physical health: a systematic review and meta-analysis. Clin Psychol Rev (2013) 33:1148-62. doi:10.1016/j.cpr.2013.08.009

83. Federal Reserve Bank of New York. Quarterly report on household debt and credit (2018). https://www.federalreserve.gov/releases/z1/dataviz/household_debt/county/map/#state:all;year:2018 [Accessed December 5, 2019].

84. Abel EL, Kruger ML. Educational attainment and suicide rates in the United States. Psychol Rep (2005) 97:25-8. doi:10.2466/pr0.97.1.25-28

85. Evans J, Middleton N, Gunnell D. Social fragmentation, severe mental illness and suicide. Soc Psychiat Epidemiol (2004) 39:165-70. doi:10.1007/s00127-004-0733-9

86. Miller JR. Income inequality and risk of suicide in New York City neighborhoods: a multilevel case-control study. Suicide Life Threat Behav (2005) 35:448-59.

87. Singh G. Area deprivation and widening inequalities in US mortality, 1969–1998. Am J Public Health (2003) 93:1137-43 doi: 10.2105/ajph.93.7.1137

88. Miller M, Swanson SA, Azrael D. Are we missing something pertinent? a bias analysis of unmeasured confounding in the firearm-suicide literature. Epidemiol Rev (2016) 38:62-9. doi:10.1093/epirev/mxv011

89. Walker R, Keane C, Burke J. Disparities and access to healthy food in the United States: a review of food deserts literature. Health Place (2010) 16:876-84. doi:10.1016/j.healthplace.2010.04.013

90. Frutos AM, Sloan CD, Merrill RM. Modeling the effects of atmospheric pressure on suicide rates in the USA using geographically weighted regression. PLoS One (2018) 13:e0206992. doi:10.1371/journal.pone.0206992

91. Ver Ploeg, M., Breneman, V., Farrigan, T., Hamrick, K., Hopkins, D., Kaufman, P., et al. Access to Affordable and Nutritious Food: Measuring and Understanding Food Deserts and Their Consequences: Report to Congress. Washington, DC: Economic Research Service, USDA (2009). Data retrieved from: https://www.ers.usda.gov/data-products/food-access-research-atlas/download-the-data/#Archived%20Versions.

92. Durkheim, E. Suicide: a study in sociology [1897]. Translated by JA Spaulding and G. Simpson. Glencoe, Illinois: The Free Press (1951).

93. Cohen DA, Mason K, Bedimo A, Scribner R, Basolo V, Farley TA. Neighborhood physical conditions and health. Am J Public Health (2003) 93:467-71. doi: 10.2105/ajph.93.3.467

94. Congdon P. Suicide and parasuicide in London: a small-area study. Urban Studies. (1996) 33:137-58. doi:10.1080/00420989650012194

95. Whitley E, Gunnell D, Dorling D, Smith GD. Ecological study of social fragmentation, poverty, and suicide. BMJ (1999) 319:1034-7. doi:https://doi.org/10.1136/bmj.319.7216.1034

96. Bécares L, Dewey ME, Das-Munshi J. Ethnic density effects for adult mental health: systematic review and meta-analysis of international studies. Psychol Med (2018) 48:2054-72. doi:10.1017/S0033291717003580

97. Schofield P, Das-Munshi J, Bécares L, Morgan C, Bhavsar V, Hotopf M, et al. Minority status and mental distress: a comparison of group density effects. Psychol Med (2016) 46:3051-9. doi:10.1017/S0033291716001835

98. Li G. totalcensus: Extract Decennial Census and American Community Survey Data. R package version 0.6.2 (2019). https://CRAN.R-project.org/package=totalcensus [Accessed December 1, 2019]

99 Lester D. A regional analysis of suicide and homicide rates in the USA: search for broad cultural patterns. Soc Psychiat Epidemiol (1988) 23:202-5. doi:10.1007/BF01794789

100. US Department of Justice. Uniform Crime Reporting Program Data: County-Level Detailed Arrest and Offense Data. ICPSR27644-v1. Ann Arbor, MI: Inter-university Consortium for Political and Social Research (2011). https://www.openicpsr.org/openicpsr/project/108164/version/V3/view [Accessed November 30, 2019].

101. Rossen LM, Hedegaard H, Khan D, Warner M. County-level trends in suicide rates in the U.S., 2005–2015. Am J Prev Med (2018) 55:72-9. doi:10.1016/j.amepre.2018.03.020

102. Dwyer-Lindgren L, Bertozzi-Villa A, Stubbs RW, Morozoff C, Kutz MJ, Huynh C, et al. US county-level trends in mortality rates for major causes of death, 1980-2014. JAMA (2016) 316:2385-401. doi:10.1001/jama.2016.13645

103. Institute for Health Metrics and Evaluation (IHME). United States Mortality Rates by County 1980-2014. Seattle, WA: Institute for Health Metrics and Evaluation (IHME) (2016). Data retrieved from: http://ghdx.healthdata.org/us-data.

104. Gagne JJ, Glynn RJ, Avorn J, Levin R, Schneeweiss S. A combined comorbidity score predicted mortality in elderly patients better than existing scores. J Clin Epidemiol (2011) 64:749-59. doi:10.1016/j.jclinepi.2010.10.004

105. Romano PS, Roos LL, Jollis JG. Adapting a clinical comorbidity index for use with ICD-9-CM administrative data: differing perspectives. J Clin Epidemiol (1993) 46:1075-9.

106. Charlson ME, Pompei P, Ales KL, MacKenzie CR. A new method of classifying prognostic comorbidity in longitudinal studies: development and validation. J Chronic Dis (1987) 40:373-83. doi:10.1016/0021-9681(87)90171-8

107. van Walraven C, Austin PC, Jennings A, Quan H, Forster AJ. A modification of the Elixhauser comorbidity measures into a point system for hospital death using administrative data. Med Care (2009) 47:626-33. doi:10.1097/MLR.0b013e31819432e5

108. Elixhauser A, Steiner C, Harris DR, Coffey RM. Comorbidity measures for use with administrative data. Med Care (1998) 36:8-27.

109. Bolton JM, Walld R, Chateau D, Finlayson G, Sareen J. Risk of suicide and suicide attempts associated with physical disorders: a population-based, balancing score-matched analysis. Psychol Med (2015) 45:495-504. doi:10.1017/S0033291714001639

110. Zaorsky NG, Zhang Y, Tuanquin L, Bluethmann SM, Park HS, Chinchilli VM. Suicide among cancer patients. Nat Commun (2019) 10:207. doi:10.1038/s41467-018-08170-1

111. Kam D, Salib A, Gorgy G, Patel TD, Carniol ET, Eloy JA, et al. Incidence of suicide in patients with head and neck cancer. JAMA Otolaryngol Head Neck Surg (2015) 141:1075-81. doi:10.1001/jamaoto.2015.2480

112. Henson KE, Brock R, Charnock J, Wickramasinghe B, Will O, Pitman A. Risk of Suicide after cancer diagnosis in England. JAMA Psychiatry (2018) 76: 51-60. doi:10.1001/jamapsychiatry.2018.3181

113. Saad AM, Gad MM, Al‐Husseini MJ, AlKhayat MA, Rachid A, Alfaar AS, et al. Suicidal death within a year of a cancer diagnosis: a population‐based study. Cancer (2019) 125:972-9. doi:10.1002/cncr.31876

114. Dalela D, Krishna N, Okwara J, Preston MA, Abdollah F, Choueiri TK, et al. Suicide and accidental deaths among patients with non-metastatic prostate cancer. BJU Int (2016) 118:286-97. doi:10.1111/bju.13257

115. Guo Z, Gan S, Li Y, Gu C, Xiang S, Zhou J, et al. Incidence and risk factors of suicide after a prostate cancer diagnosis: a meta-analysis of observational studies. Prostate Cancer Prostatic Dis (2018) 21:499-508. doi:10.1038/s41391-018-0073-6

116. Barker E, Kõlves K, De Leo D. The relationship between asthma and suicidal behaviours: a systematic literature review. Eur Respir J (2015) 46:96-106. doi:10.1183/09031936.00011415

117. Druss B, Pincus H. Suicidal ideation and suicide attempts in general medical illnesses. Arch Intern Med (2000) 160:1522-6. doi:10.1001/archinte.160.10.1522

118. Crump C, Sundquist K, Sundquist J, Winkleby MA. Sociodemographic, psychiatric and somatic risk factors for suicide: a Swedish national cohort study. Psychol Med (2014) 44:279-89. doi:10.1017/S0033291713000810

119. Kuo C-J, Chen VC-H, Lee W-C, Chen WJ, Ferri CP, Stewart R, et al. Asthma and suicide mortality in young people: a 12-year follow-up study. Am J Psychiatry (2010) 167:1092-9. doi:10.1176/appi.ajp.2010.09101455

120. Singhal A, Ross J, Seminog O, Hawton K, Goldacre MJ. Risk of self-harm and suicide in people with specific psychiatric and physical disorders: comparisons between disorders using English national record linkage. J R Soc Med (2014) 107:194-204. doi:10.1177/0141076814522033

121. Chen C-H, Lin C-L, Hsu C-Y, Kao C-H. A retrospective administrative database analysis of suicide attempts and completed suicide in patients with chronic pancreatitis. Front Psychiatry (2018) 9:147. doi:10.3389/fpsyt.2018.00147

122. Roberts SE, John A, Kandalama U, Williams JG, Lyons RA, Lloyd K. Suicide following acute admissions for physical illnesses across England and Wales. Psychol Med (2018) 48:578-91. doi:10.1017/S0033291717001787

123. Wetzel HH, Gehl CR, Dellefave L, Schiffman JF, Shannon KM, Paulsen JS. Suicidal ideation in Huntington disease: the role of comorbidity. Psychiatry Res (2011) 188:372-6. doi:10.1016/j.psychres.2011.05.006

124. Paulsen JS, Hoth KF, Nehl C, Stierman L. Critical periods of suicide risk in Huntington’s disease. Am J Psychiatry (2005) 162:725-31. doi:10.1176/appi.ajp.162.4.725

125. Prabhakar D, Peterson EL, Hu Y, Rossom RC, Lynch FL, Lu CY, et al. Dermatologic conditions and risk of suicide: a case-control study. Psychosomatics (2018) 59:58-61. doi:10.1016/j.psym.2017.08.001

126. Wu JJ, Penfold RB, Primatesta P, Fox TK, Stewart C, Reddy SP, et al. The risk of depression, suicidal ideation and suicide attempt in patients with psoriasis, psoriatic arthritis or ankylosing spondylitis. J Eur Acad Dermatol Venereol (2017) 31:1168-75. doi:10.1111/jdv.14175

127. Parisi R, Webb RT, Kleyn CE, Carr MJ, Kapur N, Griffiths CEM, et al. Psychiatric morbidity and suicidal behaviour in psoriasis: a primary care cohort study. Br J Dermatol (2019) 180:108-15. doi:10.1111/bjd.17004

128. Rodriguez-Bolanos F, Gooderham M, Papp K. A closer look at the data regarding suicidal ideation and behavior in psoriasis patients: the case of brodalumab. Skin Therapy Lett (2019) 24:1-4.

129. Lebwohl MG, Papp KA, Marangell LB, Koo J, Blauvelt A, Gooderham M, et al. Psychiatric adverse events during treatment with brodalumab: analysis of psoriasis clinical trials. J Am Acad Dermatol (2018) 78:81-9.e5. doi:10.1016/j.jaad.2017.08.024

130. Gjervig Hansen H, Köhler-Forsberg O, Petersen L, Nordentoft M, Postolache TT, Erlangsen A, et al. Infections, anti-infective agents, and risk of deliberate self-harm and suicide in a young cohort: a nationwide study. Biol Psychiatry (2019) 85:744-51. doi:10.1016/j.biopsych.2018.11.008

131. Campbell G, Darke S, Bruno R, Degenhardt L. The prevalence and correlates of chronic pain and suicidality in a nationally representative sample. Aust N Z J Psychiatry (2015) 49:803-11. doi:10.1177/0004867415569795

132. Racine M, Sánchez-Rodríguez E, Gálan S, Tomé-Pires C, Solé E, Jensen MP, et al. Factors associated with suicidal ideation in patients with chronic non-cancer pain. Pain Med (2016) 18:283-93. doi:10.1093/pm/pnw115

133. Pergolizzi Jr JV, Passik S, LeQuang JA, Colucci D, Taylor R, B Raffa R, et al. The risk of suicide in chronic pain patients. Nurs Palliat Care (2018) 3. doi:10.15761/NPC.1000189

134. Goulet JL, Kerns RD, Bair M, Becker WC, Brennan P, Burgess DJ, et al. The musculoskeletal diagnosis cohort: examining pain and pain care among veterans. Pain (2016) 157:1696-703. doi:10.1097/j.pain.0000000000000567

135. Mayhew M, DeBar LL, Deyo RA, Kerns RD, Goulet JL, Brandt CA, et al. Development and assessment of a crosswalk between ICD-9-CM and ICD-10-CM to identify patients with common pain conditions. J Pain (2019) 20:1429-45. doi:10.1016/j.jpain.2019.05.006

136. Chronic Pain Research Alliance. Impact of chronic overlapping pain conditions on public health and the urgent need for safe and effective treatment (2015). http://www.chronicpainresearch.org/public/CPRA_WhitePaper_2015-FINAL-Digital.pdf [Accessed December 1, 2019].

137. Maixner W, Fillingim RB, Williams DA, Smith SB, Slade GD. Overlapping chronic pain conditions: implications for diagnosis and classification. J Pain (2016) 17:T93-107. doi:10.1016/j.jpain.2016.06.002

138. Schrepf A, Williams DA, Gallop R, Naliboff B, Basu N, Kaplan C, et al. Sensory sensitivity and symptom severity represent unique dimensions of chronic pain: a MAPP Research Network study. Pain (2018) 159:2002-11. doi:10.1097/j.pain.0000000000001299

139. Crane AM, Levitt RC, Felix ER, Sarantopoulos KD, McClellan AL, Galor A. Patients with more severe symptoms of neuropathic ocular pain report more frequent and severe chronic overlapping pain conditions and psychiatric disease. Br J Ophthalmol (2017) 101:227-31. doi:10.1136/bjophthalmol-2015-308214

140. ICD10Data. Convert ICD-9-CM codes to ICD-10-CM/PCS, or convert ICD-10-CM/PCS codes to ICD-9-CM (2018). https://www.icd10data.com/Convert [Accessed November 27, 2019].

141. Rosellini AJ, Stein MB, Benedek DM, Bliese PD, Chiu WT, Hwang I, et al. Using self-report surveys at the beginning of service to develop multi-outcome risk models for new soldiers in the U.S. Army. Psychol Med (2017) 47:2275-87. doi:10.1017/S003329171700071X

142. Arias Vázquez PI, Castillo Avila RG, Dominguez Zentella MdC, Hernández-Díaz Y, González-Castro TB, Tovilla-Zárate CA, et al. Prevalence and correlations between suicide attempt, depression, substance use, and functionality among patients with limb amputations. Int J Rehabil Res (2018) 41:52-6. doi:10.1097/MRR.0000000000000259

143. Bulzacchelli MT, Sulsky SI, Rodriguez-Monguio R, Karlsson LH, Hill MOT. Injury during U.S. Army basic combat training: a systematic review of risk factor studies. Am J Prev Med (2014) 47:813-22. doi:10.1016/j.amepre.2014.08.008

144. Theodoroff SM, Lewis MS, Folmer RL, Henry JA, Carlson KF. Hearing impairment and tinnitus: prevalence, risk factors, and outcomes in US service members and veterans deployed to the Iraq and Afghanistan wars. Epidemiol Rev (2015) 37:71-85. doi:10.1093/epirev/mxu005

145. Gupta A, Chadda RK. Adverse psychiatric effects of non-psychotropic medications. BJPsych advances (2016) 22:325-34. doi:10.1192/apt.bp.115.015735

146. Gorton HC, Webb RT, Kapur N, Ashcroft DM. Non-psychotropic medication and risk of suicide or attempted suicide: a systematic review. BMJ Open (2016) 6:e009074. doi:10.1136/bmjopen-2015-009074

147. Ambizas EM. Nonpsychotropic medication-induced psychosis. U.S. Pharmacist (2014) 39:HS8-15.

148. Parker C. Psychiatric effects of drugs for other disorders. Medicine (2016) 44:768-74. doi:10.1016/j.mpmed.2016.09.011

149. Casagrande Tango R. Psychiatric side effects of medications prescribed in internal medicine. Dialogues Clin Neurosci (2003) 5:155-65.

150. Turjanski N, Lloyd GG. Psychiatric side-effects of medications: recent developments. Adv Psychiatr Treat (2005) 11:58-70. doi:10.1192/apt.11.1.58

151. Qato DM, Ozenberger K, Olfson M. Prevalence of prescription medications with depression as a potential adverse effect among adults in the United States. JAMA (2018) 319:2289-98. doi:10.1001/jama.2018.6741

152. Winnenburg R, Sorbello A, Bodenreider O. Exploring adverse drug events at the class level. J Biomed Semantics (2015) 6:18. doi:10.1186/s13326-015-0017-1

153. Tariq MM, Streeten EA, Smith HA, Sleemi A, Khabazghazvini B, Vaswani D, et al. Vitamin D: a potential role in reducing suicide risk? Int J Adolesc Med Health (2011) 23:157-65. doi:10.1515/ijamh.2011.038

154. Wu L, Ingle T, Liu Z, Zhao-Wong A, Harris S, Thakkar S, et al. Study of serious adverse drug reactions using FDA-approved drug labeling and MedDRA. BMC Bioinformatics (2019) 20:97. doi:10.1186/s12859-019-2628-5

155. US Food & Drug Administration. CFR - Code of Federal Regulations Title 21, Volume 4 (21CFR201.57) (2019). https://www.accessdata.fda.gov/scripts/cdrh/cfdocs/cfCFR/CFRSearch.cfm?fr=201.57 [Accessed November 21, 2019].

156. Lavigne JE, Au A, Jiang R, Wang Y, Good CP, Glassman P, et al. Utilization of prescription drugs with warnings of suicidal thoughts and behaviours in the USA and the US Department of Veterans Affairs, 2009: drugs with suicide warnings in the USA. J Pharm Health Serv Res (2012) 3:157-63. doi: 10.1111/j.1759-8893.2012.00093.x

157. Lavigne JE, McCarthy MM, Chapman R, Petrilla A, Knox KL. Exposure to prescription drugs labeled for risk of adverse effects of suicidal behavior or ideation among 100 Air Force personnel who died by suicide, 2006-2009. Suicide and Life Threat Behav (2012) 42:561-66. doi: 10.1111/j.1943-278X.2012.00112.x

158. Robertson HT, Allison DB. Drugs associated with more suicidal ideations are also associated with more suicide attempts. PLoS ONE (2009) 4:e7312. doi:10.1371/journal.pone.0007312

159. Adams TD, Halverson RC, LaMonte MJ, Hunt SC. Long-term mortality after gastric bypass surgery. N Engl J Med (2007) 357:753-61.

160. Bhatti JA, Nathens AB, Thiruchelvam D, Grantcharov T, Goldstein BI, Redelmeier DA. Self-harm emergencies after bariatric surgery: a population-based cohort study. JAMA Surg (2016) 151:226-32. doi:10.1001/jamasurg.2015.3414

161. Backman O, Stockeld D, Rasmussen F, Näslund E, Marsk R. Alcohol and substance abuse, depression and suicide attempts after Roux-en-Y gastric bypass surgery. Br J Surg (2016) 103:1336-42. doi:10.1002/bjs.10258

162. Trofimovich L, Skopp NA, Luxton DD, Reger MA. Health care experiences prior to suicide and self-inflicted injury, active component, U.S. Armed Forces, 2001-2010. MSMR (2012) 19:2-6.

163. Méndez-Bustos P, Calati R, Rubio-Ramírez F, Olié E, Courtet P, Lopez-Castroman J. Effectiveness of psychotherapy on suicidal risk: a systematic review of observational studies. Front Psychol (2019) 10:277. doi:10.3389/fpsyg.2019.00277
