# Supplementary material for: Using Administrative Data to Predict Suicide After Psychiatric Hospitalization in the Veterans Health Administration System
Source: Front Psychiatry. 2020 May 6;11:390. doi: 10.3389/fpsyt.2020.00390 (PMC7219514; doi:10.3389/fpsyt.2020.00390)
Supplement: Supplementary file 2 [file Image_1.pdf]

Supplementary Figure 1. ROC curve for the model using random forest (the best single classifiers in the super learner ensemble) developed to predict suicides within 12-months of hospital discharge applied in the holdout sample to predict suicides over each of the 5 time horizons

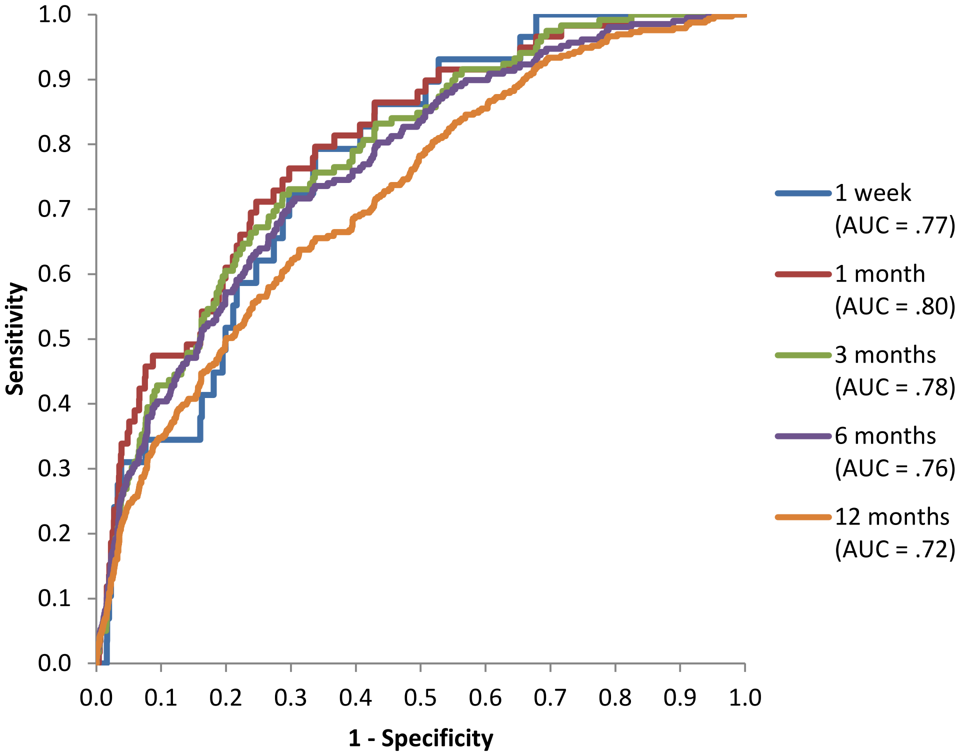

Abbreviations. ROC curve, receiver operating characteristic curve; AUC, area under the curve.
